# Allelic compatibility in plant immune receptors facilitates engineering of new effector recognition specificities

**DOI:** 10.1093/plcell/koad204

**Published:** 2023-07-24

**Authors:** Adam R Bentham, Juan Carlos De la Concepcion, Javier Vega Benjumea, Jiorgos Kourelis, Sally Jones, Melanie Mendel, Jack Stubbs, Clare E M Stevenson, Josephine H R Maidment, Mark Youles, Rafał Zdrzałek, Sophien Kamoun, Mark J Banfield

**Affiliations:** Department of Biochemistry and Metabolism, John Innes Centre, Norwich Research Park, Norwich NR4 7UH, UK; Department of Biochemistry and Metabolism, John Innes Centre, Norwich Research Park, Norwich NR4 7UH, UK; Department of Biochemistry and Metabolism, John Innes Centre, Norwich Research Park, Norwich NR4 7UH, UK; The Sainsbury Laboratory, University of East Anglia, Norwich Research Park, Norwich NR4 7UH, UK; Department of Biochemistry and Metabolism, John Innes Centre, Norwich Research Park, Norwich NR4 7UH, UK; Department of Biochemistry and Metabolism, John Innes Centre, Norwich Research Park, Norwich NR4 7UH, UK; Department of Biochemistry and Metabolism, John Innes Centre, Norwich Research Park, Norwich NR4 7UH, UK; Department of Biochemistry and Metabolism, John Innes Centre, Norwich Research Park, Norwich NR4 7UH, UK; Department of Biochemistry and Metabolism, John Innes Centre, Norwich Research Park, Norwich NR4 7UH, UK; The Sainsbury Laboratory, University of East Anglia, Norwich Research Park, Norwich NR4 7UH, UK; The Sainsbury Laboratory, University of East Anglia, Norwich Research Park, Norwich NR4 7UH, UK; Department of Biochemistry and Metabolism, John Innes Centre, Norwich Research Park, Norwich NR4 7UH, UK; The Sainsbury Laboratory, University of East Anglia, Norwich Research Park, Norwich NR4 7UH, UK; Department of Biochemistry and Metabolism, John Innes Centre, Norwich Research Park, Norwich NR4 7UH, UK

## Abstract

Engineering the plant immune system offers genetic solutions to mitigate crop diseases caused by diverse agriculturally significant pathogens and pests. Modification of intracellular plant immune receptors of the nucleotide-binding leucine-rich repeat (NLR) receptor superfamily for expanded recognition of pathogen virulence proteins (effectors) is a promising approach for engineering disease resistance. However, engineering can cause NLR autoactivation, resulting in constitutive defense responses that are deleterious to the plant. This may be due to plant NLRs associating in highly complex signaling networks that coevolve together, and changes through breeding or genetic modification can generate incompatible combinations, resulting in autoimmune phenotypes. The sensor and helper NLRs of the rice (*Oryza sativa*) NLR pair Pik have coevolved, and mismatching between noncoevolved alleles triggers constitutive activation and cell death. This limits the extent to which protein modifications can be used to engineer pathogen recognition and enhance disease resistance mediated by these NLRs. Here, we dissected incompatibility determinants in the Pik pair in *Nicotiana benthamiana* and found that heavy metal–associated (HMA) domains integrated in Pik-1 not only evolved to bind pathogen effectors but also likely coevolved with other NLR domains to maintain immune homeostasis. This explains why changes in integrated domains can lead to autoactivation. We then used this knowledge to facilitate engineering of new effector recognition specificities, overcoming initial autoimmune penalties. We show that by mismatching alleles of the rice sensor and helper NLRs Pik-1 and Pik-2, we can enable the integration of synthetic domains with novel and enhanced recognition specificities. Taken together, our results reveal a strategy for engineering NLRs, which has the potential to allow an expanded set of integrations and therefore new disease resistance specificities in plants.

## Teaser

Allelic diversity in the rice nucleotide-binding leucine-rich repeat (NLR) receptor pair Pik can be used to expand effector recognition, without autoactivity, thus offering a resource for engineering NLRs to generate disease-resistant crops.

**IIN A NUTSHELL**

**Background:** Plants use specialized intracellular immune receptors called the nucleotide-binding leucine-rich repeat (NLR) receptor to detect pathogen effector proteins secreted into the host during infection. Effectors aid plant colonization, which can result in serious disease and limit crop yields in agriculture. Therefore, immune responses triggered by NLRs upon perception of effectors are essential to maintain plants being healthy. However, plant pathogens use hundreds of effectors during infection and these effectors evolve rapidly, escaping from recognition by NLR immune proteins. Recently, bioengineering of NLRs to recognize a wider range of effectors was demonstrated to be an effective means of generating disease-resistant plants. However, bioengineering of NLRs can result in “autoactivity” where the immune system is constantly active. This is deleterious to the health of the plant.

**Question:** In our study, we strived to understand the limits of bioengineering for a pair of rice NLRs called Pik. Piks are interesting NLRs as they comprise a sensor NLR (important for effector recognition) and a helper NLR (important for defense signaling).

**Findings:** We found bioengineering of the native Pik sensor NLR often resulted in autoactivity. However, by using different combinations of Pik helper NLR alleles (an alternative form of the gene), we could mitigate the autoactivity caused by bioengineering, allowing us to generate Pik sensor NLRs with different effector recognition specificities in the model plant *Nicotiana benthamiana*. Our study establishes a strategy to incorporate a wider variety of effector recognition modules into the Pik NLRs without autoactivity.

**Next steps:** The next step in this research is to understand whether this bioengineered resistance is transferable from model plants to stable transgenic crops, such as rice (*Oryza sativa*), barley (*Hordeum vulgare*), wheat (*Triticum aestivum*), or maize (*Zea mays*), and to understand which pathogens are the best targets for new disease resistance using the Pik NLRs.

## Introduction

Engineering the plant immune system is a promising genetic solution to prevent pathogen infection thereby reducing crop losses and global food insecurity caused by plant pathogens (Bentham et al. 2020; Outram et al. 2022). Nucleotide-binding leucine-rich repeat (NLRs) receptors are intracellular immune proteins that trigger robust defense responses upon recognition of pathogen virulence proteins (effectors) delivered into the host during infection (Jones et al. 2016; Burdett et al. 2019). Effector recognition by NLRs often culminates in cell death that isolates the invading pathogen and confers resistance (Jones et al. 2016; Maruta et al. 2022). Due to their effective and specific responses to plant pathogens, engineering of NLRs to increase their effector recognition specificities is a promising approach to boost disease resistance (Monteiro and Nishimura 2018; Marchal et al. 2022; Outram et al. 2022).

NLRs are typically modular tripartite proteins that consist of an N-terminal signaling domain, either a coiled-coil (CC) domain, a CC domain with homology to RPW8 (CC_R_) or Toll-interleukin-1 receptor (TIR) domain, a central nucleotide-binding (NB) domain, and a C-terminal leucine-rich repeat (LRR) domain (Takken and Goverse 2012; Jones et al. 2016; Lüdke et al. 2022). NLR proteins can function as singletons, in pairs and in networks, and utilize several mechanisms to detect and respond to pathogen effectors (Cesari 2018; Wu et al. 2018; Adachi et al. 2019). One such mechanism is the use of integrated domains, which function as effector baits embedded within the canonical NLR architecture (Cesari et al. 2014; Baggs et al. 2017). Integrated domains often share homology to pathogen–host targets and effector binding results in NLR activation (Kroj et al. 2016; Sarris et al. 2016; Białas et al. 2018; Cesari 2018). Due to their role in effector recognition, integrated domains are key targets for engineering disease resistance in NLRs, and only a few mutations to these domains can lead to novel effector recognition profiles (De la Concepcion et al. 2019; Liu et al. 2021; Cesari et al. 2022; Maidment et al. 2022; Zhang et al. 2022).

Most characterized NLRs that contain integrated domains (NLR-IDs) are found as a part of a sensor/helper receptor pair, where the NLR-ID is referred to as the sensor, and its signaling partner, the helper (Cesari 2018; Adachi et al. 2019; Feehan et al. 2020). Some of the best-characterized examples of paired NLRs are the *Arabidopsis* (*Arabidopsis thaliana*) RESISTANCE TO *RALSTONIA SOLANACEARUM* 1 (RRS1)/RESISTANCE TO PSEUDOMONAS SYRINGAE 4 (RPS4) pairs that encode an RRS1-integrated WRKY domain (Le Roux et al. 2015; Zhang et al. 2017; Mukhi et al. 2021) and the rice (*Oryza sativa*) NLR pairs RGA5/RGA4 and Pik-1/Pik-2 (Kanzaki et al. 2012; Cesari et al. 2013; Zdrzałek et al. 2020). The RGA5/RGA4 and Pik pairs harbor an integrated heavy metal-associated (HMA) domain in their sensor NLRs RGA5 and Pik-1 that directly bind and recognize MAX (*Magnaporthe oryzae* avirulence and ToxB like) effectors (Maqbool et al. 2015; Guo et al. 2018). While both RGA5 and Pik-1 contain an integrated HMA domain, their domain architecture is distinct with the Pik-1 HMA domain located between the CC and NB domains and the RGA5 HMA domain located at the C-terminus, after the LRR (Kanzaki et al. 2012; Cesari et al. 2013; Maqbool et al. 2015; Ortiz et al. 2017). Further, these HMA domains provide distinct effector recognition specificities for AVR-Pik, AVR-Mgk1, and AVR-Pia/AVR1-CO39 effectors, respectively (Białas et al. 2018; Sugihara et al. 2022).

The HMA domains of Pik-1 and RGA5 use spatially distinct protein interfaces for effector recognition (De la Concepcion et al. 2018; De la Concepcion, Maidment, et al. 2021; Guo et al. 2018; Varden et al. 2019). Recent studies have reported HMA domain engineering to be an effective way to generate new resistance specificities for rice against the rice blast pathogen *M. oryzae*. In particular, 3 separate studies have shown the RGA5 HMA domain can be engineered to recognize other MAX effectors. One study showed engineered resistance to *M. oryzae* isolates carrying AVR-Pib in rice (Liu et al. 2021), and 2 studies engineered recognition of AVR-Pik in *Nicotiana benthamiana*, 1 of which was able to provide resistance in rice (Cesari et al. 2022; Zhang et al. 2022). Extensive study of the Pik-1 HMA domain has also demonstrated this domain to be amenable to engineering (De la Concepcion et al. 2019; De la Concepcion, Maidment, et al. 2021; Maidment et al. 2022). Recently, it has been shown the Pik-1 HMA can be substituted for VHH nanobody fusions that act as synthetic effector recognition domains, demonstrating the flexibility of the Pik system for mutation or substitution of new integrated domains (Kourelis et al. 2023).

Despite some success, plant immune receptor engineering remains challenging. NLRs exist in complex, regulated systems, and, as a consequence, some changes in NLRs that expand recognition can also result in constitutive defense signaling that is deleterious for plant growth (Białas et al. 2021; Maidment et al. 2022; Tamborski et al. 2023). In particular, the manipulation of integrated domains in paired NLRs often results in autoactivity. This is best exemplified by several studies of the Pik NLR pair, in which the integrated domain of the Pik-1 sensor was substituted for an engineered variant or entirely different domains, resulting in autoactivation of the receptor pair (Białas et al. 2021; Maidment et al. 2022; Kourelis et al. 2023). NLR-mediated autoimmunity has been well documented, with hybrid necrosis phenotypes as a result of crosses linked to incompatible pairing of NLRs (Bomblies et al. 2007; Chae et al. 2014; Tran et al. 2017; Kourelis and Adachi 2022), presenting a bottleneck to producing new resistant crop varieties either by breeding or precision protein engineering.

Recently, we demonstrated alleles of the rice Pik NLR pair have differentially coevolved, likely driven by their differences in recognition specificity for *M. oryzae* AVR-Pik effector variants (De la Concepcion, Vega Benjumea, et al. 2021). The Pik alleles Pikp and Pikm have undergone functional diversification, with multiple changes in their integrated HMA domain that result in different recognition specificities for AVR-Pik variants (Białas et al. 2021; De la Concepcion, Maidment, et al. 2021; De la Concepcion, Vega Benjumea, et al. 2021). Where the Pikp-1 sensor is restricted to detecting AVR-PikD, Pikm-1 is able to recognize AVR-PikD, AVR-PikE, and AVR-PikA (Kanzaki et al. 2012; De la Concepcion et al. 2018). The helper NLRs Pikp-2 and Pikm-2 also appear to have undergone diversification to match their sensor partners that results in a 1-way incompatibility between Pik alleles (De la Concepcion, Vega Benjumea, et al. 2021). While Pikp-2 can be used as a helper with Pikm-1 to recognize AVR-Pik effectors, the Pikp-1/Pikm-2 combination results in constitutive cell death in *N. benthamiana*. This incompatibility between Pikp-1 and Pikm-2 is linked to a single polymorphism in the NB-ARC of Pikm-2, which when mutated to the equivalent residue of Pikp-2 reinstates compatibility (De la Concepcion, Vega Benjumea, et al. 2021).

Here, we demonstrate the autoactivity triggered by the engineering of Pikm-1 for expanded effector recognition capabilities can be attenuated by the coexpression with Pikp-2 without compromising receptor function. For this, we delineate the basis for receptor incompatibility between Pikp and Pikm alleles, describing Pikp-2 as a facilitator for integration of new integrated domains into Pikm-1. By mismatching Pikm-1 with Pikp-2, we can integrate the RGA5 HMA domain into Pikm-1, enabling further engineering of RGA5 HMA to recognize multiple AVR-Pik effector variants. We structurally and biophysically characterize the interaction between a synthetic AVR-Pik-binding (APB) mutant of RGA5 HMA and AVR-Pik effectors, highlighting the importance of binding affinity between effector and bait for immune recognition. As a final demonstration of the utility of helper mismatching, we demonstrate this strategy also allows for the integration of fluorescent protein (FP)-binding nanobodies into the Pikm-1 chassis without autoactivation, resulting in synthetic receptors capable of responding to eGFP or mCherry in planta. These results emphasize the importance of the immune receptor context when attempting NLR engineering, supplying an alternative approach to aid in the implementation of modified immune receptors with expanded effector recognition specificities outside of that previously observed in nature.

## Results

### The Pik-HMA domain is not required for effector-independent immune signaling

**Figure 1.**
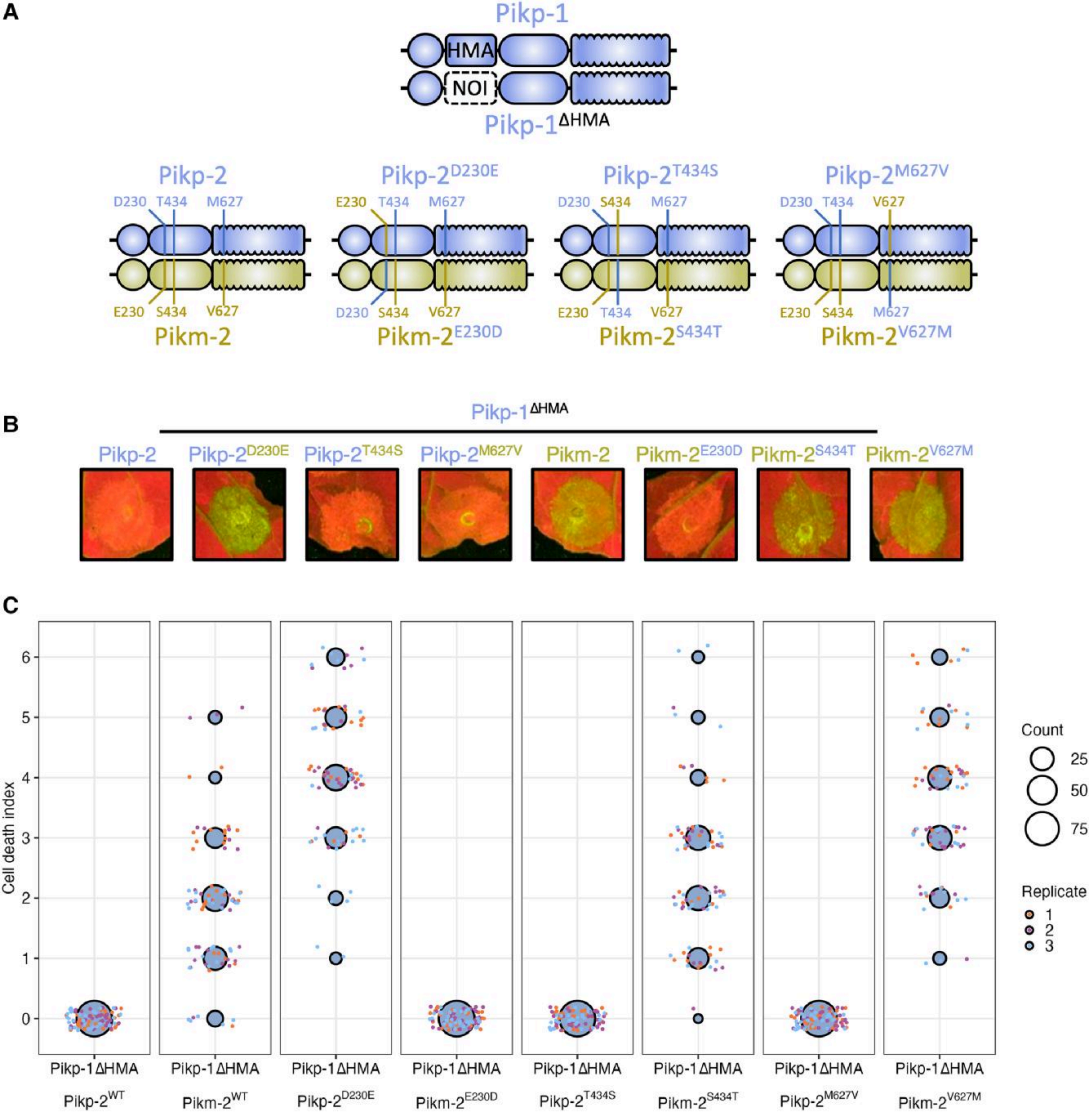
Pik-2 polymorphisms determine Pik-1 HMA domain independent allelic compatibility. **A)** Schematic diagram highlighting the domain architecture of Pikp-1 and Pikp-1^ΔHMA^ sensor NLRs and the polymorphisms between the Pikp-2 and Pikm-2 helper NLRs. **B)***N. benthamiana* cell death assays of Pikp-1^ΔHMA^ coexpressed with Pik-2 helpers and mutant variants visualized via ultraviolet light. The HMA domain of Pikp-1 is not required for effector-independent cell death in *N. benthamiana*; compatibility remains with Pikp-2 but not Pikm-2. Reciprocal D230E and E230D mutations in Pikp-2 and Pikm-2 flip the compatibility of the helper NLRs for Pikp-1^ΔHMA^. The other polymorphic residues between Pikp-2 and Pikm-2, T434S and M627V, have no effect on helper compatibility. **C)** Cell death scoring for repeats of Pikp-1^ΔHMA^ coexpressed with Pikp-2, Pikm-2, and mutants in *N. benthamiana* represented as dot plots. The total number of repeats was spots 75 per sample. For each sample, all the data points are represented as dots with a distinct color for each of the 3 biological replicates; these dots are jittered around the cell death score for visualization purposes. The size of the central dot at each cell death value is proportional to the number of replicates of the sample with that score. Quantification and statistical analysis of these results can be found in Supplemental Appendix S1A. Details of the NLR mutants used in these experiments can be found in Supplemental Table S3.

The mismatched pairing of the Pikp-1/Pikm-2 alleles triggers constitutive cell death in the absence of an effector binding to the integrated HMA domain (De la Concepcion, Vega Benjumea, et al. 2021). To better assess the role of the Pik HMA domain in signaling, activation, and autoimmunity outside of effector binding, we used an HMA-absent Pikp-1 variant (Pikp-1^ΔHMA^) where the HMA domain of Pikp-1 was substituted with the unrelated NOI domain from the rice NLR Pii-2 (Pii-2 residues Glu1016 to Lys1052) (Fujisaki et al. 2017). Constitutive cell death was observed upon coexpression of Pikp-1^ΔHMA^ with Pikm-2 in *N. benthamiana*. However, like wild-type Pikp-1, coexpression of Pikp-1^ΔHMA^ with Pikp-2 did not result in cell death (Fig. 1 and Supplemental Appendix S1A).

The Pikp-2 and Pikm-2 helpers only differ by 3 polymorphisms: Asp230Glu, Thr434Ser, and Met627Val. We made reciprocal mutants of Pikp-2 and Pikm-2 helpers for each of these polymorphisms, generating 6 mutants (Pikp-2^D230E^, Pikp-2^T434S^, Pikp-2^M627V^, Pikm-2^E230D^, Pikm-2^S434T^, and Pikm-2^V627M^), and found autoactivity induced by coexpression of Pikp-1^ΔHMA^/Pikm-2 is determined by the Pik-2 Asp230Glu polymorphism (Pikp-2^D230E^/Pikm-2^E230D^) (Fig. 1 and Supplemental Appendix S1A) as previously described for wild-type Pik NLRs (De la Concepcion, Vega Benjumea, et al. 2021). An Asp230Glu mutation into Pikp-2 resulted in constitutive activation when coexpressed with Pikp-1^ΔHMA^, and the reciprocal mutation, Glu230Asp, in Pikm-2 abolished autoactivity (Fig. 1 and Supplemental Appendix S1A). This suggests the integrated HMA domain acts as an effector-binding domain but is not required for downstream NLR signaling and cell death.

### Incompatibility between alleles of the Pik NLR pair is linked to regions within the sensor and the helper

**Figure 2.**
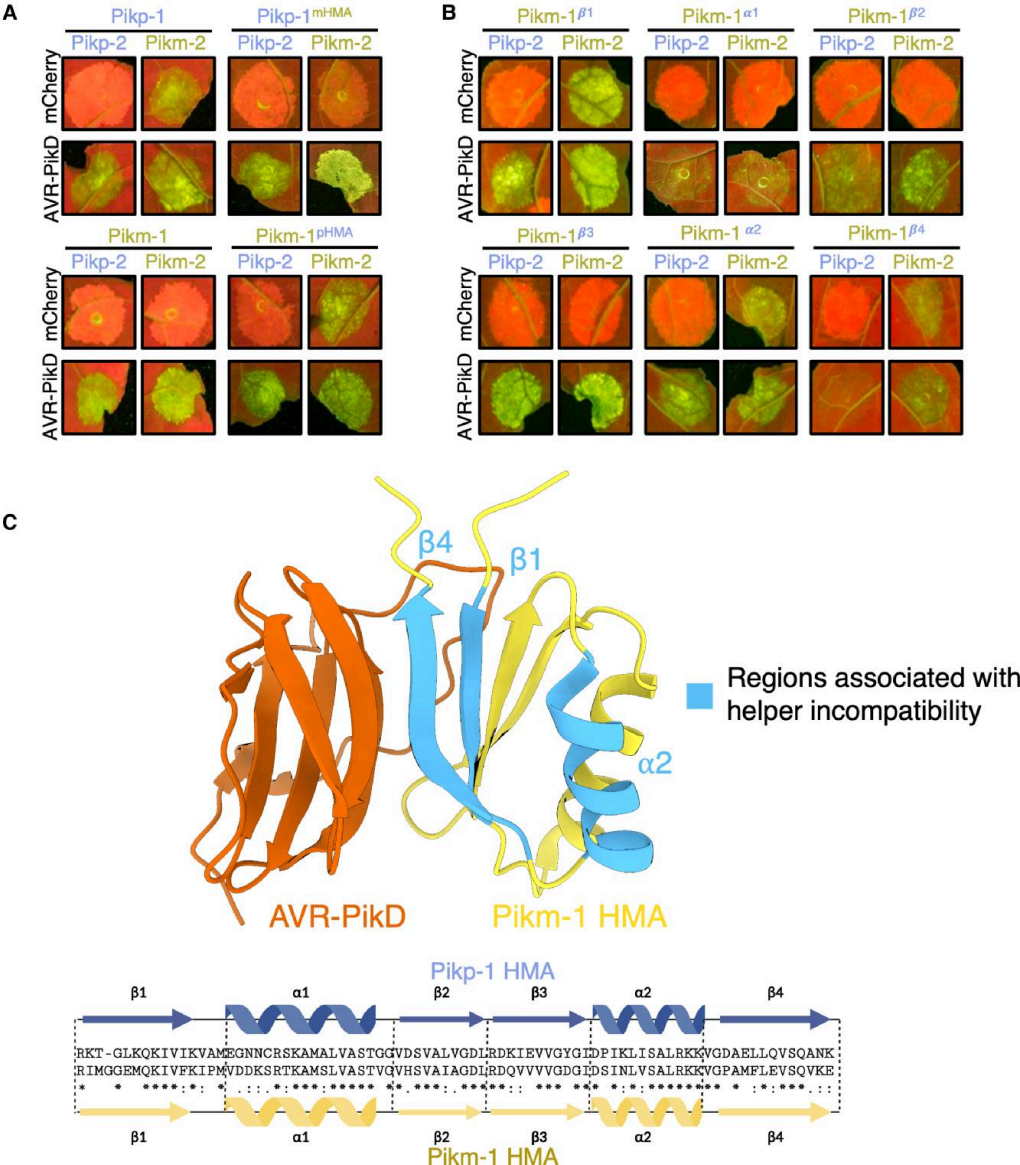
The HMA domain of Pik-1 is important for compatibility with Pik-2 helpers. **A)** Coexpression of Pikp-1 with Pikm-2 triggers effector-independent cell death in *N. benthamiana.* Integration of the Pikm-1 HMA into Pikp-1 facilitates Pikp-1 compatibility with Pikm-2, whereas incorporation of the Pikp-1 HMA into Pikm-1 abolishes compatibility with Pikm-2. Quantification and statistical analysis of these results are shown in Supplemental Fig. S1A and Appendix S1B. **B)** Incompatibility of the Pikp-1 with Pikm-2 in *N. benthamiana* is linked to the α2 helix, *β*1, and *β*4 strands of the HMA domain, with Pikm-1 HMA chimeras carrying the Pikp-1 secondary structure elements resulting in effector-independent cell death when coexpressed with Pikm-2. Quantification and statistical analysis of these results can be found in Supplemental Fig. S1B and Appendix S1C. **C)** Some regions of the HMA domain that are involved in sensor/helper incompatibility are shared with the APB interface (PDB ID: 6FU9). Details of the NLR mutants used in these experiments can be found in Supplemental Table S3.

As the integrated HMA is not required for immune activation of the Pik pair and is the most variable domain between the Pikp-1 and Pikm-1 alleles (Costanzo and Jia 2010), we hypothesized the Pikm-HMA domain coevolved with Pikm-2 to suppress autoactivation mediated by Pik-2 Asp230Glu polymorphism. To test this, we exchanged the integrated HMA domains between sensor alleles Pikp-1 (pHMA) and Pikm-1 (mHMA), to create Pikp-1^mHMA^ and Pikm-1^pHMA^. Pikp-1^mHMA^ and Pikm-1^pHMA^ were coexpressed in *N. benthamiana* with either the Pikp-2 or Pikm-2 helper and challenged with AVR-PikD or mCherry to test for effector activation and autoimmunity, respectively (Figs. 2A and S1A and Supplemental Appendix S1B). Expression of the Pikm-1^pHMA^ with Pikm-2 resulted in effector-independent cell death; however, this sensor was not autoactive in the presence of Pikp-2 and was able to respond to AVR-PikD. By contrast, Pikp-1^mHMA^ was not autoactive when coexpressed with either the Pikp-2 or Pikm-2 helpers and cooperated with either helper to respond to AVR-PikD. These data demonstrate the integrated domain of the Pik-1 sensor contributes to the compatibility between the Pik sensor and helper NLRs.

To gain a better understanding of which features of the HMA domain are involved in sensor/helper compatibility, we generated chimeras by introducing secondary structures from the Pikp-1 HMA into the Pikm-1 HMA and tested for autoactivation in the presence of Pikm-2. The Pik HMA maintains a 4-strand *β*-sandwich fold (*β*1–*β*4) flanked by 2 helices (α1 and α2) (De la Concepcion et al. 2018). For this experiment, we generated 6 chimeric sensors: Pikm-1*^β^*^1^, Pikm-1^α1^, Pikm-1*^β^*^2^, Pikm-1*^β^*^3^, Pikm-1^α2^, and Pikm-1*^β^*^4^, and these were coexpressed with Pikm-2 and AVR-PikD or mCherry in *N. benthamiana* (Figs. 2B and S1B and Supplemental Appendix S1C). Of the 6 mutants, only Pikm-1*^β^*^1^, Pikm-1^α2^, and Pikm-1*^β^*^4^ resulted in effector-independent cell death. However, not all the residues of the *β*1 and *β*4 strands make significant contributions to the APB interface of the HMA (Figs. 2C, S2, and S3 and Supplemental Appendix S1, D to F), implying that some residues not directly involved in the binding to the effector can have a regulatory role in NLR activation. Furthermore, we note coexpression of Pikp-2 with the Pikm-1*^β^*^4^ prevented autoactivity caused by coexpression of this chimera with Pikm-2; however, we observed the Pikm-1*^β^*^4^ to have a reduced response to AVR-PikD when paired with Pikp-2 (Figs. 2B and S1B and Supplemental Appendix S1C). This result may be due to changes to the important interactions between the residues of the *β*4 strand with the effector, which are known to be more extensive in Pikm-1 than in Pikp-1 (De la Concepcion et al. 2018), or potentially due to the reduced sensitivity of Pikp-2 as a helper (De la Concepcion et al., Maidment, 2021; De la Concepcion, Vega Benjumea, et al. 2021).

Following the observation that the *β*1, α2, and *β*4 HMA secondary structures may be involved in helper incompatibility, we created single point mutations of the polymorphic residues between Pikp and Pikm HMA domains in these secondary structures (Supplemental Figs. S2 and S3 and Appendix S1, D to F) to assess their individual contributions to sensor/helper compatibility. We generated 3 sets of single mutants in Pikm-1: the *β*1 mutants Pikm-1^I184K^, Pikm-1^M185T^, Pikm-1^ΔG186^, Pikm-1^E188L^, Pikm-^1M189K,^ Pikm-1^F194I^, Pikm-1^I196V^ and Pikm-1^P197A^; the α2 mutants Pikm-1^S239P^, Pikm-1^N241K^, and Pikm-1^V243I^; and the *β*4 mutants Pikm-1^P252D^, Pikm-1^M254E^, Pikm-1^F255L^, Pikm-1^E257Q^, Pikm-1^V261A^, Pikm-1^K262N^, and Pikm-1^E263K^.

These mutants were then coexpressed in *N. benthamiana* with Pikm-2 to test their effect on receptor compatibility. We observed few of the single Pikm-1 mutants to influence compatibility with Pikm-2 in contrast to our observations with the α2, *β*1, and *β*4 chimeras, which points toward a certain threshold for change in the HMA being tolerated by the system (Supplemental Figs. S2 and S3 and Appendix S1, D to F). Notable exceptions to this were the deletion of Gly186 in *β*1 and the Pro252Asp substitution in *β*4, which resulted in strong autoactivity in the presence of Pikm-2. Why these 2 mutations result in such strong autoactivity is unclear, but could be related to both causing large-scale structural changes, as the removal of a residue (ΔG186) or mutation of a proline (Pro252) could impact secondary structure formation and affect the ability of the mHMA to prevent autoactivity in the presence of Pikm-2.

Taken together, these data demonstrate that Pik-1 integrated domain contributes to compatibility with Pik-2 helper NLR and not only to effector binding, as the HMA domain is not required for cell death signaling but has evolved to accommodate for changes in Pik-2 that would otherwise result in constitutive activation.

### Integration of the RGA5 HMA domain into Pik-1 is facilitated by allelic mismatching

Our results also suggest Pikp-2 may be more accommodating of changes in the Pik-1 sensor than Pikm-2, even tolerating the complete substitution of the integrated HMA by an unrelated domain without inducing autoactivity (Fig. 1 and Supplemental Appendix S1A). We hypothesized the ability of Pikp-2 to accommodate changes in the integrated domain would allow for integration of an HMA domain that would normally result in autoactivity. To test this, we made a chimera of Pikm-1 carrying the HMA domain from the rice NLR RGA5. Using multiple sequence alignment and structural visualization in ChimeraX (Pettersen et al. 2021), we defined residues 997 to 1071 from the RGA5 HMA to be the identical boundaries of the Pikm-1 HMA domain. It was important to make sure the size of the HMA incorporated into the Pikm-1 chassis was identical to the Pikm-1 HMA due to our previous observation that removal of a single residue from the HMA (ΔG186) resulted in strong autoactivity.

**Figure 3.**
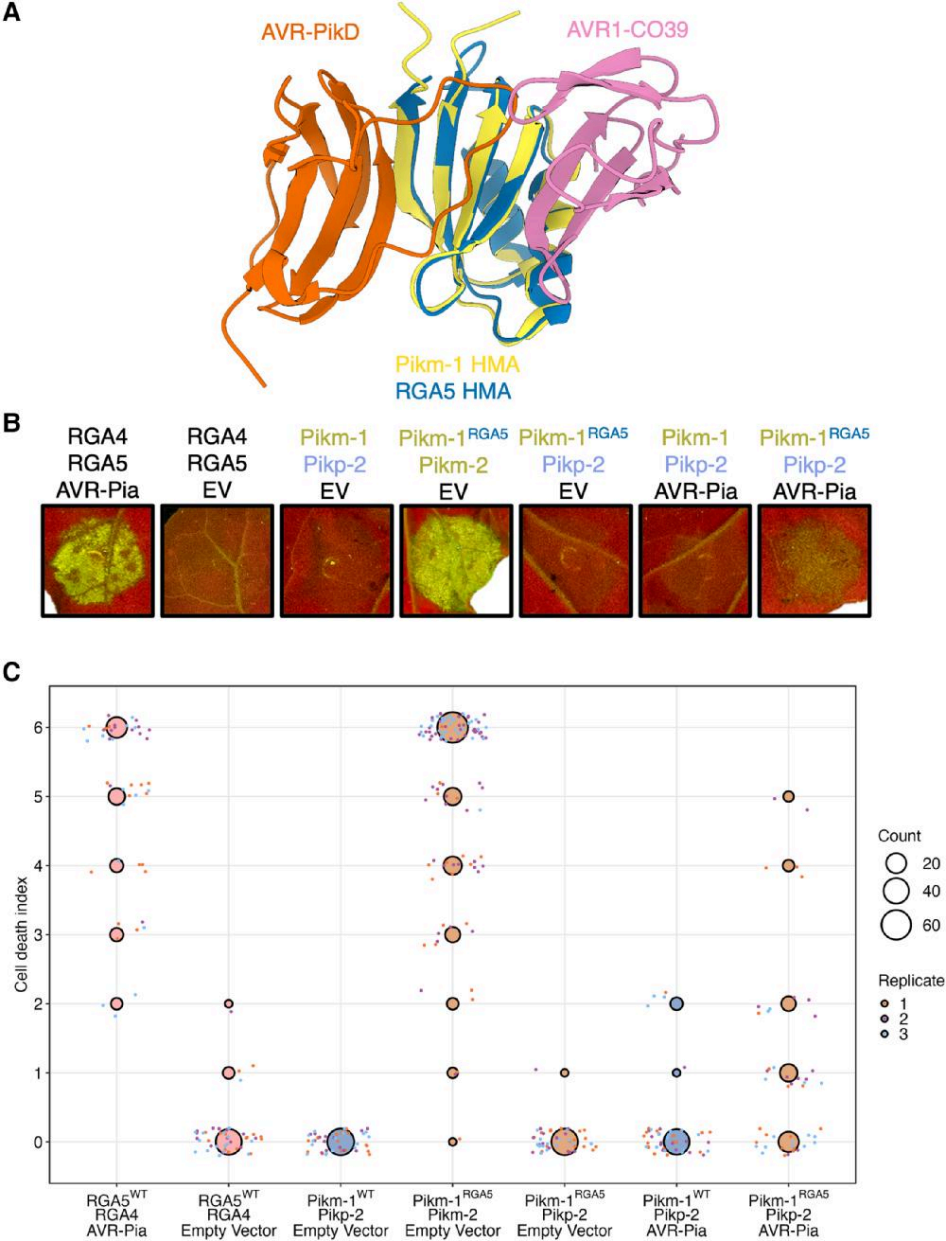
Autoactivity following integration of the RGA5 HMA domain into Pikm-1 is relieved by allelic mismatch with Pikp-2 but only weakly responds to AVR-Pia. **A)** Schematic structural alignment of the RGA5 HMA domain (PDB ID: 5ZNG) with the Pikm-1 HMA domain (PBD ID: 6FU9) showing the different binding interfaces of these HMAs for the AVR-Pik and AVR1-CO39/AVR-Pia (not shown) effectors. **B)** Coexpression of Pikm-1^RGA5^ with Pikp-2 suppresses effector-independent cell death in the presence of empty vector (EV) control and responds weakly to AVR-Pia. **C)** Cell death scoring of wild-type (WT) Pikm-1 and Pikm-1^RGA5^ coexpressed with Pikp-2 in *N. benthamiana* represented as dot plots. The total number of repeats was 45 per sample. For each sample, all the data points are represented as dots with a distinct color for each of the 3 biological replicates; these dots are jittered around the cell death score for visualization purposes. The size of the central dot at each cell death value is proportional to the number of replicates of the sample with that score. Statistical analyses of these results are shown in Supplemental Appendix S1G. Details of the NLR mutants used in these experiments can be found in Supplemental Table S3.

Coexpression of the Pikm-1^RGA5^ chimera with Pikm-2 in *N. benthamiana* resulted in a strong effector-independent cell death response. However, no cell death was observed upon coexpression of Pikm-1^RGA5^ with Pikp-2 (Fig. 3 and Supplemental Appendix S1G). Therefore, the Pikp-2 helper allows the integration of the RGA5 HMA into Pikm-1. To test whether the Pikm-1^RGA5^ chimera is a functional receptor, the Pikm-1^RGA5^/Pikp-2 combination was coexpressed with AVR-Pia. Coexpression of Pikm-1^RGA5^, Pikp-2, and AVR-Pia in *N. benthamiana* resulted in weak cell death, significantly weaker than the cell death elicited by RGA4/RGA5 in response to AVR-Pia (Fig. 3 and Supplemental Appendix S1G), but comparable to the cross-reactivity of Pikp-1/Pikp-2 with AVR-Pia previously observed in *N. benthamiana* (Varden et al. 2019), indicating a level of cell death equivalent to a known interaction, which does not translate into full resistance to *M. oryzae* carrying AVR-Pia in rice plants. Furthermore, we sought determine whether the RGA5 HMA integrated into Pikm-1 could complement the function of the Pikm-1 HMA and respond to AVR-PikD (Supplemental Fig. S4 and Appendix S1H). Coexpression of Pikm-1^RGA5^/Pikp-2 with AVR-PikD did not trigger a cell death response in *N. benthamiana*, demonstrating the RGA5 HMA cannot substitute the Pikm-1 HMA as an AVR-Pik recognition module when integrated into Pikm-1.

These data demonstrate the Pikp-2 helper can be used to facilitate the integration of new domains into the Pikm-1 sensor that would otherwise result in autoactivity/incompatibility when paired with Pikm-2.

### The RGA5 HMA domain can be engineered to recognize AVR-Pik from within the Pik-1 chassis

**Figure 4.**
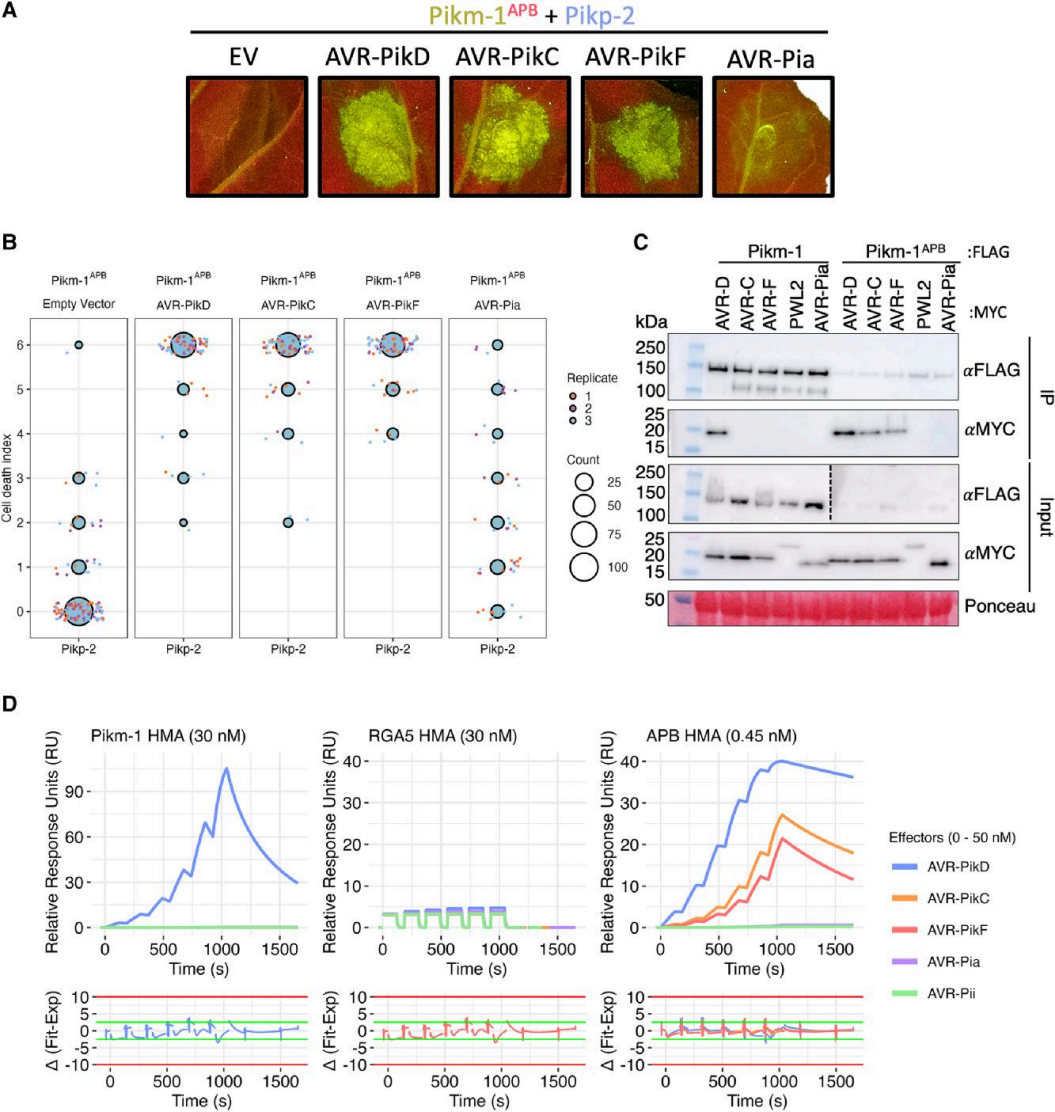
The RGA5 APB mutant binds and recognizes AVR-Pik when integrated into Pikm-1. **A)** Pikm-1^APB^ chimera responds to all variants of AVR-Pik tested and activates cell death when coexpressed with Pikp-2 in *N. benthamiana*, but like Pikm-1^RGA5^, it only weakly responds to AVR-Pia. **B)** Cell death scoring of Pikm-1^APB^ coexpressed with AVR-Pik variants D, C, and F in *N. benthamiana* represented as dot plots. The total number of repeats was 80 per sample. For each sample, all the data points are represented as dots with a distinct color for each of the 3 biological replicates; these dots are jittered around the cell death score for visualization purposes. The size of the central dot at each cell death value is proportional to the number of replicates of the sample with that score. Statistical analyses of these results are shown in Supplemental Appendix S1I. **C)** co-IP of Pikm-1^APB^ with different MAX (*Magnaporthe* AVRs and ToxB-like) effectors shows association with AVR-Pik variants, but not AVR-Pia, in planta. Dotted line denotes separate membrane exposures of the same membrane. **D)** SPR sensograms for the interaction of HMA domains of Pikm-1, RGA5, and RGA5 APB mutant with effectors AVR-PikD, AVR-PikC, AVR-PikF, and AVR-Pia. Non-MAX effector AVR-Pii was added as a negative control. RUs for each labeled protein concentration are shown with the residuals plot beneath (SPR acceptance guides as determined by Biacore software are shown as green and red lines in the residuals plots). Concentration of each protein in the assay is indicated next to their corresponding name. Each experiment was repeated a minimum of 3 times, with similar results.

To test whether the RGA5 HMA can act as an effector recognition module in the Pikm-1 receptor, we engineered AVR-Pik recognition in the RGA5 HMA. Using a host target of AVR-Pik, *O. sativa* heavy metal-associated isoprenylated plant protein 19 (OsHIPP19) (Maidment et al. 2021) as a structural template for APB, we generated an RGA5 variant, termed the APB mutant (Supplemental Fig. S5). The APB mutant contains the point mutations Glu1033Asp, Val1039Gln, Met1065Gln, Leu1068Glu, Glu1070Lys, and Lys1071Glu that localize to a potential APB interface of the RGA5 HMA. Next, we generated a Pikm-1 chimera containing the RGA5-APB mutant HMA (Pikm-1^APB^) and coexpressed it with Pikp-2 and the AVR-Pik variants AVR-PikD, AVR-PikC, and AVR-PikF in *N. benthamiana.* The Pikm-1^APB^ chimera was able to trigger cell death in response to AVR-PikD, AVR-PikC, and AVR-F (Fig. 4, A and B, and Supplemental Appendix S1I). We also tested whether Pikm-1^APB^ could recognize AVR-Pia. However, as for the Pikm-1^RGA5^ chimera, we only observed a weak cell death response (Fig. 4, A and B, and Supplemental Appendix S1I).

To observe whether cell death corresponds with effector binding in planta, we performed coimmunoprecipitation (co-IP) assays with FLAG-tagged Pikm-1^APB^ and 4xMYC-tagged AVR-PikD, AVR-PikC, AVR-PikF, AVR-Pia, and Pathogenicity toward Weeping Lovegrass 2 (PWL2) as negative control. We observed bands corresponding to AVR-PikD, AVR-PikC, and AVR-PikF in Pikm-1^APB^ samples after FLAG pull-down, whereas only AVR-PikD was pulled down by wild-type Pikm-1 (Fig. 4C). Corresponding with the weak response in cell death assays, we were unable to observe association between Pikm-1^APB^ and AVR-Pia.

Taken together, these data demonstrate the RGA5 HMA domain can be engineered to respond to AVR-Pik in planta in the context of the Pikm-1 receptor. However, incorporation of the RGA5 HMA in the Pikm-1 chassis is not sufficient for robust recognition of AVR-Pia.

### The affinity of HMA domains for effectors underpins recognition phenotypes in Pik-1 chimeras

We hypothesized a lower affinity of the APB HMA for AVR-Pia compared to AVR-Pik is responsible for the differences in cell death phenotypes. The interaction between AVR-Pia/RGA5-HMA is known to be much weaker when compared with AVR-Pik/Pik-HMA (Ortiz et al. 2017; De la Concepcion et al. 2018; Guo et al. 2018). To investigate this hypothesis, we performed surface plasmon resonance (SPR) to determine affinities of the RGA5, Pikm-1, and APB HMA domains for different MAX effectors.

We purified AVR-PikD, AVR-PikC, AVR-PikF, AVR-Pia, and the non-MAX effector AVR-Pii as previously described (De la Concepcion et al. 2018, 2022) (see Materials and methods) and performed multicycle kinetics by flowing the effectors over a Biacore CM5 chip presenting amine-coupled RGA5, APB, and Pikm-1 HMA domains (Supplemental Fig. S6 and Table S1). As in previous reports, we observed strong binding of AVR-PikD to the Pikm-1 HMA (equilibrium dissociation constant [*K*_D_] = ∼10 nM) (De la Concepcion et al. 2018) and very low binding to RGA5 HMA (Cesari et al. 2022; Zhang et al. 2022) (Figs. 4D and S6); neither the Pikm-1 HMA nor RGA5 demonstrated any strong binding to AVR-PikC or AVR-PikF, as characterized by their rapid dissociation from the HMA (Supplemental Fig. S6A and Table S1).

Due to the weak binding of AVR-Pia to the HMAs relative to the Pikm-1/AVR-PikD interaction, higher concentrations (up to 50 *µ*M) of AVR-Pia were flowed over the chip, which allowed us to measure the affinity of AVR-Pia for the RGA5 and APB HMAs at 26.8 and 32.9 *µ*M, respectively (Supplemental Fig. S6). These values are in agreement with previous studies that have reported micromolar affinities between the RGA5 HMA and AVR1-CO39 and AVR-Pia effectors using isothermal titration calorimetry (ITC) or microscale thermophoresis (MST) (Ortiz et al. 2017; Guo et al. 2018; Liu et al. 2021; Zhang et al. 2022). Interestingly, the affinity of the interaction between RGA5 HMA and AVR-Pia is similar to the affinities observed for the interaction between RGA5 HMA and AVR-PikD or Pikm-1 HMA and AVR-PikC/AVR-PikF, which do not to result in resistance in planta. A key similarity between these weaker HMA/effector interactions is the rapid dissociation rate of the effector from the HMA, indicative that the RGA5 HMA alone can facilitate, but not maintain, binding of the effector in vitro. This observation is particularly evident when compared to the binding of Pikm-1 to AVR-PikD in which the dissociation rate is considerably slower (Supplemental Fig. S6).

We observed high binding affinity of AVR-PikD, AVR-PikC, and AVR-PikF for the APB mutant (Fig. 4D). As the effector does not dissociate appreciably from the HMA over the time of the experiment, we were unable to accurately calculate binding using multicycle kinetics (Supplemental Fig. S7). Therefore, to quantify the affinity between the APB and AVR-Pik effectors, we used single-cycle kinetics with a long dissociation phase, which allowed us to calculate a *K*_D_ of 0.31, 2.95, and 16.50 nM for AVR-PikD, AVR-PikC, and AVR-PikF respectively (Fig. 4D and Supplemental Table S1).

The engineered APB mutant can bind AVR-Pik effectors with nanomolar affinity, and this strong binding corresponds with the effector association and cell death response observed for Pikm-1^APB^ in planta. By contrast, AVR-Pia rapidly dissociates from all HMA domains, and this corresponds with weak/no response with Pikm-1^APB^ and Pikm-1^RGA5^ chimeras in planta. Taken together, these data suggest binding affinity to the HMA domain is key to recognition in the Pik system, with high-affinity interfaces being essential for initiating a cell death response.

### The structural basis for interaction between the RGA5-APB HMA mutant and AVR-Pik

The Pikm-1 HMA and RGA5 HMA domains are essential for recognition of MAX effectors in their respective NLRs; however, they have spatially distinct effector-binding interfaces (Ortiz et al. 2017; De la Concepcion et al. 2018; Guo et al. 2018).

**Figure 5.**
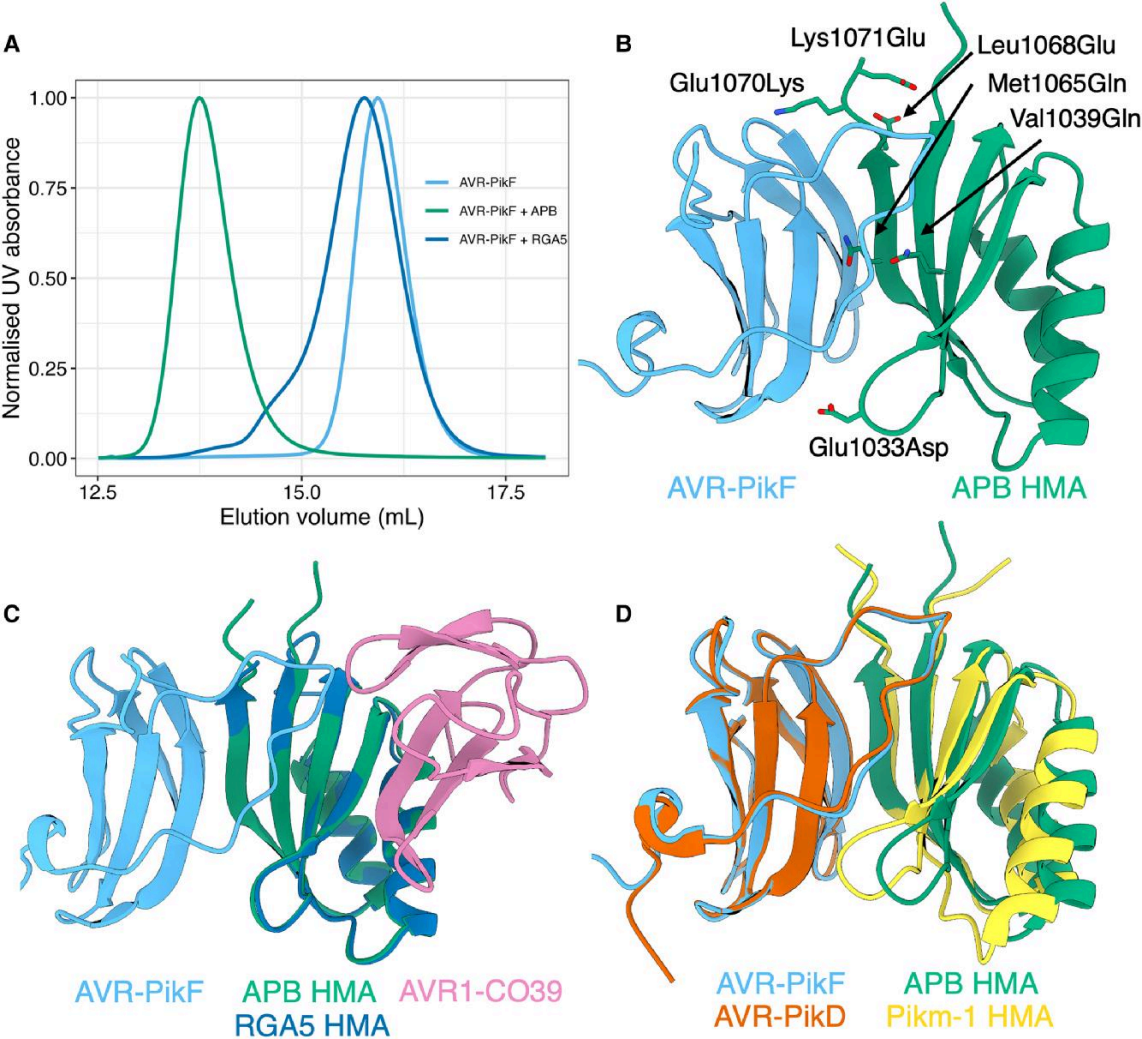
Six mutations in the RGA5 HMA reconstitute a high-affinity APB interface akin to that of the Pik-1 HMA. **A)** Analytical SEC of AVR-PikF with the RGA5 and APB HMA proteins. A mixture of AVR-PikF and APB HMA elutes earlier than a mixture of RGA5 and AVR-PikF or AVR-PikF alone, indicative of complex formation between AVR-PikF and the APB HMA. **B)** The crystal structure of AVR-PikF in complex with the RGA5 APB HMA mutant (PDB: 8B2R). Mutations in RGA5, guided by the structure of the OsHIPP19/AVR-PikF complex, are shown forming contacts with AVR-PikF and are labeled. **C)** Superimposition of the crystal structures of the APB/AVR-PikF complex with the RGA5/AVR1-CO39 complex (PDB ID: 5ZNG) showing the swapped effector-binding interface of the APB HMA compared to the RGA5 HMA. **D)** Superimposition of the APB/AVR-PikF complex with the crystal structure of AVR-PikD bound to the Pikm-1 HMA domain (PDB ID: 6G10), showing the shared effector-binding interface in these complexes.

As the effector recognition interfaces of RGA5 and Pikm-1 HMA domains are different, we determined a crystal structure of the complex between the APB mutant and AVR-PikF, to validate our structural modeling of the RGA5 HMA and confirm we had engineered an AVR-Pik/Pik-HMA-like interface into RGA5 HMA. Using analytical gel filtration, we observed a peak shift after incubating purified AVR-PikF and APB proteins, indicative of stable complex formation (Fig. 5A). Following this, we used a coexpression approach to purify an APB/AVR-PikF complex, which was used to obtain crystals via sparse matrix screening (Supplemental Fig. S8). X-ray diffraction data were collected at the Diamond Light Source, Oxford, resulting in a 1.3-Å data set (Supplemental Table S2) (see details of crystallization and structure solution in Materials and methods). The APB/AVR-PikF complex shares the same interface as Pikm-1 HMA/AVR-PikD and OsHIPP19/AVR-PikF complexes. Structural alignment of these complexes results in an RMSD of 0.51 and 0.39 Å, respectively (Figs. 5, B and D, and S9). As predicted, each of the 6 mutations in the RGA5 HMA generated to facilitate APB is located at the effector interface (Figs. 5B and S10). These mutations are sufficient to generate an APB interface in the RGA5 HMA distinct from that observed for AVR-Pia/AVR1-CO39 (Fig. 5C).

### Allelic mismatching can alleviate autoactivity caused by the integration of nanobody domains

**Figure 6.**
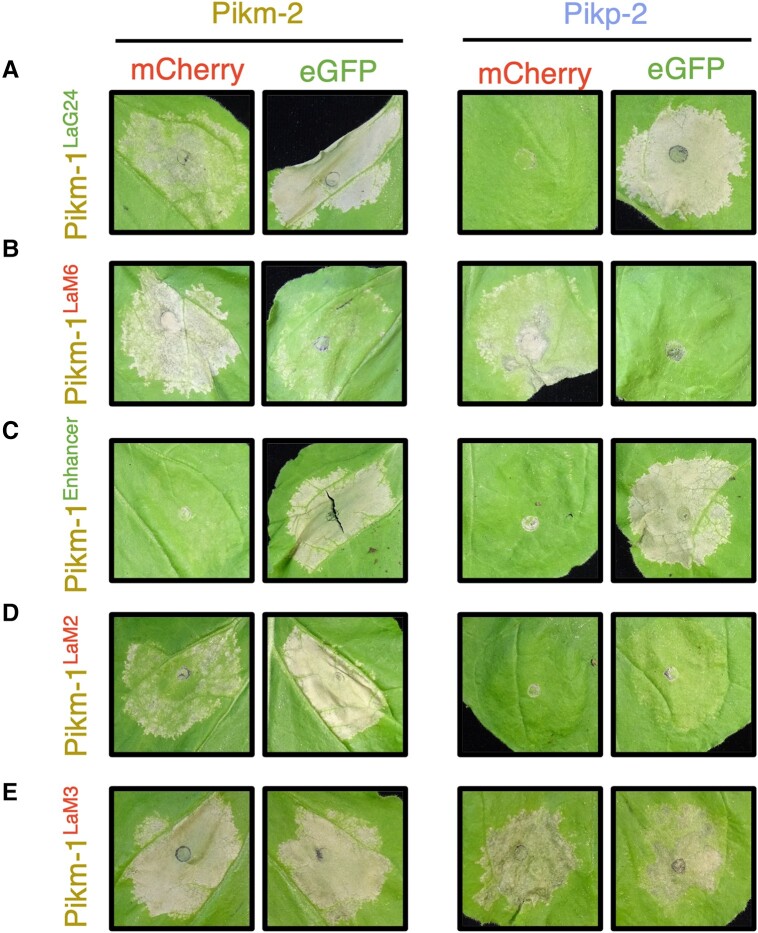
Allelic mismatching of the Pik helper NLRs can be used to alleviate autoactivity from nanobody integration in Pikm-1. Five pikobodies that either respond to the FP eGFP (Pikm-1^LaG24^ and Pik-1^Enhancer^ indicated by green text) or mCherry (Pikm-1^LaM2^, Pikm-1^LaM3^, and Pikm-1^LaM6^, indicated by red text) were tested with the Pikm-2 or Pikp-2 helpers to assess the effect of mismatching on cell death responses in *N. benthamiana*. **A)**, **B)** Pikm-1^LaG24^ and Pikm-1^LaM6^ are autoactive in the presence of Pikm-2; however, coexpression with the Pikp-2 helper does not result in autoactivity. Both pikobodies retain ability to respond to their respective FPs. **C)** Pikm-1^Enhancer^ is not autoactive in the presence of both helpers and can respond to eGFP with equal strength. **D)** Coexpression of Pikm-1^LaM2^ with Pikp-2 alleviates autoactivity observed in the presence of Pikm-2; however, response to mCherry is substantially reduced. **E)** Mismatching of the helper alleles is unable to prevent autoactivity induced by the Pikm-1^LaM3^ pikobody. Details of the NLR mutants used in these experiments can be found in Supplemental Table S3.

Kourelis et al. (2023) demonstrated that “pikobodies,” which are defined as Pik-1–nanobody fusions combined with Pik-2, can confer recognition of either eGFP or mCherry upon integration of VHH nanobodies with affinity for these FPs. However, some of the nanobodies incorporated into Pikm-1 resulted in constitutive cell death when paired with Pikm-2 (Kourelis et al. 2023). To test whether allelic mismatching could be used to alleviate autoactivity caused by nanobody integration, we performed cell death assays in *N. benthamiana* using 5 different nanobody integrations, 2 of which respond to eGFP (Pikm-1^LaG24^ and Pikm-1^Enhancer^) and 3 that respond to mCherry (Pikm-1^LaM2^, Pikm-1^LaM3^, and Pikm-1^LaM6^) (Figs. 6 and S11 and Supplemental Appendix S1J) (Rothbauer et al. 2006; Fridy et al. 2014; Kourelis et al. 2023). The Pikm-1–nanobody fusion was coexpressed with Pikm-2 or Pikp-2 in the presence of eGFP or mCherry and assessed for cell death 5 dpi. Of the 5 nanobody integrations, 4 were demonstrated to be autoactive in the presence of Pikm-2 (Pikm-1^LaG24^, Pikm-1^LaM2^, Pikm-1^LaM3^, and Pikm-1^LaM6^). While Pikm-1^LaM3^ remained autoactive, the autoactivity of Pikm-1^LaG24^, Pikm-1^LaM2^, and Pikm-1^LaM6^ could be mitigated by coexpression with Pikp-2, while still retaining recognition of their cognate FP, although in the case of Pikm-1^LaM2^, cell death signaling was significantly reduced (Figs. 6 and S11 and Supplemental Appendix S1J).

## Discussion

Constitutive immune activation by the combination of incompatible NLRs through breeding or genetic engineering (hybrid necrosis) presents a bottleneck in plant breeding and evolution (Chae et al. 2014; Tran et al. 2017; Calvo-Baltanás et al. 2021; Ordon et al. 2021). Likewise, autoactivity due to engineering presents a bottleneck to strategies for NLR-mediated pathogen resistance (Maidment et al. 2021; Kourelis et al. 2023; Tamborski et al. 2023). The work presented here highlights the importance of factors outside of enhancing effector binding, such as considering the context of NLRs that act in pairs or networks, for the generation of new recognition specificities and NLR combinations without penalties imposed by constitutive immune activation.

### The helper allele Pikp-2 can accommodate for changes in the integrated domain of Pik-1 without triggering effector-independent cell death

We previously reported on the incompatibility of the Pikp and Pikm alleles (De la Concepcion, Vega Benjumea, et al. 2021), highlighting the functional diversification of the Pik receptor pair and linking the specialization of the Pik-2 receptor for its cognate sensor to an Asp230Glu polymorphism in the NB domain. How a single amino acid polymorphism with similar chemical properties triggers such a strong phenotype remains obscure. We previously speculated that the extra carbon atom in the side chain of the Glu230 compared with Asp would be enough to create a steric clash or change in conformation that mimics the active state (De la Concepcion, Maidment, et al. 2021; De la Concepcion, Vega Benjumea, et al. 2021); however, high-resolution structures of the Pik-2 activated complex would be required to test this hypothesis. In this study, we further demonstrated the role of the integrated HMA domain in compatibility of the Pik-1 sensor with the Pik-2 helper. When we introduced the HMA domain from Pikp-1 into Pikm-1, we observed autoactivity with Pikm-2 but not Pikp-2. Pikp-2^D230E^ and Pikm-2^E230D^ mutants, which flip the specialization of each helper, swapped the compatibility of Pik-2 for Pik-1 mutants/chimeras.

Studies involving the swap of the Pikp-integrated HMA for a noncoevolved ancestral version (Białas et al. 2021) or the equivalent HMA domain of OsHIPP19 (Maidment et al. 2022) also showed that this caused autoimmunity, which was removed by mutation of the HMA outside of the effector-binding interface, further supporting a mechanism for coadaptation of the integrated HMA domain with other domains in the sensor Pik-1 and the helper Pik-2. This coadaptation may have led to different sensitivity thresholds of the helpers for the sensors, resulting in differences in the requirements for activation between Pikp-2 and Pikm-2. As such, we observe Pikp-2 to be more permissive of changes in the Pik-1 compared to Pikm-2.

Specific Pik pair combinations are more tolerant to changes in the integrated domain, facilitating engineering of expanded recognition that would otherwise result in constitutive cell death. By considering the context of the engineered receptor domain within the NLR pair, we present an alternative approach to circumventing autoactive immune responses that can limit the potential of NLR engineering for novel disease resistance.

### Allelic mismatching provides avenues for engineering disease resistance

The mismatching strategy reported here opens exciting avenues for the incorporation of new effector recognition motifs into the Pik system, and perhaps other paired NLR systems. Combining the Pikm-1 sensor with the Pikp-2 helper yielded a compatible receptor pair with a greater ability to accept HMA modifications than the natural pairing of the Pikm-1/Pikm-2 alleles. Mismatching of the Pik sensor/helper alleles allowed incorporation of the RGA5 HMA into the Pikm-1 backbone, without autoactivity. Notably, this strategy appears to be useful in areas such as the incorporation of VHH–nanobody fusions into Pikm-1 to allow for tailor-made NLRs deemed “pikobodies” (Kourelis et al. 2023), which are often autoactive in the presence of Pikm-2. Indeed, we demonstrate several Pikm-1–VHH-nanobody chimeras triggered constitutive cell death responses, indicative of autoactivity, and this autoactivity can be mitigated through coexpression of the Pikp-2 helper. Mismatching of the Pik alleles in conjunction with nanobody integration is a powerful combination that could allow for a greater number of successful integrations, streamlining this engineering strategy.

Recently, there have been several reports of engineering expanded effector recognition in integrated HMA domain containing NLRs (De la Concepcion et al. 2019; Liu et al. 2021; Cesari et al. 2022; Maidment et al. 2022; Zhang et al. 2022). In the RGA5/RGA4 system, recognition of AVR-Pib and AVR-PikD has been engineered into the RGA5 HMA domain; however, this results in compromised AVR-Pia recognition (Liu et al. 2021; Cesari et al. 2022; Zhang et al. 2022). Furthermore, implementation of model-driven engineering of RGA5 into crop systems is challenging and has had variable success, with RGA5 mutants that exhibit expanded recognition in a *N. benthamiana* model not always translating to disease resistance in transgenic rice lines (Cesari et al. 2022; Zhang et al. 2022). In parallel, engineering of the Pikp-1 HMA to respond to previously unrecognized AVR-Pik variants has been shown in *N. benthamiana* assays and transgenic rice lines (Maidment et al. 2022).

Interestingly, full replacement of the integrated HMA for the HMA domain of OsHIPP19 caused autoimmunity, which was removed by mutation of the HMA. However, this is not always possible as the approach benefitted from the knowledge of the NLR-ID/effector complex (De la Concepcion et al. 2019; Białas et al. 2021; Maidment et al. 2022). Whether engineering facilitated by allelic mismatching of the Pik pair can provide resistance in transgenic rice lines is yet to be tested and is an important next step to demonstrate the use of this approach for translating recognition in plant models to resistance in crops.

### The Pik system relies on high-affinity effector binding to activate defense responses in planta

We demonstrate the RGA5 HMA domain can be integrated into the Pikm-1 backbone and engineered to recognize AVR-Pik, including variants not recognized by wild-type Pikm-1. As shown by our biophysical and structural analysis, the 6 mutations introduced in the APB mutant of RGA5 HMA domain, based on the host target OsHIPP19, recapitulate a functional APB recognition interface. These data highlight the power of host target-guided design of NLR-ID baits for engineering recognition.

While low levels of cell death were observed, neither Pikm-1^RGA5^ nor Pikm-1^APB^ responded to AVR-Pia at a level comparable with RGA5/RGA4. Indeed, the cell death observed is better compared to the cell death caused by the Pikp pair when coexpressed with AVR-Pia, which has been described as cross-reactivity in *N. benthamiana*, as Pikp is unable to provide full resistance to *M. oryzae* strains carrying AVR-Pia (Varden et al. 2019). It is possible the AVR-Pia/AVR1-CO39 interface is occluded in the Pikm-1^RGA5^ chimera, and co-IP with the APB mutant did not show an association in planta. However, we speculate that the lack of AVR-Pia recognition *N. benthamiana* is likely due to a naturally lower affinity of the effector for the HMA domain as opposed to occlusion of the interaction interface, as we were able to observe some weak cell death when Pikm-1^RGA5^ or Pikm-1^APB^ coexpressed with Pikp-2 was challenged with AVR-Pia. Indeed, substitution of VHH nanobodies that share no homology to the effector recognition interfaces of HMA domains that are able to act as sensory domains for the Pikm-1 sensor makes the hypothesis that reduced response to AVR-Pia by the Pikm-1^RGA5^ and Pikm-1^APB^ receptor is due to an occluded RGA5/AVR-Pia interface less likely. In support of this, previous studies have benchmarked the affinity of AVR-Pia/AVR1-CO39 for the RGA5 HMA domain in the micromolar range (Ortiz et al. 2017; Guo et al. 2018), while AVR-Pik effectors bind their cognate integrated HMA domains with nanomolar affinity (for interactions that result in cell death responses). These previous observations were corroborated by the biophysical analyses performed in this study (Supplemental Fig. S6A and Table S1), which also measure the affinities of RGA5 and the APB HMA domains for AVR-Pia in the micromolar range, compared to the nanomolar affinities of the APB HMA and Pikm-1 HMA domains for AVR-Pik effectors.

It remains unclear why the binding affinities of the Pik HMA and RGA5 HMA domains for their cognate effectors differ so significantly. However, the RGA5 post-LRR region, which contains the HMA domains, as characterized by AlphaFold2 (Colab Fold v 1.5.2 [Jumper et al. 2021; Mirdita et al. 2022; Supplemental Fig. S12]), which may contribute to effector binding. Indeed, AVR-Pia is known to associate with regions of the RGA5 receptor outside of the HMA domain (Ortiz et al. 2017). However, RGA5 receptors where the HMA domain has been deleted are unable to respond to AVR-Pia in cell death assays (Cesari et al. 2013). Furthermore, recent studies engineering AVR-Pib and AVR-Pik recognition in RGA5, respectively, showed mutations outside of the HMA-influenced effector recognition in planta (Liu et al. 2021; Zhang et al. 2022). Collectively, available data suggest that RGA5-mediated effector recognition requires the HMA domain, but alone it is not sufficient for effector recognition and works together with other regions at the RGA5 C-terminus. Inclusion of these additional regions from RGA5 into the Pik receptor backbone alongside the RGA5 HMA domain could support AVR-Pia recognition but, equally so, may affect Pik sensor/helper compatibility. Certainly, the additional complexity of the RGA4/RGA5 system makes engineering this receptor pair more challenging compared to the Pik NLRs.

Modification of plant NLRs has proven challenging due to the lack of understanding of the context of NLRs as part of complex systems. In this study, we facilitate NLR-mediated resistance engineering by exploiting the allelic diversity in the Pik NLR pair to allow for generation of receptors with expanded recognition specificities that would otherwise result in constitutive cell death. Our structural, biophysical, and in planta analyses demonstrate the Pik system requires a high-affinity effector-binding interface to allow for binding to translate to defense, and as a single domain, the RGA5 HMA domain appears to lack the affinity for AVR-Pia to facilitate a robust Pik chassis-mediated cell death response. However, our engineering of RGA5 HMA to recognize AVR-Pik from within the Pikm-1 chassis highlights the strengths of this system for engineering; only a single high-affinity interface needs to be present to mediate effector recognition, making the Pik system a simple but efficient means for generating bespoke NLR resistance. This work lays the foundation for the incorporation of new effector recognition motifs into the Pik system and is a key advance toward the development of designer NLRs that can be tailored to specific secreted pathogen signatures.

## Materials and methods

### Plant materials and growth conditions

*N. benthamiana* plants were grown in a controlled environment room at 22 °C constant temperature and 80% relative humidity, with a 16-h photoperiod with lighting provided by a combination of 2 Philips Master TL-D 58W/840 and Sylvania GRO-LUX F58W/GRO-T8 fluorescent tubes.

### Gene cloning—In planta expression

For expression in planta, full-length *Pikp-1* and *Pikm-1*, and relevant mutants, were cloned with a 6xHIS/3xFLAG tag into the pICH47742 plasmid, full-length *Pikp-2* and *Pikm-2* were cloned by GoldenGate cloning via *Bsa1* into pICH47751 with a C-terminal 6xHA tag, and *Pikp-2*^D230E^ and *Pikm-2*^E230D^ mutants were generated by site-directed mutagenesis as previously described (De la Concepcion et al. 2018; De la Concepcion, Vega Benjumea, et al. 2021).

To generate the Pikm-1 DOM2 acceptor, the *Pikm-1* sequence was domesticated of *BsaI* and *BbsI* restriction sites to allow compatibility with our Golden Gate cloning system and cloned into the Level 0 CDS(ns) pICSL01005 acceptor. Once domesticated, the position of the *Pikm-1* HMA domain was substituted with predomesticated iGEM amilCP negative selection reporter cassettes internally flanked by outward pointing *Esp3I* sites to produce CAGA (5′) and GATG (3′) cloning overhangs. *Esp3I* was used to incorporate an iGEM RFP-negative selection reporter cassette, internally flanked by outward pointing *BbsI* sites presenting CAGA (5′) and GATG (3′) cloning overhangs, allowing cloning of new domains via *BbsI* into the Pikm-1 DOM2 acceptor in the analogous position to where the HMA domain was located.

The *RGA5* HMA and APB mutants were cloned into the Pikm-1 DOM2 acceptor via Golden Gate cloning with *BbsI* to assemble a full-length *Pikm-1* receptor chimera. Full-length *Pikm-1*^RGA5^ and *Pikm-1*^APB^ were subsequently cloned into pICH47742 via *BsaI*, with a C-terminal 6xHIS/3xFLAG tag.

*RGA5* and *RGA4* were assembled into the binary *Agrobacterium* (*Agrobacterium tumefaciens*) expression vector pICSL4723 (Engler et al. 2014) via *BbsI*. *RGA4* was tagged with a C-terminal 6xHA tag, and *RGA5* was left untagged to prevent effects on receptor function. Expression of *RGA4* and *RGA5* was driven by the *Arabidopsis* (*A. thaliana*) actin and 2 × 35S promoters, respectively. For cell death assays, AVR-Pia was cloned untagged into pJK268c with P19 via *Bbs1*, with expression driven by a 2 × 35S promoter. For co-IP assays, an N-terminally 4xMYC tagged AVR-Pia was cloned into pICH47752 via *Bsa1*.

*AVR-Pik* effector variants used in this study were described previously (De la Concepcion et al. 2018). *PWL2* was cloned into pICH47751 under a Ubi10 promoter and 35S terminator and C-terminal 4xMYC tag via Golden Gate cloning.

Pikobody constructs for coexpression of *Pikm-2* with the *Pikm-1*–nanobody fusions, either the LaG24, GFP enhancer, LaM2, LAM3, or LAM6 nanobody, were described in Kourelis et al. (2023). For mismatching, the above constructs were redesigned to coexpress *Pikp-2* rather than *Pikm-2*.

### Gene cloning—Recombinant expression in *Escherichia coli*

The *RGA5*, *APB*, and *Pikm-1* HMA domains and the *AVR-Pik* effector variants and *AVR-Pia* effector were cloned into pOPIN-GG vector pPGN-C iva (Bentham et al. 2021) with a cleavable N-terminal 6xHIS-GB1-3C tag via Golden Gate cloning with *BsaI*. *AVR-Pii* effector domain was cloned with a cleavable N-terminal MBP tag and an uncleavable C-terminal 6xHIS via in-fusion cloning into pOPINE (Berrow et al. 2007). For coexpression with the *APB* HMA for crystallography studies, *AVR-PikF* was cloned into pPGC-K (Bentham et al. 2021) without a tag via Golden Gate cloning with *BsaI*.

### In planta co-IP

Transient gene expression in planta was performed by infiltrating 4-wk-old *N. benthamiana* plants with *A. tumefaciens* strain GV3101 (C58 [rifR] Ti pMP90 [pTiC58DT-DNA] [gentR] nopaline [pSoup-tetR]). *A. tumefaciens* carrying NLRs and effectors were infiltrated at OD_600_ 0.4 and 0.6, respectively, in agroinfiltration medium (10 mM MgCl_2_, 10 mM 2-(N-morpholine)-ethanesulfonic acid [MES], and pH 5.6) with the addition of 150 *µ*M acetosyringone.

Leaf tissue was collected 3 d postinfiltrations (dpi) and frozen in liquid nitrogen before processing. Samples were ground to a fine powder in liquid nitrogen using a mortar and pestle before being mixed with 2 times weight/volume ice-cold Co-IP extraction buffer (25 mM Tris pH 7.5, 150 mM NaCl, 1 mM EDTA, 10% v/v glycerol, 2% w/v PVPP, 10 mM DTT, 1 × complete protease inhibitor tablet per 50 mL [Roche], and 0.1% Tween 20). Samples were centrifuged at 4,200 × *g* at 4 °C for 20 min, and supernatant was passed through a 0.45-*µ*m Ministart syringe filter. SDS–PAGE/immunoblot analysis was used to identify proteins in the sample with the use of anti-FLAG M2 antibody (Sigma) and anti-MYC antibody (Santa Cruz Biotechnology) for NLRs and effectors, respectively.

For immunoprecipitation, 2 mL of filtered plant extract was incubated with 30 *µ*L of M2 anti-FLAG magnetic beads (Sigma) in a rotary mixer for 3 h at 4 °C. The FLAG beads were separated from the supernatant with the use of a magnetic rack to allow for the removal of the supernatant. The beads were then washed with 1 mL of IP buffer (25 mM Tris pH 7.5, 150 mM NaCl, 1 mM EDTA, 10% glycerol, and 0.1% Tween 20). The FLAG beads were washed 3 times using this method. After washing, 30 *µ*L of LDS RunBlue sample buffer was added to the FLAG beads and incubated for 10 min at 70 °C. The beads were then applied to a magnetic rack and the supernatant was loaded to SDS–PAGE gels and subsequently used for immunoblot blot analysis. PVDF membranes were probed with anti-FLAG M2 and anti-MYC antibodies to detect NLRs and effectors, respectively.

### *N. benthamiana* cell death assays and cell death scoring

Cell death assays and scoring were performed as described previously (De la Concepcion, Maidment, et al. 2021; De la Concepcion, Vega Benjumea, et al. 2021). In brief, *N. benthamiana* tissue was infiltrated with *A. tumefaciens* GV3101 (C58 [rifR] Ti pMP90 [pTiC58DT-DNA] [gentR] nopaline [pSoup-tetR]) carrying NLRs and effectors at OD_600_ 0.4 and 0.6, respectively, and P19 at OD_600_ 0.1. Leaves were imaged 5 dpi from the abaxial side for UV fluorescence images. Photos were taken using a Nikon D4 camera with a 60-mm macrolens, ISO set 1600 and exposure ∼10 s at F14. The filter was a Kodak Wratten No.8 and the white balance was set to 6,250 °K. Blak–Ray longwave (365 nm) B-100AP spotlight lamps were moved around the subject during the exposure to give an even illumination. Images shown are representative of 3 independent experiments with a minimum internal technical repeat; a minimum of 45 data points across 3 repeats were collected per sample across 30 plants. The cell death scoring was performed using the cell death index previously presented in Maqbool et al. (2015). Dot plots were generated using R 4.0.5 (https://www.r-project.org) with the package ggplot2 (Wickham 2016). The size of the center dot at each cell death value is directly proportional to the number of replicates in the sample with that score. All individual data points are represented as dots. Statistical analysis was performed using estimation graphics (Ho et al. 2019) with the besthr R package (MacLean 2019; De la Concepcion, Vega Benjumea, et al. 2021) and can be found in Supplemental Appendix S1.

### Protein expression and purification from *E. coli*

Expression vectors containing the 6xHIS-GB1-tagged effectors and HMA domains were transformed into *E. coli* SHuffle cells. Using an overnight culture for inoculum, 8 L of SHuffle cells were grown in autoinduction media (AIM) at 30 °C to an OD_600_ of 0.6 to 0.8 before the temperature was reduced to 18 °C for overnight induction (Studier 2005). Cells were pelleted by centrifugation at 5,000 × *g* for 10 min and resuspended in lysis buffer (50 mM HEPES pH 8.0, 500 mM NaCl, 30 mM imidazole, 50 mM glycine, and 5% v/v glycerol). Cell lysate was clarified by centrifugation at 45,000 × *g* for 20 min following disruption of the resuspended pellet by sonication. Proteins were purified from clarified lysate via Ni^2+^ immobilized metal chromatography (IMAC) coupled with size-exclusion chromatography (SEC). The 6xHIS-GB1 tag was removed via overnight cleavage with 3C protease at 4 °C before a final round of SEC using a buffer of 10 mM HEPES pH 8 and 150 mM NaCl. Proteins were flash frozen in liquid nitrogen before storage at −80 °C.

For coexpression of the APB/AVR-PikF complex, *E. coli* SHuffle cells were cotransformed with 6xHIS-GB1-tagged APB HMA and untagged AVR-PikF and plated on dual resistance carbenicillin and kanamycin selection. Expression and purification of the complex were then performed as described above, using dual selection for growth in large-scale cultures.

### Crystallization, X-ray data collection, structure solution, and refinement

The APB/AVR-PikF complex was concentrated to 10 mg/mL in SEC buffer (10 mM HEPES pH 8.0 and 150 mM NaCl) for crystallization. Sitting drop and vapor diffusion crystallization trials were set up in 96-well plates using an Oryx Nano robot (Douglas Instruments). Crystallization plates were incubated at 20 °C. APB/AVR-PikF crystals appeared in the SG1 Screen (Molecular Dimensions) after 10 d in a 0.1 M BIS-TRIS pH 5.5, 25 w/v % PEG 3350 condition. Crystals were harvested and snap frozen in liquid nitrogen prior to shipping.

Crystals of the APB/AVR-PikF complex diffracted to 1.3 Å and X-ray data sets were collected at the Diamond Light Source on the i04 beamline under proposal m × 25108. The data were processed using the xia2 pipeline and AIMLESS as implemented in CCP4i2 (Winn et al. 2011). Using the structure of the OsHIPP19/AVR-PikF complex (PDB ID: 7B1I) as a template, the structure of the APB/AVR-PikF complex was solved using molecular replacement with PHASER (McCoy et al. 2007). The final structure was obtained after iterative cycles of refinement using COOT and REFMAC (Murshudov et al. 1997; Emsley and Cowtan 2004). Structure geometry was validated using the tools in COOT and MOLPROBITY (Emsley and Cowtan 2004; Chen et al. 2010). Protein interface analyses were performed using QtPISA and ChimeraX (Krissinel and Henrick 2007; Pettersen et al. 2021). Models are visualized using ChimeraX (Pettersen et al. 2021). X-ray diffraction data can be found in the Protein Data Bank (https://www.ebi.ac.uk/pdbe/) under the accession number 8B2R.

### Analytical SEC

One hundred fifty micrograms of purified AVR-PikF was mixed with 150 *µ*g of the RGA5 and APB HMA domains and incubated on ice for 30 min before separation via SEC using a Superdex S75 10/300 GL size-exclusion column (Cytiva). As a negative control, 150 *µ*g of AVR-PikF was run alone. HMA domains were not run separate from AVR-PikF due to low or no absorbance at A_280_ resulting in no observable peak in the chromatogram. Chromatograms were visualized using the ggplot2 R library in R 4.0.5 (Wickham 2016).

### Biophysical analysis with SPR

SPR was performed using a Biacore 8K (Cytiva). Purified HMA domains were immobilized on a Series S Sensor CM5 Chip (Cytiva) via amine-coupling using 0.4 M 1-ethyl-3-(3-dimethylaminopropyl)-carbodiimide (EDC) and 0.1 M N-hydroxysuccinimide (NHS) to activate the chip surface prior to binding of HMAs at 2 concentrations on different channels, a high concentration (30 nM, ∼2,000 response units [RUs]) and a low concentration (0.3 nM, ∼200 RUs) to allow for accurate measurement of affinity and kinetics of strong and weak interactors; 1 M ethanolamine–HCl pH 8.5 was then used to block the CM5 chip after coupling was completed.

Samples were run in HBS-EP+ running buffer (0.1 M HEPES, 1.5 M NaCl, 0.03 M EDTA, and 0.5% v/v Tween 20), and the chip was regenerated after each cycle with an ionic regeneration buffer (0.46 M KSCN, 1.83 M MgCl_2_, 0.92 M urea, and 1.83 M guanidine–HCl). Effectors were run over the chip at a flow rate of 100 *µ*L/min; contact and dissociation time varied depending on the experiment (see below).

Where possible, we performed multicycle kinetics to assess the affinity and binding kinetics of the effectors for the HMA. For strong interactions (Pikm-1 HMA with AVR-PikD), we used serial dilutions of effectors from 50 to 0 nM, and for weak interactions, we used serial dilutions of 50 to 0 *µ*M (AVR-Pia with RGA5 HMA, APB HMA, and Pikm-1 HMA; AVR-PikC with RGA5 HMA and Pikm-1 HMA; and AVR-PikF with RGA5 HMA and Pikm-1 HMA), with each concentration being performed in triplicate. Contact time and dissociation times for the experiment were set at 120 s.

For strong interactions, we performed single-cycle kinetics due to the extremely slow dissociation rates of the effectors from the HMA domains, which interfered with accurate calculations of kinetic parameters and binding affinity. For single-cycle kinetics, increasing concentrations of effector (0 to 50 nM) were sequentially flowed over the HMA-bound sensor chip each with a contact time of 120 s before a single dissociation phase of 600 s. Each cycle was performed in triplicate.

SPR sensograms were analyzed with the Biacore Insight Evaluation Software (Cytiva) and equilibrium dissociation constants (*K*_D_) values were calculated using a 1:1 binding model from a kinetic fit model. Residual graphs are generated from the subtraction of the experimental data from the fit model (ΔFit–Exp). Sensograms and residual graphs were generated in R 4.0.5 using the ggplot2 R package (Wickham 2016).

### Accession numbers

Pikp-1 E9KPB5, Pikm-1 B5UBC1, Pikp-2, and Pikm-2 (D5L9H7), RGA5 (F7J0N2), RGA4 (F7J0M4), AVR-Pik (variants D, C, and F) (C4B8C2), AVR-Pia (B9WZW9), AVR-Pii (L7J571), and PWL2 (A0A3G2LZD8).

## Supplementary Material

koad204_Supplementary_Data

## Data Availability

The PDB accession number for structural data is provided in the manuscript.

## Supplemental data

The following materials are available in the online version of this article.

**Supplemental Figure S1.** Cell death scoring of Pik-1 chimeras coexpressed with Pikp-2 and Pikm-2 in *N. benthamiana*.

**Supplemental Figure S2.** The effect of point mutations in the *β*1 strand, α2 helix, and *β*4 strand secondary structures of the Pikm-1 HMA domain and their effect on compatibility with the Pikm-2 helper in *N. benthamiana*.

**Supplemental Figure S3.** Cell death scoring of point mutations in the α2 helix, *β*1, and *β*4 strands of the Pikm-1 HMA domain when expressed with the Pikm-2 helper in *N. benthamiana*.

**Supplemental Figure S4.** The Pikm-1^RGA5^ chimera does not respond to AVR-PikD in *N. benthamiana*.

**Supplemental Figure S5.** Structure-guided engineering of RGA5 using OsHIPP19 as a template to generate the APB mutant.

**Supplemental Figure S6.** Multicycle kinetics SPR sensograms capturing effector/HMA interactions.

**Supplemental Figure S7.** Multicycle kinetics SPR sensograms of the APB mutant with AVR-Pik variants.

**Supplemental Figure S8.** Crystallization of the APB/AVR-PikF HMA complex.

**Supplemental Figure S9.** Superimposition of the crystal structure of APB HMA/AVR-PikF complex with other MAX effector/HMA complexes.

**Supplemental Figure S10.** Superimposition of the crystal structure of APB HMA/AVR-PikF complex with the morph model generated from an OsHIPP19 template.

**Supplemental Figure S11.** Cell death scoring of pikobodies coexpressed with Pikp-2 and Pikm-2 in *N. benthamiana*.

**Supplemental Figure S12.** AlphaFold2 prediction of the C-terminal domains of RGA5 describes previously uncharacterized domains.

**Supplemental Table S1.** Full kinetic parameters for HMA-effector interactions as measured by SPR.

**Supplemental Table S2.** Data collection and refinement statistics.

**Supplemental Table S3.** Mutants used in this study.

**Supplemental Table S4.** Key resources used in this study.

**Supplemental Table S5.** Primers used in this study.
