# Supplementary material for: Allelic compatibility in plant immune receptors facilitates engineering of new effector recognition specificities
Source: Plant Cell. 2023 Jul 24;35(10):3809–27. doi: 10.1093/plcell/koad204 (PMC10533329; doi:10.1093/plcell/koad204)
Supplement: koad204_Supplementary_Data [file koad204_supplementary_data.zip › tpc.23.00251Supplemental Figures and Tables.pdf]

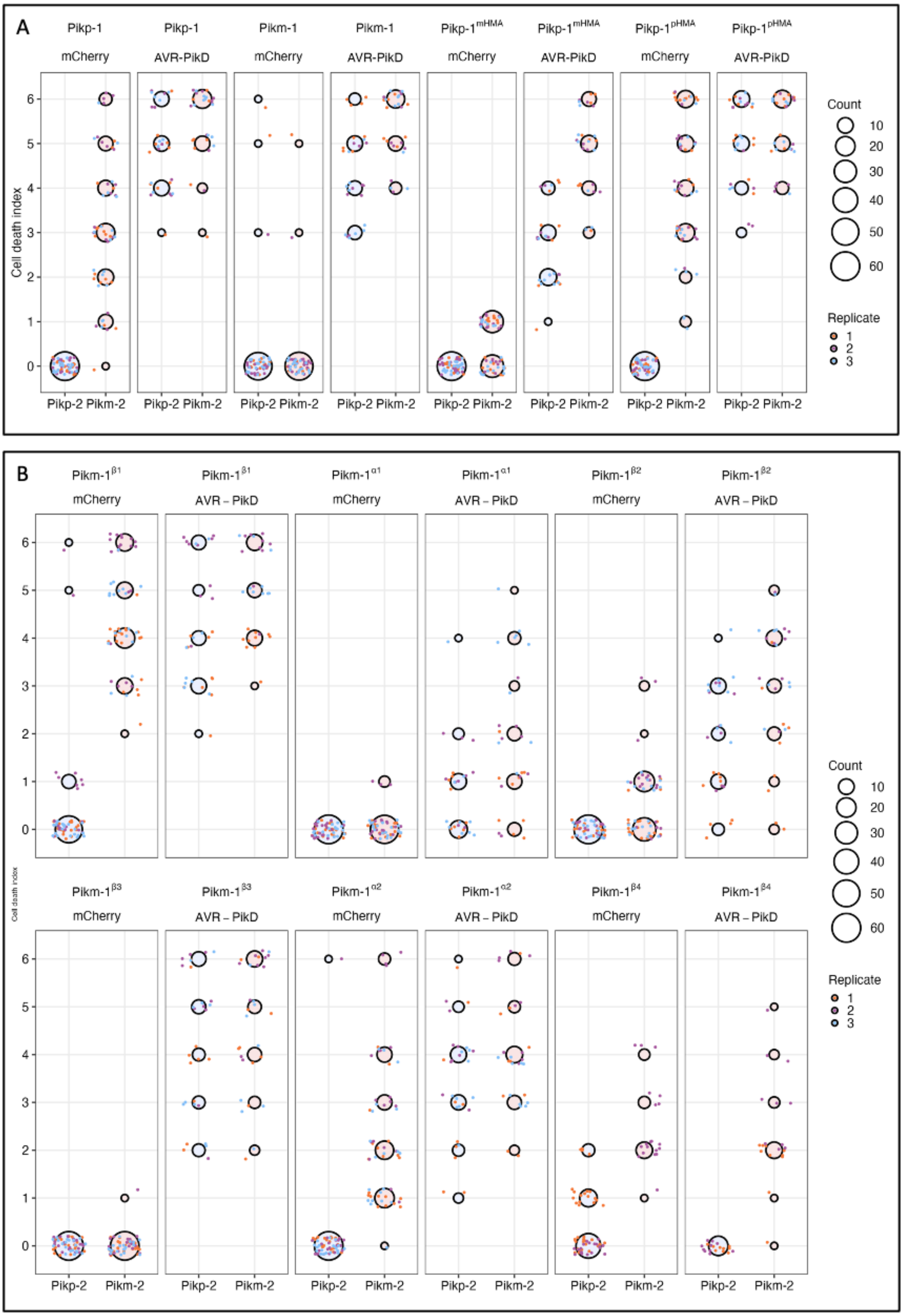

Supplemental Figure S1. Cell death scoring of Pik-1 chimeras co-expressed with Pikp-2 and Pikm-2 in *N. benthamiana*. Supports Figure 2. A) Scoring of Pikp-1^{mHMA} and Pikm-1^{pHMA} chimeras when co-expressed with Pikp-2 or Pikm-2 and either AVR-PikD or mCherry. **B)** Scoring of Pikm-1 chimeras carrying different secondary structures from the Pikp-1 HMA when co-expressed with Pikp-2 or Pikm-2 and either AVR-PikD or mCherry. Scoring is

represented as dot plots. The total number of repeats was 60 per sample for both **A** and **B**. For each sample, all the data points are represented as dots with a distinct colour for each of the three biological replicates; these dots are jittered around the cell death score for visualisation purposes. The size of the central dot at each cell death value is proportional to the number of replicates of the sample with that score. Statistical analysis of these results is shown in **Appendix 1 B (Supplemental File 1)**. Details of the NLR mutants used in these experiments can be found in **Supplemental Table S3**.

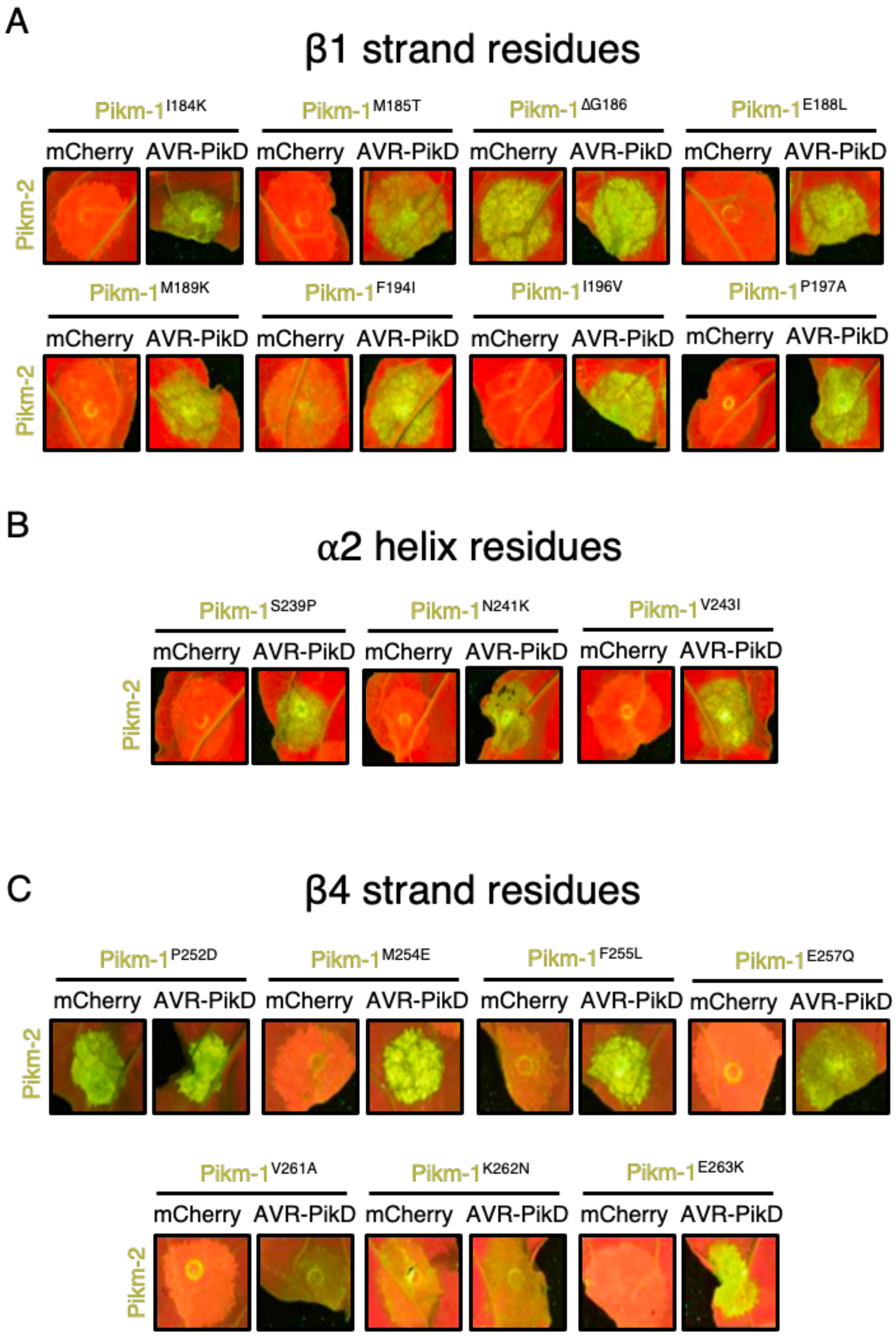

Supplemental Figure S2. The effect of point mutations in the β1 strand, α2 helix, and β4 strand secondary structures of the Pikm-1 HMA domain, and their effect on compatibility with the Pikm-2 helper in *N. benthamiana*. Supports Figure 2. Point mutations are substituted with the corresponding residue in the Pikp HMA. **A)** Individual point mutations of the residues in the β1 strand. **B)** Individual point mutations of the residues in the α2 helix. **C)** Individual point mutations of the residues in the β4 strand. Quantification and statistical analysis of these results are shown in **Supplemental Figure S3, Appendices 1 D, E, F (Supplemental File 1).** Details of the NLR mutants used in these experiments can be found in **Supplemental Table S3**.

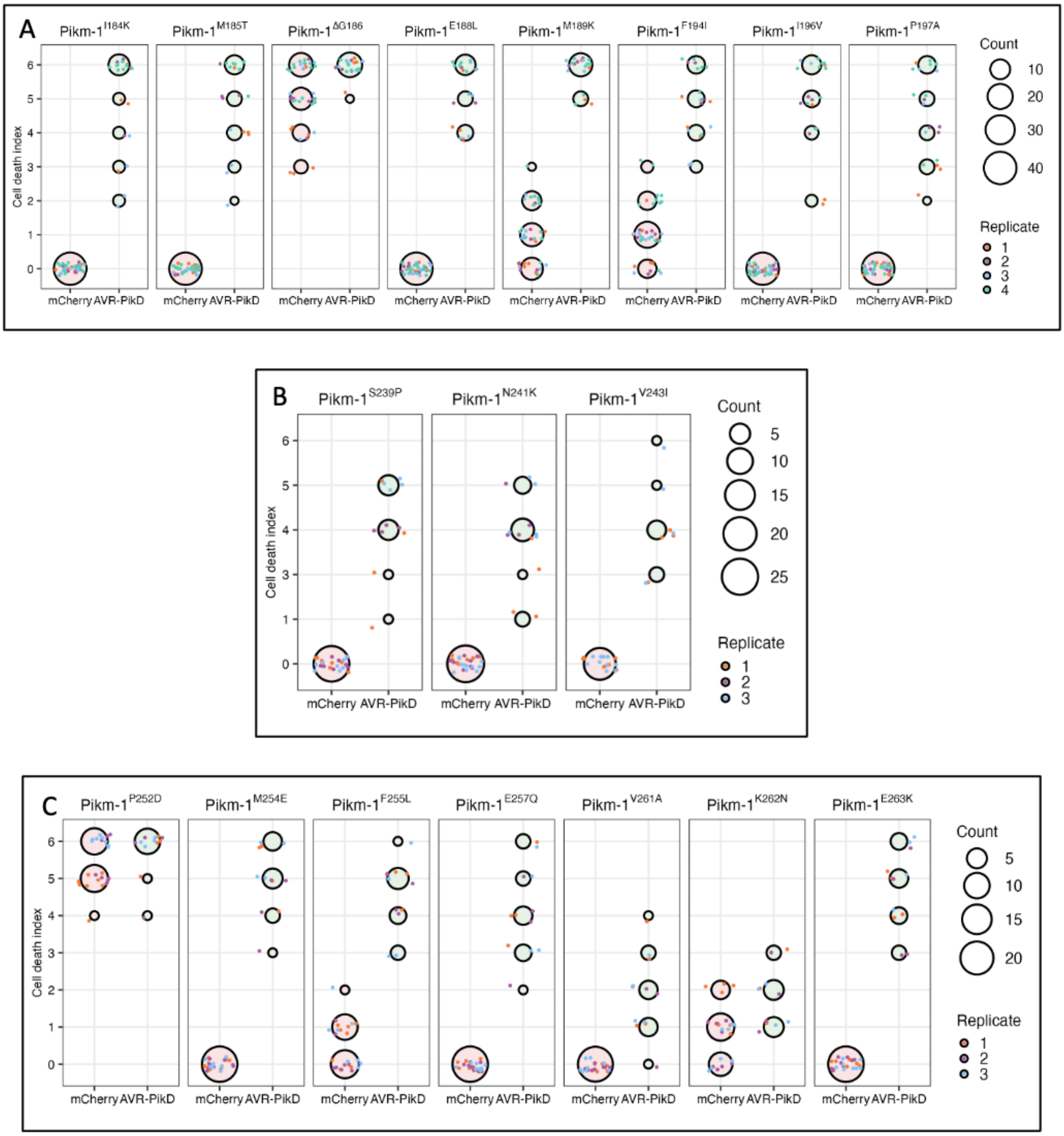

Supplemental Figure S3. Cell death scoring of point mutations in the α2 helix, β1, and β4 strands of the Pikm-1 HMA domain when expressed with the Pikm-2 helper in *N. benthamiana*. Supports Figure 2. A) Residues belonging to the β1 strand. **B)** Residues belonging to the α2 helix. **C)** Residues belonging to the β4 strand. Scoring is represented as dot plots. The total number of repeats was 40 per sample. For each sample, all the data points are represented as dots with a distinct color for each of the three biological replicates; these dots are jittered around the cell death score for visualization purposes. The size of the central dot at each cell death value is proportional to the number of replicates of the sample with that score. Quantification and statistical analysis of these results are shown in **Appendices 1 D, E, F (Supplemental File 1)**. Details of the NLR mutants used in these experiments can be found in **Supplemental Table S3**.

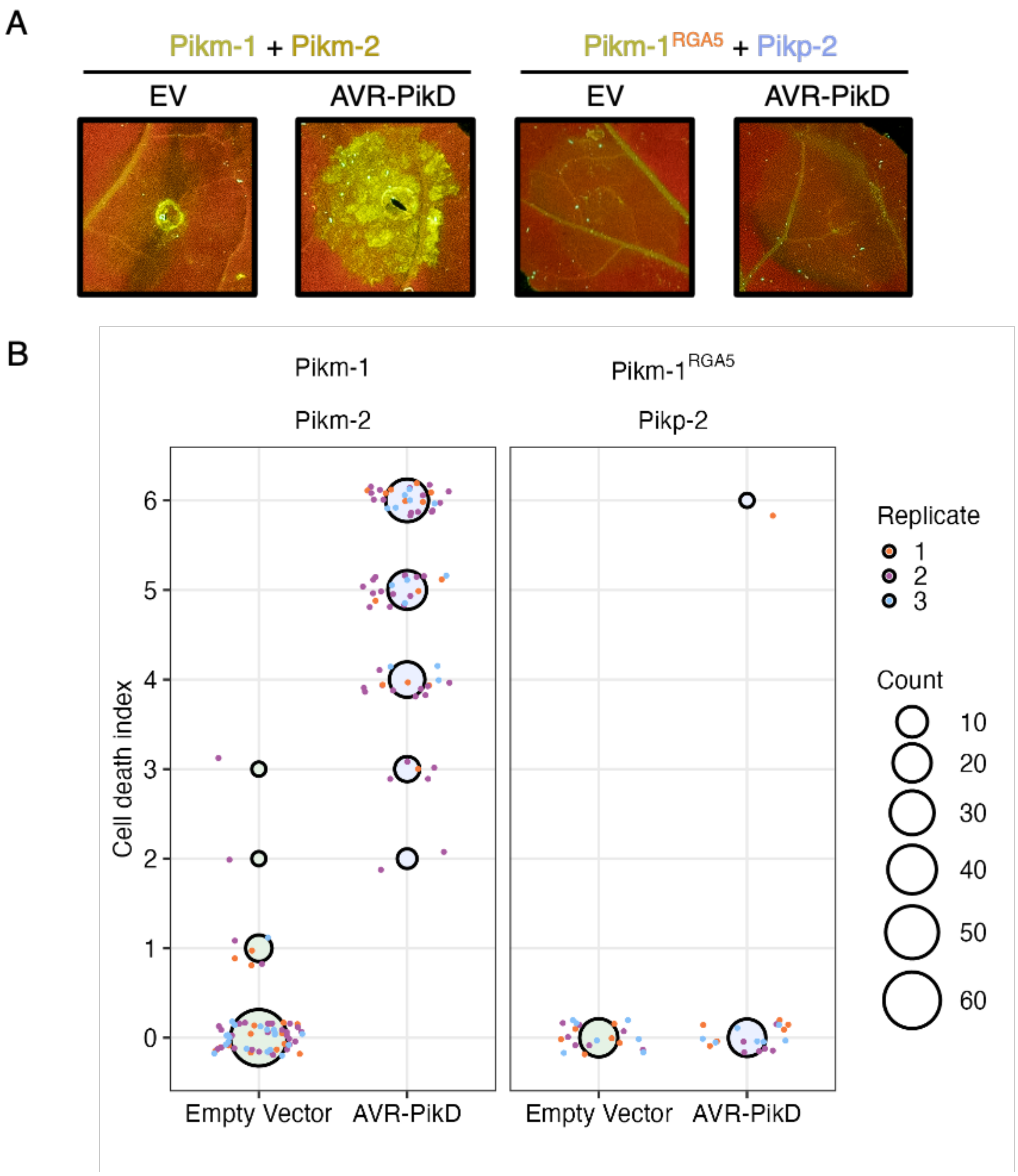

Supplemental Figure S4. The Pikm-1^{RGA5} chimera does not respond to AVR-PikD in *N. benthamiana*. Supports Figure 3. A) Co-expression of the Pikm-1^{RGA5} chimera with Pikp-2 and AVR-PikD in *N. benthamiana* leaves does not result in cell death. Wildtype Pikm-1 and Pikm-2 co-expressed with AVR-PikD shown as positive control **B)** Cell death scoring of **A)** represented as dot plots. For each sample, all the data points are represented as dots with a distinct color for each of the three biological replicates; these dots are jittered around the cell death score for visualization purposes. The size of the central dot at each cell death value is proportional to the number of replicates of the sample with that score. Statistical analyses of these results are shown in **Appendix 1 H (Supplemental File 1)**. Details of the NLR mutants used in these experiments can be found in **Supplemental Table S3**.

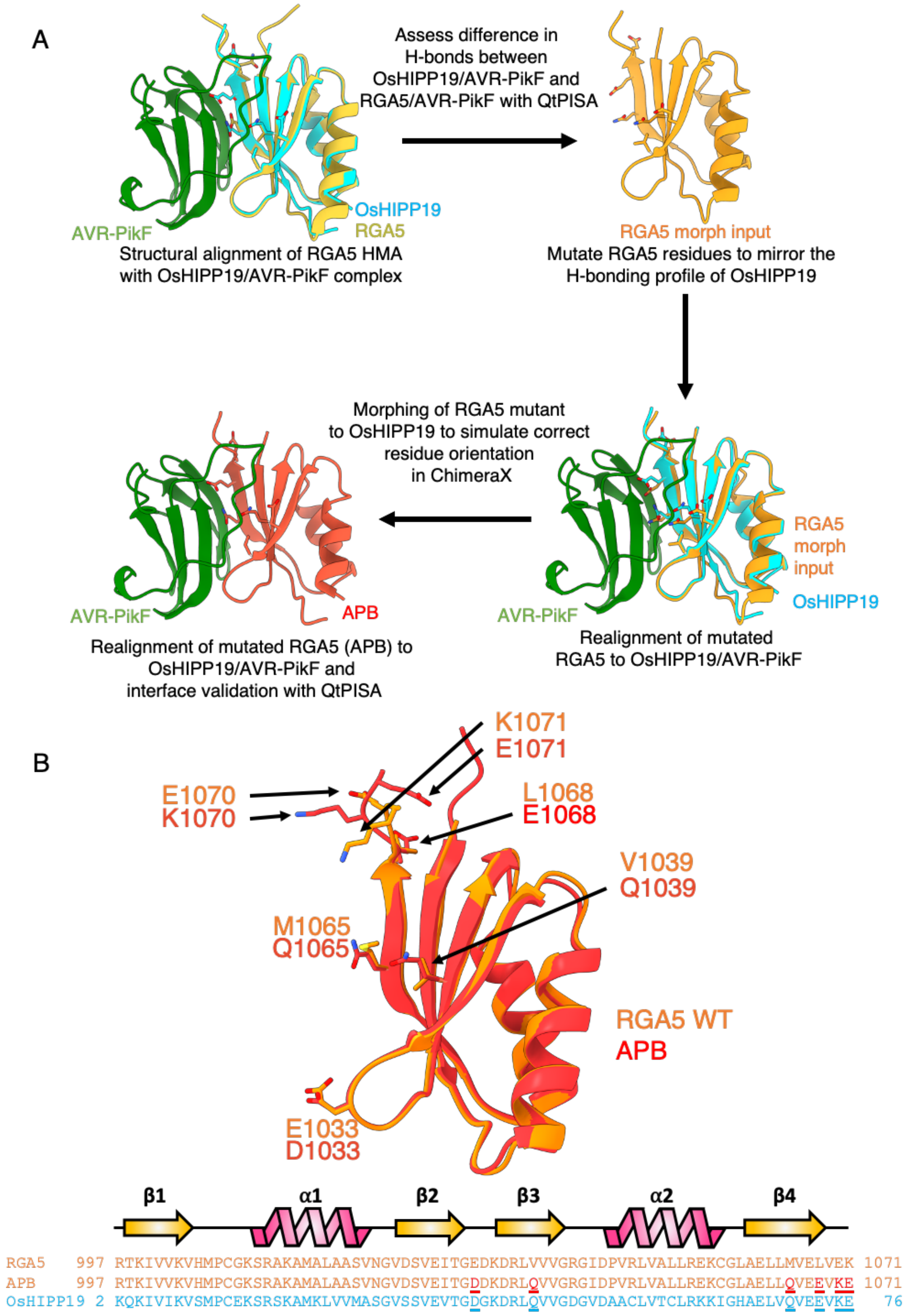

Supplemental Figure S5. Structure-guided engineering of RGA5 using OsHIPP19 as a template to generate the APB mutant. Supports Figure 4. A) Modelling pipeline using QtPISA and ChimeraX to make the RGA5 APB mutant using OsHIPP19 as a template. **B)** Structural alignment of the RGA5 HMA (PDB: 5ZNG) with the APB mutant with a sequence alignment highlighting the changes informed by OsHIPP19.

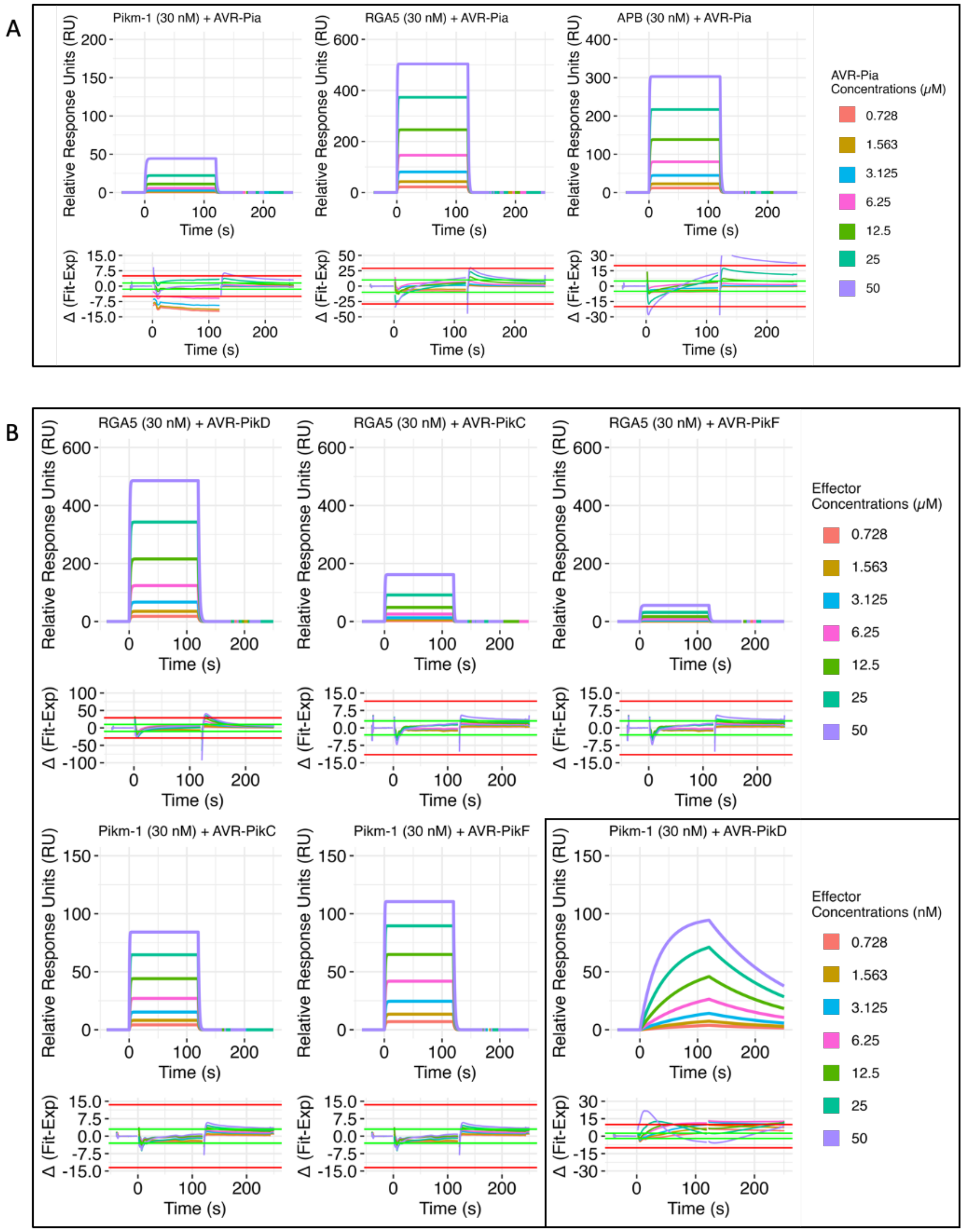

Supplemental Figure S6. Multicycle kinetics SPR sensograms capturing effector/HMA interactions. SupportsFigure 4. Effectors were flowed over the HMA-bound CM5 chip at 8 concentrations (0 – 50 μM), with the exception of the strong Pikm-1/AVR-PikD interaction where 0–50 nM of AVR-PikD was used. Kinetic and binding parameters were calculated using a 1:1 binding model. Residual graphs are shown under the sensograms, with data between the red lines being deemed reliable. **A)** Sensograms of the Pikm-1, RGA5 and APB HMA domains with AVR-Pia. **B)** Sensograms of RGA5 and Pikm-1 HMA domains with AVR-Pik effectors.

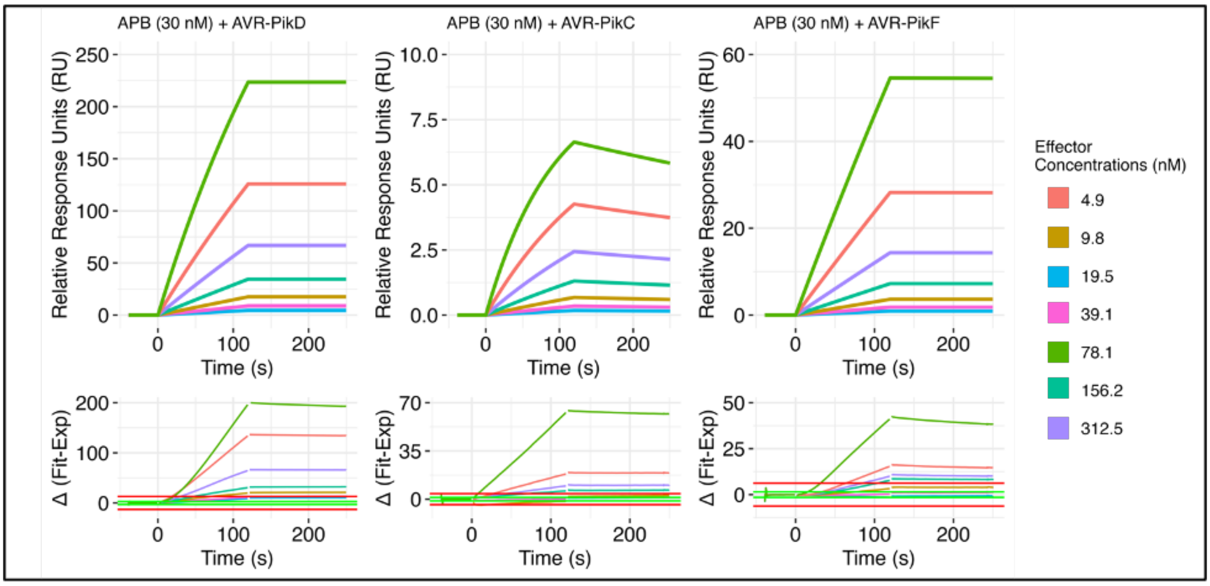

Supplemental Figure S7. Multicycle kinetics SPR sensograms of the APB mutant with AVR-Pik variants. Supports Figure 4. Effectors were flowed over the HMA-bound CM5 chip at 7 concentrations (4.9–312.5 nM). Kinetic and binding parameters were calculated using a 1:1 binding model. Residual graphs are shown under the sensograms, with data between the red lines being deemed reliable. Due to the high affinity of the effectors for the HMA, the effectors failed to dissociate from the APB chip between runs. This is reflected in the poor fit as described by the residuals, and the decreasing relative response as seen between the samples.

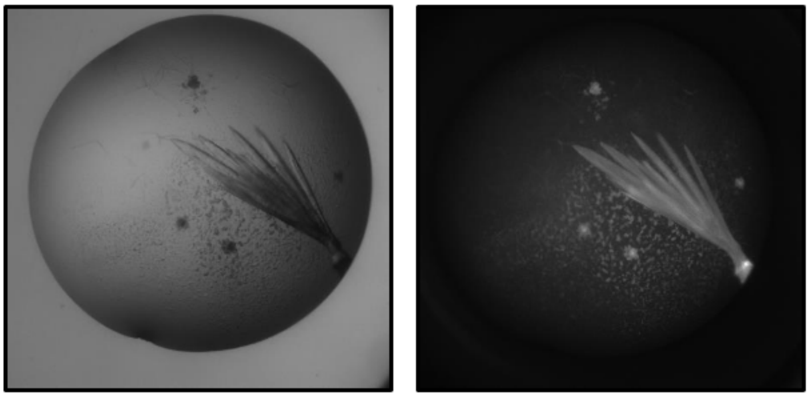

Supplemental Figure S8. Crystallization of the APB/AVR-PikF HMA complex. Supports Figure 5. APB/AVR-PikF HMA complex crystals formed in ShotGun 1 sparse matrix screen (Molecular Dimensions) well B1 (0.1 M BIS-TRIS pH 5.5, 25% PEG3350) after 10 days.

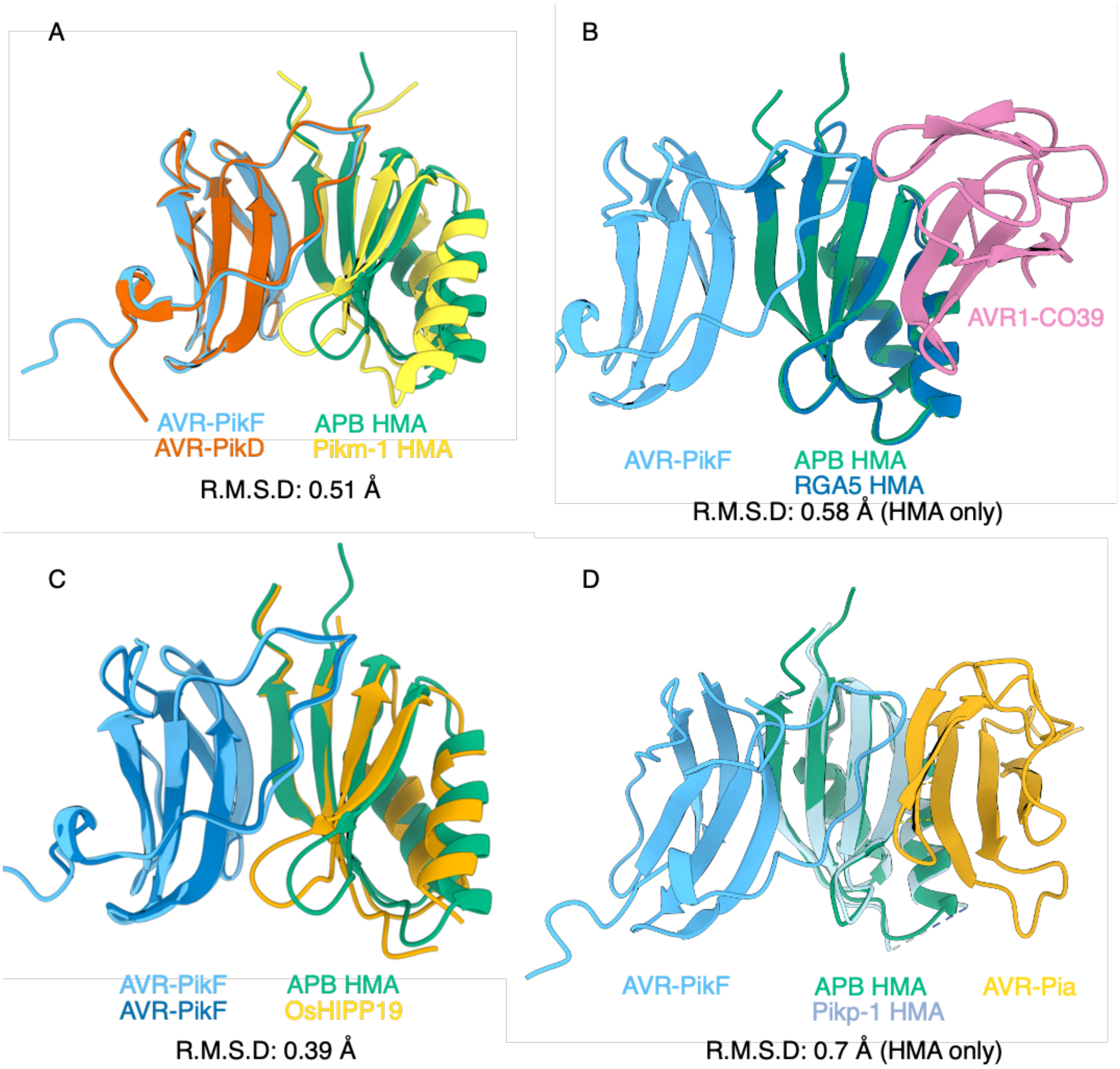

Supplemental Figure S9. Superimposition of the crystal structure of APB HMA/AVR-PikF complex with other MAX effector/HMA complexes. Supports Figure 5. A) Superimposition with Pikm-1 HMA/AVR-PikD (PDB ID: 6G10). **B)** Superimposition with RGA5 HMA/AVR1-CO39 (PDB ID: 5ZNG). **C)** Superimposition with OsHIPP19/AVR-PikF (PDB ID: 7B1I). **D)** Superimposition with Pikp-1 HMA/AVR-Pia (PDB ID: 6Q76).

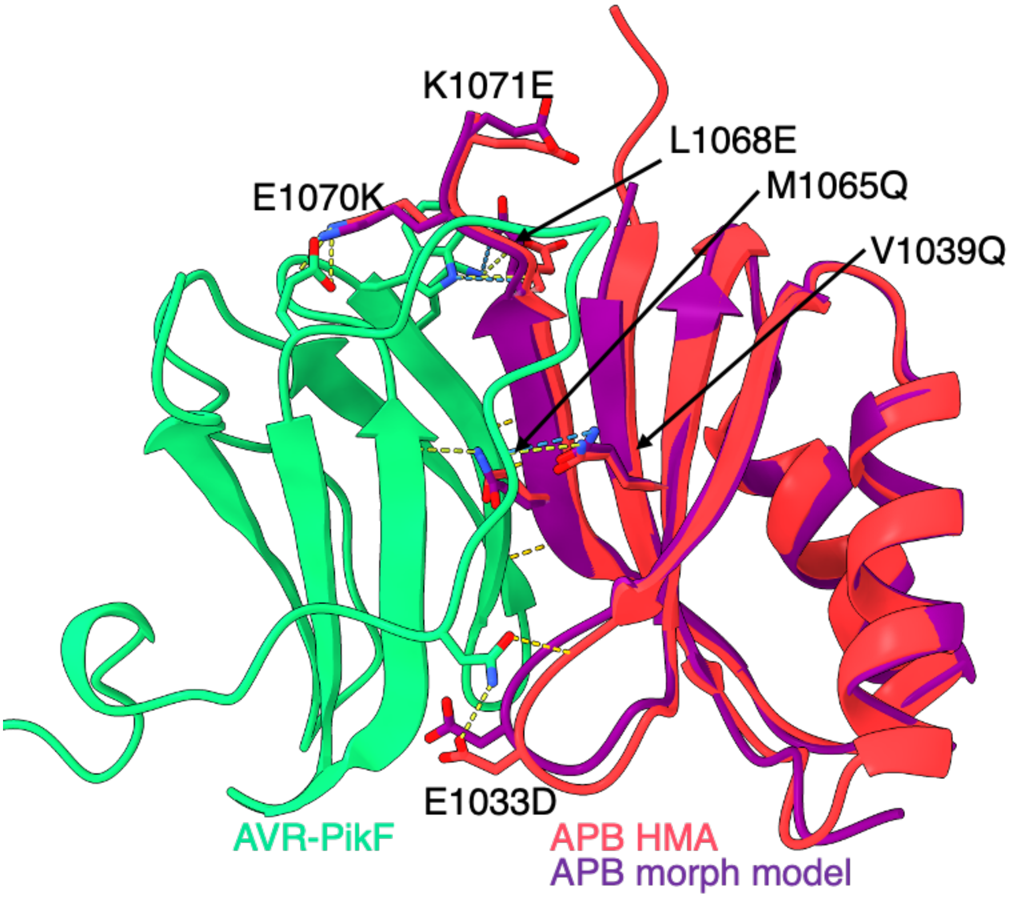

Supplemental Figure S10. Superimposition of the crystal structure of APB HMA/AVR-PikF complex with the morph model generated from an OsHIPP19 template. Supports Figure 5. Residues of RGA5 that were mutated based on OsHIPP19 to create the APB mutant are shown.

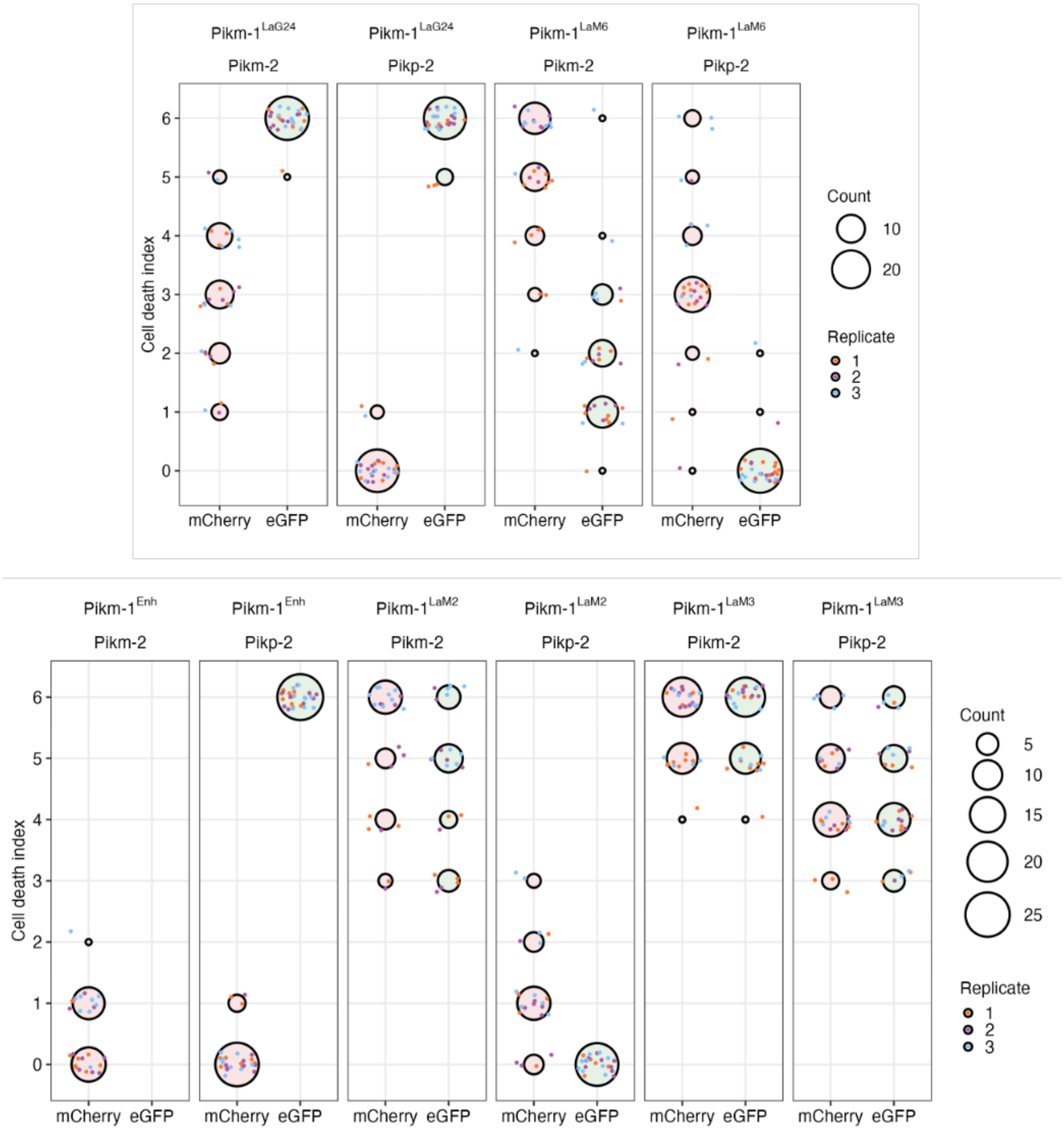

Supplemental Figure S11. Supporting figure for Figure 6. Cell death scoring of pikobodies co-expressed with Pikp-2 and Pikm-2 in *N. benthamiana*. Supports Figure 6. A) Cell death scoring of fluorescent protein-responding pikobodies co-expressed with the Pikm-2 and Pikp-2 helpers in *N. benthamiana* represented as dot plots. The total number of repeats was 30 per sample. For each sample, all the data points are represented as dots with a distinct color for each of the three biological replicates; these dots are jittered around the cell death score for visualization purposes. The size of the central dot at each cell death value is proportional to the number of replicates of the sample with that score. Statistical analyses of these results are shown in **Appendix 1 J (Supplemental File 1)**. Details of the NLR mutants used in these experiments can be found in **Supplemental Table S3**.

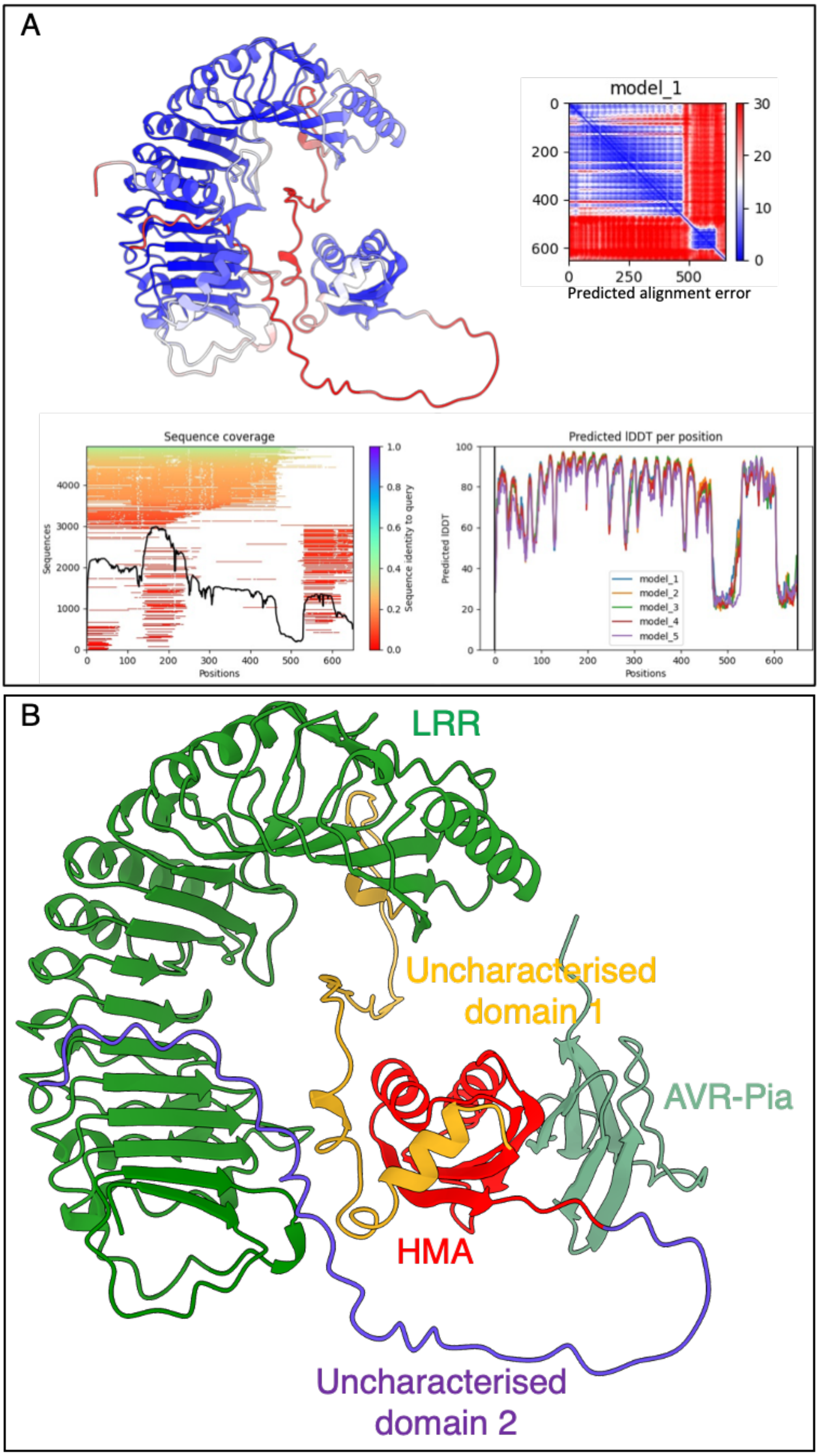
A
model_1
Predicted alignment error
Sequence coverage
Sequences
Sequence identity to query
Positions
Predicted lDDT per position
Predicted lDDT
model_1
model_2
model_3
model_4
model_5
B
LRR
Uncharacterised domain 1
AVR-Pia
HMA
Uncharacterised domain 2

Supplemental Figure S12. AlphaFold2 prediction of the C-terminal domains of RGA5 describes previously uncharacterized domains. A) AlphaFold2 v2.1 (Jumper et al., 2021) (as implemented in the AlphaFold ColabFold (Mirdita et al., 2022)) prediction of the RGA5 C-terminus. The RGA5 model is colored by residue position confidences, with blue indicating high confidence and red low confidence. The LRR and HMA domains appear well predicted, however additional regions at the C-terminus lack confidence. **B)** Coloring of the RGA5 AlphaFold2 model to highlight the different domains present, with AVR-Pia (olive green) superimposed at the predicted HMA interface (from PDB ID: 6Q76).

Supplemental Table S1. Full kinetic parameters for HMA-effector interactions as measured by SPR.

Effector	k_a (M^{-1} s^{-1})	k_d (s^{-1})	K_D (M)	R-max	U-value	$\chi 2$
			Pikm-1			
AVR-PikD	5.77E+05	5.74E-03	9.96E-09	134.3	2	1.86E+00
AVR-PikC	8.03E+04	6.99E-01	8.70E-06	120.8	20	3.21E+00
AVR-PikF	9.65E+04	5.89E-01	6.10E-06	144.1	15	6.72E+00
AVR-Pia	3.72E+01	6.95E-01	1.87E-02	16647.9	95	1.90E+01
AVR-Pii	N.B.	N.B.	N.B.	N.B.	N.B.	N.B.
			RGA5			
AVR-PikD	3.79E+04	5.42E-01	1.43E-05	833.3	15	7.07E+01
AVR-PikC	1.81E+04	1.22E+00	6.73E-05	706.6	20	2.51E+00
AVR-PikF	8.13E+03	5.40E-01	6.64E-05	240.3	33	4.98E+00
AVR-Pia	3.29E+04	8.82E-01	2.68E-05	774.1	15	4.58E+01
AVR-Pii	N.B.	N.B.	N.B.	N.B.	N.B.	N.B.
			APB			
AVR-PikD	5.30E+05	1.67E-04	3.14E-10	40.4	5	7.77E-01
AVR-PikC	3.35E+05	9.87E-04	2.95E-09	31.6	2	4.52E-01
AVR-PikF	6.23E+04	1.02E-03	1.65E-08	49.4	2	5.64E-01
AVR-Pia	2.33E+04	7.66E-01	3.29E-05	502.3	33	6.42E+01
AVR-Pii	N.B.	N.B.	N.B.	N.B.	N.B.	N.B.

Supplemental Table S2. Data collection and refinement statistics

	APB/AVR-PikF
Data collection statistics	
Wavelength (Å)	0.979
Space group	$P\ 2_1\ 2_1\ 2_1$
Cell dimensions *a*, *b*, *c* (Å)	31.91 57.90 76.66
Resolution (Å)*	46.24 – 1.22 (1.24 – 1.22)
R_{merge} (%)	6.9 (188.4)
$I/\sigma I$	14.5 (0.7)
Completeness (%)	
Overall	99.4 (92.0)
Anomalous	99.0 (88.6)
Unique reflections	42985 (1915)
Redundancy	
Overall	11.5 (5.2)
Anomalous	6.0 (2.7)
CC(1/2) (%)	100 (32.4)
Refinement and model statistics	
Resolution (Å)	46.24 – 1.22 (1.24 – 1.22)
R_{work}/R_{free} (%)	14.9 / 20.2
No. atoms	1516
Protein	1324
Ligand	9
Ion	2
Water	181
B-factors	
Protein	18.0
Ligand	33.6
Ion	22.1
Water	27.2
R.m.s deviations	
Bond lengths (Å)	0.0129
Bond angles (°)	1.88
Ramachandran plot (%)**	
Favoured	97.50
Allowed	1.88
Outliers	0.62
MolProbity Score	1.48

*The highest resolution shell is shown in parenthesis.
**As calculated by MolProbity

Supplemental table 3. Mutants used in this study.

Name	Background	ID/Mutation	Reference
Pikp-1$^{\Delta HMA}$	Pikp-1	NOI (Pii-2 residues Glu1016 to Lys1052)	This study
Pikp-2^{D230E}	Pikp-2	Asp230Glu	De la Concepcion et al., 2021b
Pikp-2^{T434S}	Pikp-2	Thr434Ser	De la Concepcion et al., 2021b
Pikp-2^{M627V}	Pikp-2	Met627Val	De la Concepcion et al., 2021b
Pikm-2^{E230D}	Pikm-2	Glu230 Asp	De la Concepcion et al., 2021b
Pikm-2^{S434T}	Pikm-2	Ser434Thr	De la Concepcion et al., 2021b
Pikm-2^{V627M}	Pikm-2	Val627Met	De la Concepcion et al., 2021b
Pikp-1^{mHMA}	Pikp-1	Pikm HMA	This study
Pikm-1^{pHMA}	Pikm-1	Pikp HMA	This study
Pikm-1$^{\beta 1}$	Pikm-1	Pikm HMA (Pikp HMA residues Arg183 to Met197)	This study
Pikm-1$^{\alpha 1}$	Pikm-1	Pikm HMA (Pikp HMA residues Glu198 to Gly215)	This study
Pikm-1$^{\beta 2}$	Pikm-1	Pikm HMA (Pikp HMA residues Val216 to Leu225)	This study
Pikm-1$^{\beta 3}$	Pikm-1	Pikm HMA (Pikp HMA residues Arg226 to Ile236)	This study
Pikm-1$^{\alpha 2}$	Pikm-1	Pikm HMA (Pikp HMA residues Asp237 to Lys248)	This study
Pikm-1$^{\beta 4}$	Pikm-1	Pikm HMA (Pikp HMA residues Val249 to Lys262)	This study
Pikm-1^{I184K}	Pikm-1	Ile184Lys	This study
Pikm-1^{M185T}	Pikm-1	Met185Thr	This study
Pikm-1^{DG186K}	Pikm-1	ΔΓλψ186	This study
Pikm-1^{E188L}	Pikm-1	Glu188Leu	This study
Pikm-1^{M189K}	Pikm-1	Met189Lys	This study

Name	Background	ID/Mutation	Reference
Pikm-1^{I196V}	Pikm-1	Ile196Val	This study
Pikm-1^{P197A}	Pikm-1	Pro197Ala	This study
Pikm-1^{S239P}	Pikm-1	Ser239Pro	This study
Pikm-1^{N241K}	Pikm-1	Asn241Lys	This study
Pikm-1^{V243I}	Pikm-1	Val243Ile	This study
Pikm-1^{P252D}	Pikm-1	Pro252Asp	This study
Pikm-1^{M254E}	Pikm-1	Met254Glu	This study
Pikm-1^{F255L}	Pikm-1	Phe255Leu	This study
Pikm-1^{E257Q}	Pikm-1	Glu257Gln	This study
Pikm-1^{V261A}	Pikm-1	Val261Ala	This study
Pikm-1^{K262N}	Pikm-1	Lys262Asn	This study
Pikm-1^{E263K}	Pikm-1	Glu263Lys	This study
Pikm-1^{RGA5}	Pikm-1	RGA 5 HMA (residues 997 to 1071)	This study
Pikm-1^{APB}	Pikm-1	Glu1033Asp, Val1039Gln, Met1065Gln, Leu1068Glu, Glu1070Lys, Lys1071Glu	This study
Pikm-1^{LaG24}	Pikm-1	LaG24 anti-GFP nanobody	Fridy et al. 2014; Kourelis et al., 2023
Pikm-1^{Enhancer}	Pikm-1	Enhancer/cAbGFP4 anti-GFP nanobody	Fridy et al. 2014; Kourelis et al., 2023
Pikm-1^{LaM2}	Pikm-1	LaM2 anti-mCherry nanobody	Fridy et al. 2014; Kourelis et al., 2023
Pikm-1^{LaM3}	Pikm-1	LaM3 anti-mCherry nanobody	Fridy et al. 2014; Kourelis et al., 2023
Pikm-1^{LaM6}	Pikm-1	LaM6 anti-mCherry nanobody	Fridy et al. 2014; Kourelis et al., 2023

Supplemental Table S4. Key resources used in this study.

Reagent type (species) or resource	Designation	Source or reference	Identifiers	Additional Information
Recombinant DNA reagent	pICH47742	Addgene; (Engler et al., 2014)		
Recombinant DNA reagent	pICSL01005	Addgene; (Engler et al., 2014)		
Recombinant DNA reagent	Pikm-1 DOM2 acceptor	This paper		To create Pikm-1 chimeras
Recombinant DNA reagent	pICH47751	Addgene; (Engler et al., 2014)		
Recombinant DNA reagent	pPGN-C	Addgene; (Bentham et al., 2021)		
Recombinant DNA reagent	pPGC-K	Addgene; (Bentham et al., 2021)		
Recombinant DNA reagent	pICSL4723	Addgene; (Engler et al., 2014)		
Commercial assay or kit	ANTI-FLAG M2 Affinity Magnetic Beads	Sigma (Merk)	A2220	
Commercial assay or kit	Series S Sensor Chip CM5	Cytiva	29104988	
Antibody	Anti-FLAG M2 antibody (mouse monoclonal)	Signma (Merk)	Cat. #F1804	Used diluted (1:5000)
Antibody	Anti-MYC (9E10) (mouse monoclonal)	Santa Cruz Biotechnology	sc-40	Used diluted (1:3000)
Antibody	Anti-mouse IgG HRP conjugate	Promega	Cat. #W4021	Used diluted (1:10000)
Commercial assay or kit	ECL extreme Lumiblue Western Blotting Substrate	Abcam	Ab270517	
Commercial assay or kit	SG1 Screen, spare matrix crystallisation screen	Molecular Dimensions	MD1-88	
Software, algorithm	besthr R package	De la Concepcion et al., 2019; MacLean, 2019		
Software,				

algorithm	ggplot2 R package
Software, algorithm	ggpubr R package

Supplemental Table S5. Primers used in this study.

Primer Name	Sequence	Usage
1841JCmHMAβ1 _F	AAAAAATCGTGTTCAAGATTCCCATG	phosphorylated primers for site-directed mutagenesis in Pikm-HMA β1
1842JCmHMAP197A_F	AAAAAATCGTGTTCAAGATTGCCATG	phosphorylated primers for site-directed mutagenesis in Pikm-HMA β1
1843JCmHMAI196V_F	AAAAAATCGTGTTCAAGGTTCCCATG	phosphorylated primers for site-directed mutagenesis in Pikm-HMA β1
1844JCmHMAF194I_F	AAAAAATCGTGATCAAGATTCCCATG	phosphorylated primers for site-directed mutagenesis in Pikm-HMA β1
1845JCmHMA β1_R	GCATTTCCCCTCCCATTATTCTGAGA	phosphorylated primers for site-directed mutagenesis in Pikm-HMA β1
1846JCmHMAM189K_R	GCTTTTCCCCTCCCATTATTCTGAGA	phosphorylated primers for site-directed mutagenesis in Pikm-HMA β1
1847JCmHMAE188L_R	GCATTAGCCCTCCCATTATTCTGAGA	phosphorylated primers for site-directed mutagenesis in Pikm-HMA β1
1848JCmHMAΔG186_R	GCATTTCCCCCATTATTCTGAGA	phosphorylated primers for site-directed mutagenesis in Pikm-HMA β1
1849JCmHMAM185T_R	GCATTTCCCCTCCTGTTATTCTGAGA	phosphorylated primers for site-directed mutagenesis in Pikm-HMA β1
1850JCmHMAI184K_R	GCATTTCCCCTCCCATTTTTCTGAGA	phosphorylated primers for site-directed mutagenesis in Pikm-HMA β1
1559JCmHMAS239P_R	TGGGGTCAATGCCATCAC	phosphorylated primers for site-directed mutagenesis in Pikm-HMA α2
1560JCmHMAα2_R	TGGAGTCAATGCCATCAC	phosphorylated primers for site-directed mutagenesis in Pikm-HMA α2
1561JCmHMAα2_F	TCAATCTTGTCTCTGCGCTC	phosphorylated primers for site-directed mutagenesis in Pikm-HMA α2
1562JCmHMAN241K_F	TCAAGCTTGTCTCTGCGCT	phosphorylated primers for site-directed mutagenesis in Pikm-HMA α2
1563JCmHMAV243I_F	TCAATCTTATCTCTGCGCTC	phosphorylated primers for site-directed mutagenesis in Pikm-HMA α2

1565JCmHMAβ4_R	CGCAGGGCCCACCTT	phosphorylated primers for site-directed mutagenesis in Pikm-HMA β4
1566JCmHMAP252D_R	CGCATCGCCCACCTTC	phosphorylated primers for site-directed mutagenesis in Pikm-HMA β4
1567JCmHMAβ4_F	ATGTTTCTGGAGGTCAGCCA	phosphorylated primers for site-directed mutagenesis in Pikm-HMA β4
1568JCmHMAM254E_F	GAGTTTCTGGAGGTCAGCCA	phosphorylated primers for site-directed mutagenesis in Pikm-HMA β4
1569JCmHMAF255L_F	ATGTTGCTGGAGGTCAGCCA	phosphorylated primers for site-directed mutagenesis in Pikm-HMA β4
1570JCmHMAE257Q_F	ATGTTTCTGCAGGTCAGCCA	phosphorylated primers for site-directed mutagenesis in Pikm-HMA β4
1571JCmHMAV261A_F	CAAAGGAGGACGTGAGAC	phosphorylated primers for site-directed mutagenesis in Pikm-HMA β4
1572JCmHMAK262N_F	TAAATGAGGACGTGAGAC	phosphorylated primers for site-directed mutagenesis in Pikm-HMA β4
1573JCmHMAE263K_F	TAAAGAAAGACGTGAGACCAA	phosphorylated primers for site-directed mutagenesis in Pikm-HMA β4
1574JCmHMA β4_2R	CTTGGCTGACCTCCAG	phosphorylated primers for site-directed mutagenesis in Pikm-HMA β4
