# Supplementary material for: Allelic compatibility in plant immune receptors facilitates engineering of new effector recognition specificities
Source: Plant Cell. 2023 Jul 24;35(10):3809–27. doi: 10.1093/plcell/koad204 (PMC10533329; doi:10.1093/plcell/koad204)
Supplement: koad204_Supplementary_Data [file koad204_supplementary_data.zip › tpc.23.00251Supplemental File 1 Appendices.pdf]

Appendix 1. Statistical analysis of cell death scoring with besthr.

Cell death scoring in this study was performed through qualitative measurement of cell death as determined by approximation of autofluorescence under UV light 5 dpi, as previously performed in De la Concepcion et al., 2019. The autofluorescence was compared to a previously established cell death scale (Maqbool et al., 2015). To analyse our cell death scoring, we used estimation methods (Ho et al., 2019) and visualised these with use of the besthr R package (MacLean, 2019) to generate estimation graphics.

Besthr compares the cell death scores of all samples and ranks them irrespective of sample then generates mean ranks for the control and test samples. A bootstrap process is then performed on the ranked test data in which samples of equal size to the experiment were replaced and a mean rank is calculated.

Rank means were calculated after 1000 bootstrap samples and a distribution of the mean ranks were plotted, with the 2.5 and 97.5 quantiles calculated and highlighted on the plotted distribution. If the mean of the control data is outside of the 2.5 or 97.5 quantile boundaries, the control and test means are considered to be different.

References

De la Concepcion, J.C., Franceschetti, M., MacLean, D., Terauchi, R., Kamoun, S., Banfield, M.J., 2019. Protein engineering expands the effector recognition profile of a rice NLR immune receptor. eLife 8, e47713. https://doi.org/10.7554/eLife.47713

Ho, J., Tumkaya, T., Aryal, S., Choi, H., Claridge-Chang, A., 2019. Moving beyond P values: data analysis with estimation graphics. Nat Methods 16, 565–566. https://doi.org/10.1038/s41592-019-0470-3

MacLean, D., 2019. TeamMacLean/besthr: Initial Release. Zenodo. https://doi.org/10.5281/zenodo.3374507

Maqbool, A., Saitoh, H., Franceschetti, M., Stevenson, C., Uemura, A., Kanzaki, H., Kamoun, S., Terauchi, R., Banfield, M., 2015. Structural basis of pathogen recognition by an integrated HMA domain in a plant NLR immune receptor. eLife 4, e08709. https://doi.org/10.7554/eLife.08709

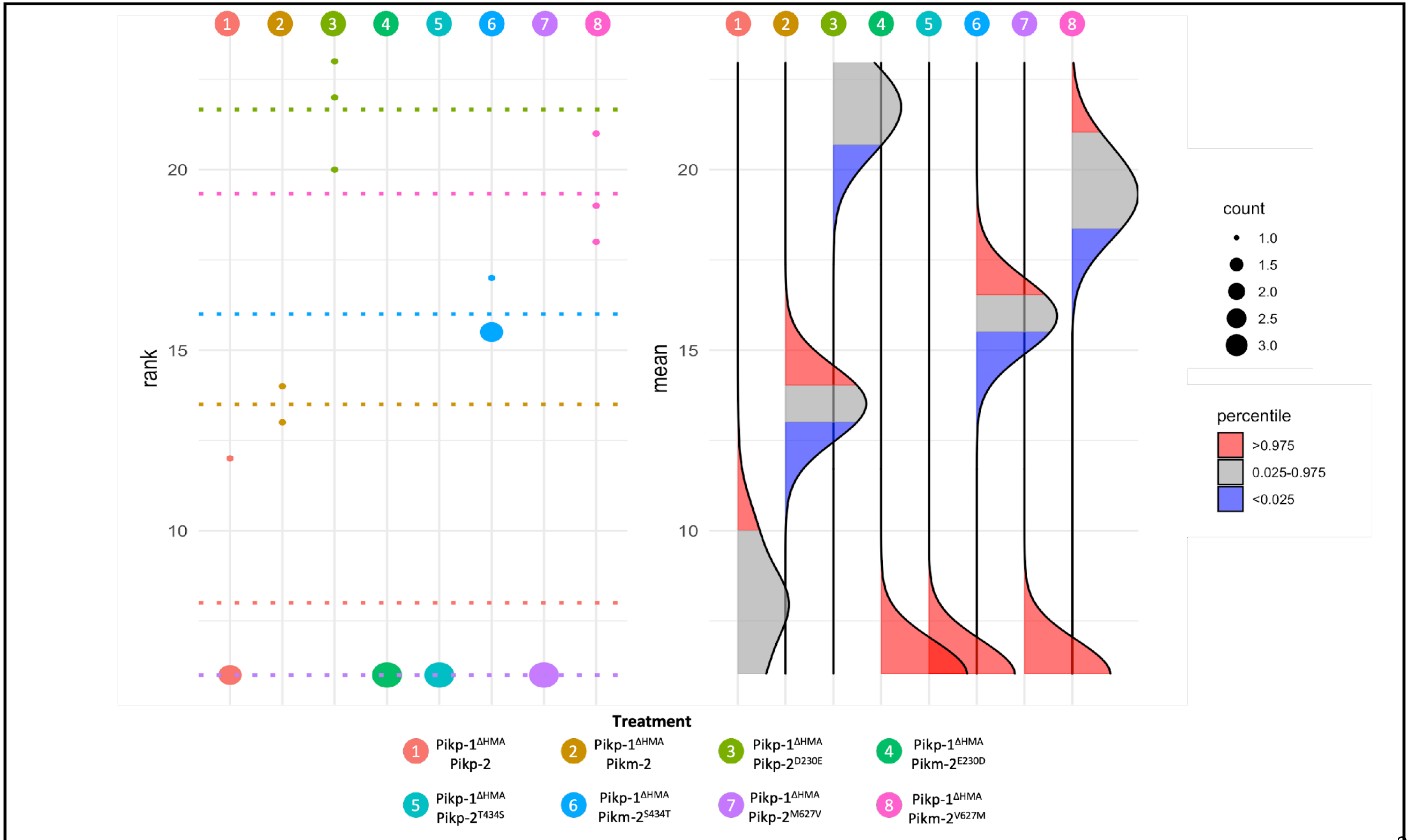

Appendix 1 A. Statistical analysis of cell death scoring from Figure 1.

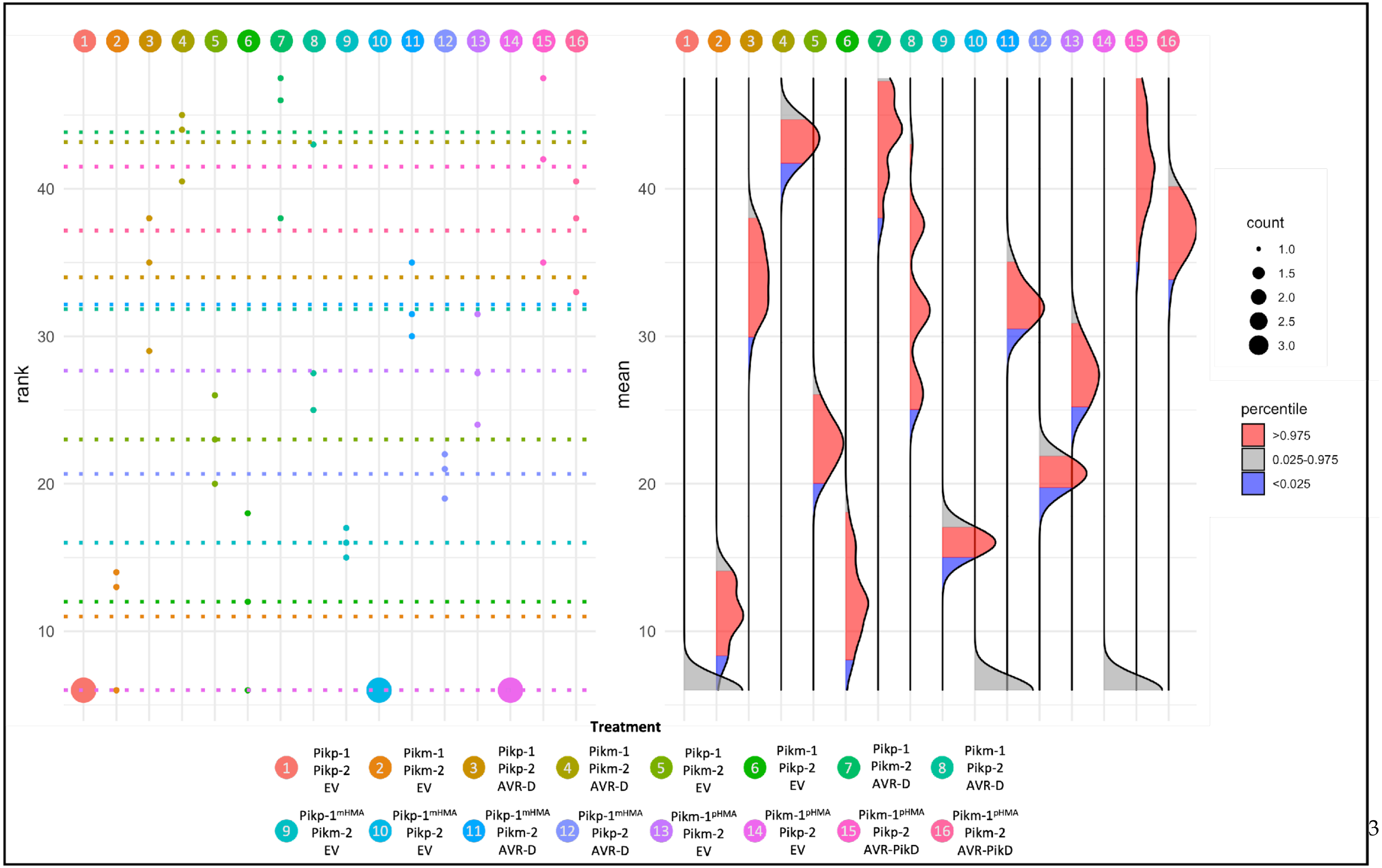

Appendix 1 B. Statistical analysis of cell death scoring from Figure S1 A.

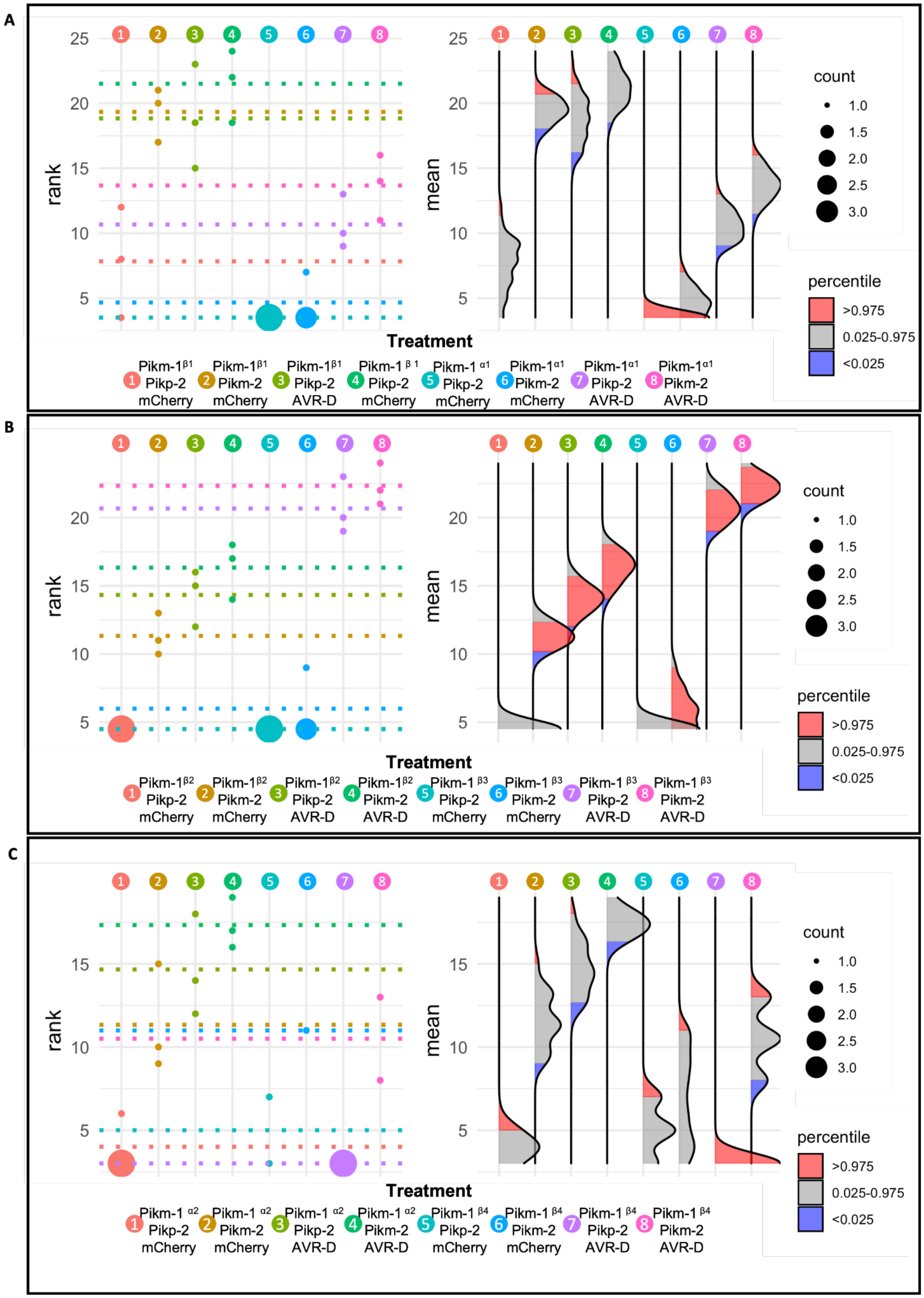

Appendix 1 C. Statistical analysis of cell death scoring from Figure S1 B. A) Chimeras of Pikm-1 with the β1 and α1 secondary structures of Pikp-1 HMA **B)** Chimeras of Pikm-1 with the β2 and β3 structures of Pikp-1 **C)** Chimeras of Pikm-1 with the α2 and β4 secondary structures of Pikp-1 HMA

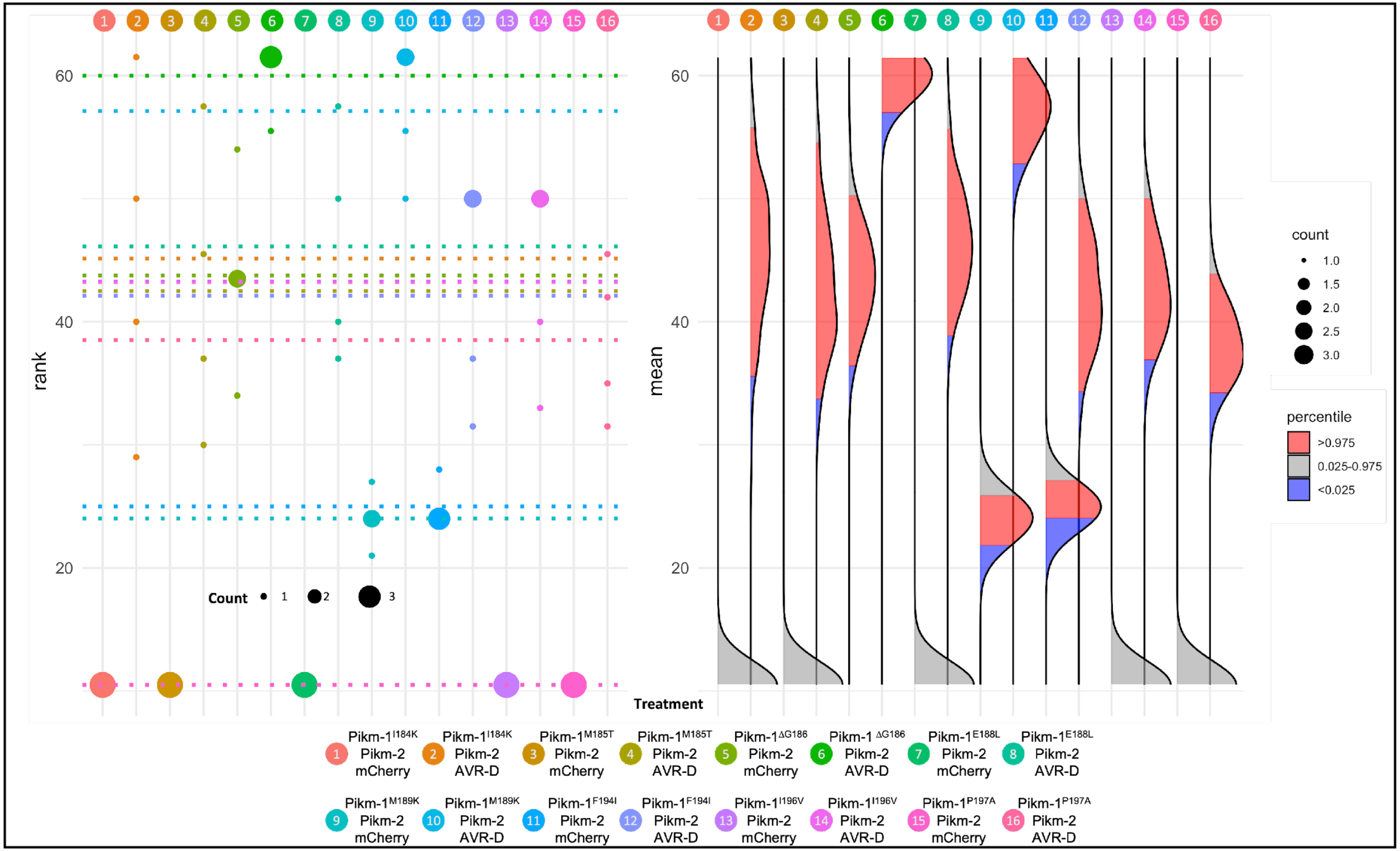

Appendix 1 D. Statistical analysis of cell death scoring from Figure S3 A.

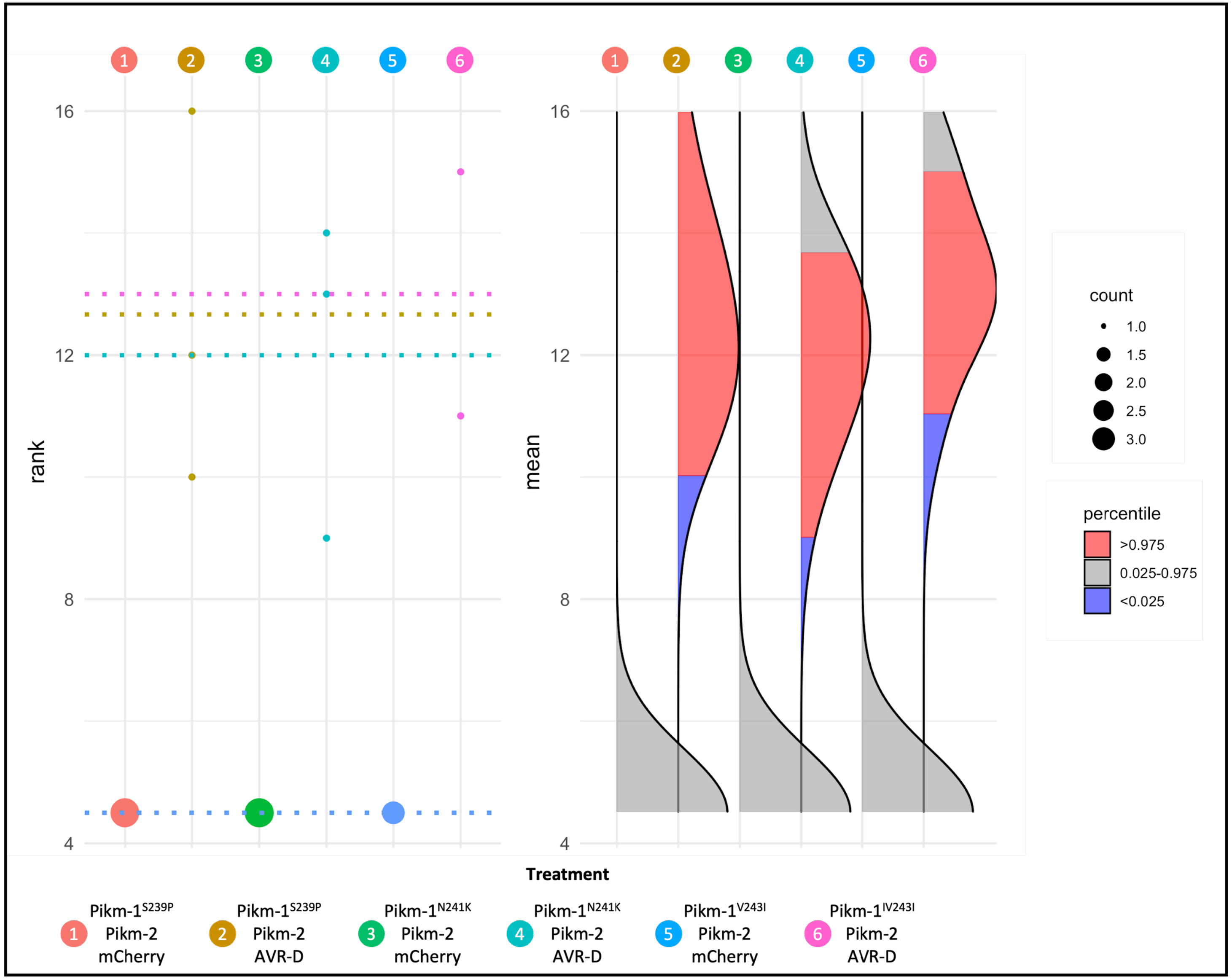

Appendix 1 E. Statistical analysis of cell death scoring from Figure S3 B.

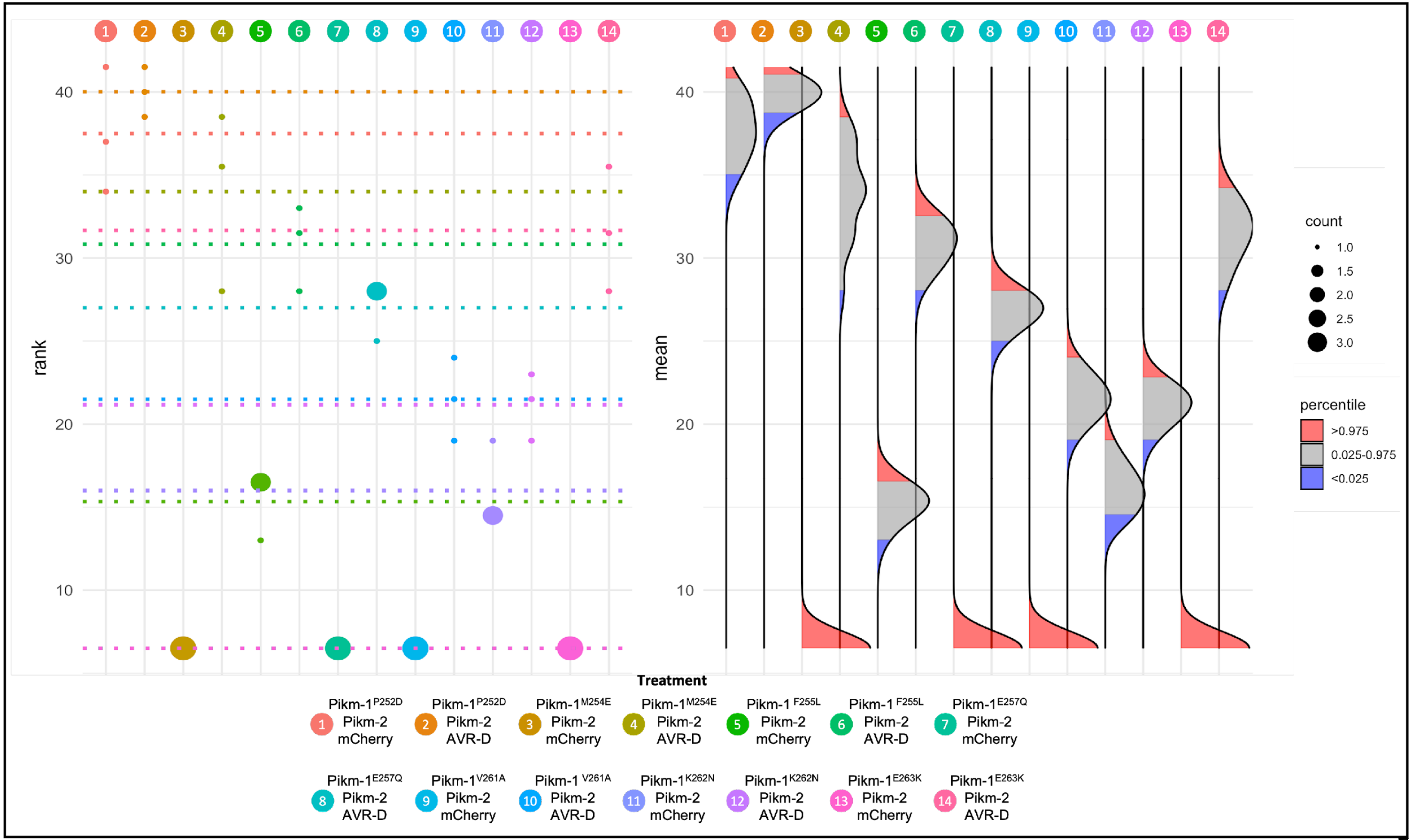

Appendix 1 F. Statistical analysis of cell death scoring from Figure S3 C.

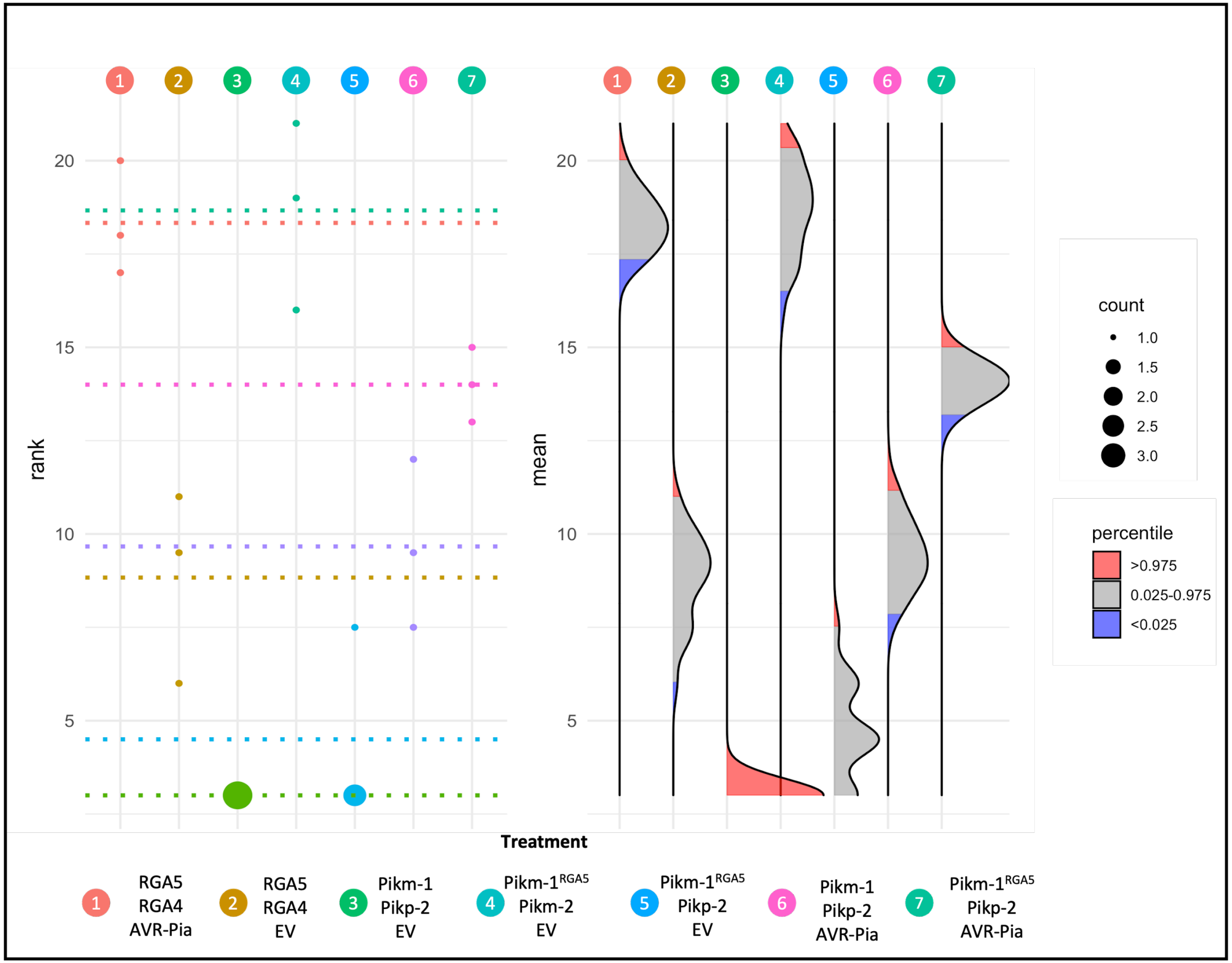

Appendix 1 G. Statistical analysis of cell death scoring from Figure 3 B.

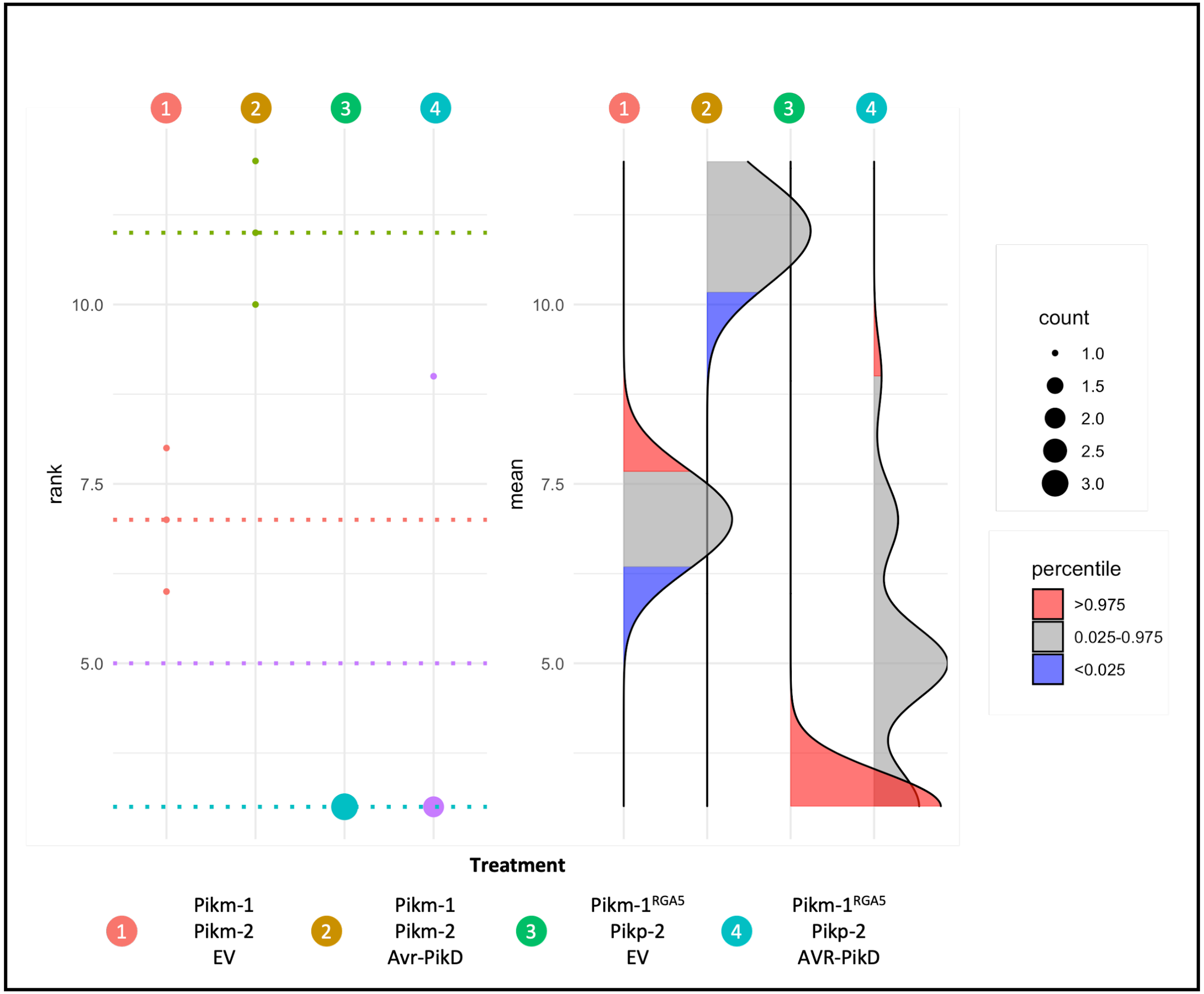

Appendix 1 H. Statistical analysis of cell death scoring from Figure S4.

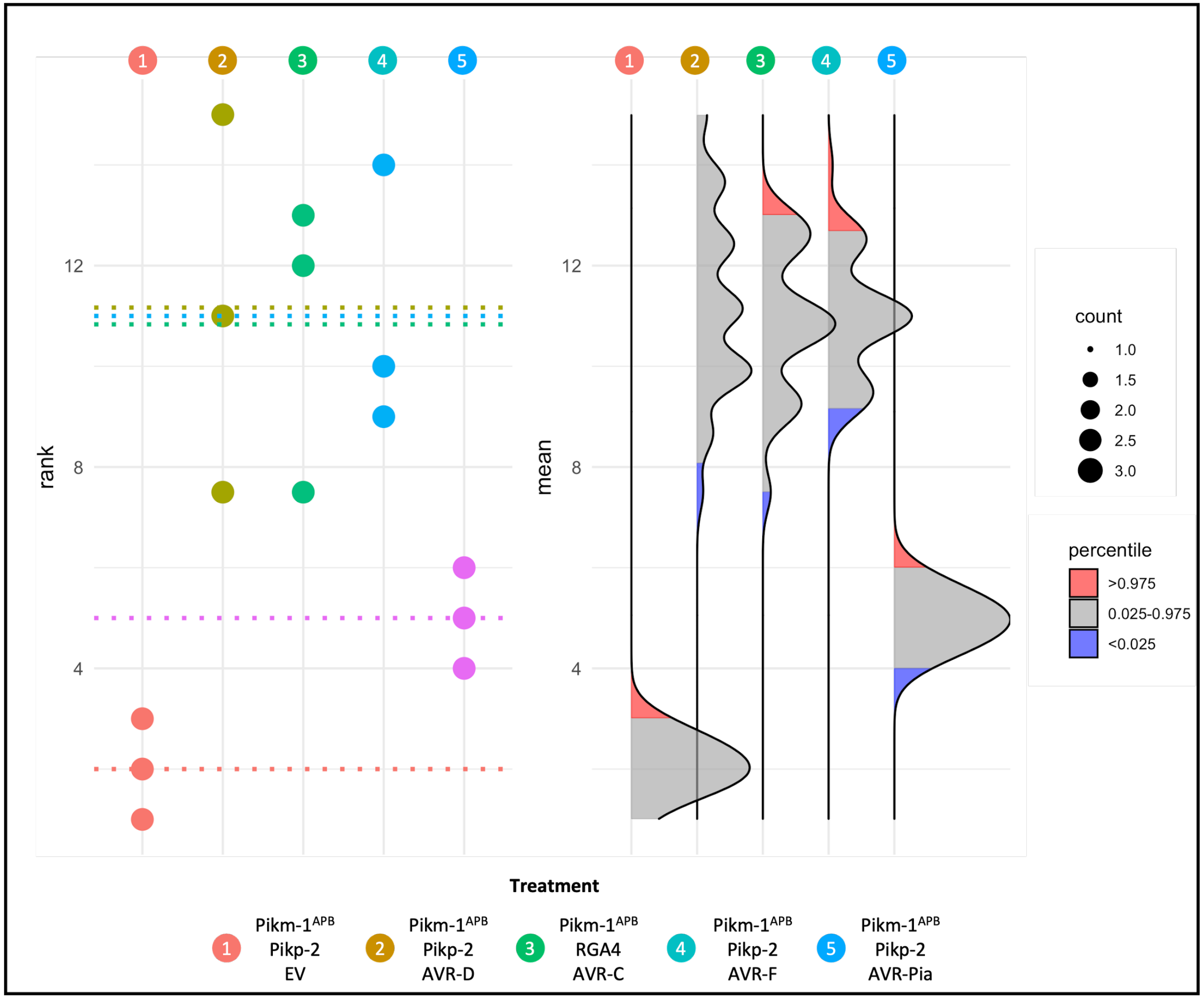

Appendix 1 I. Statistical analysis of cell death scoring from Figure 4.

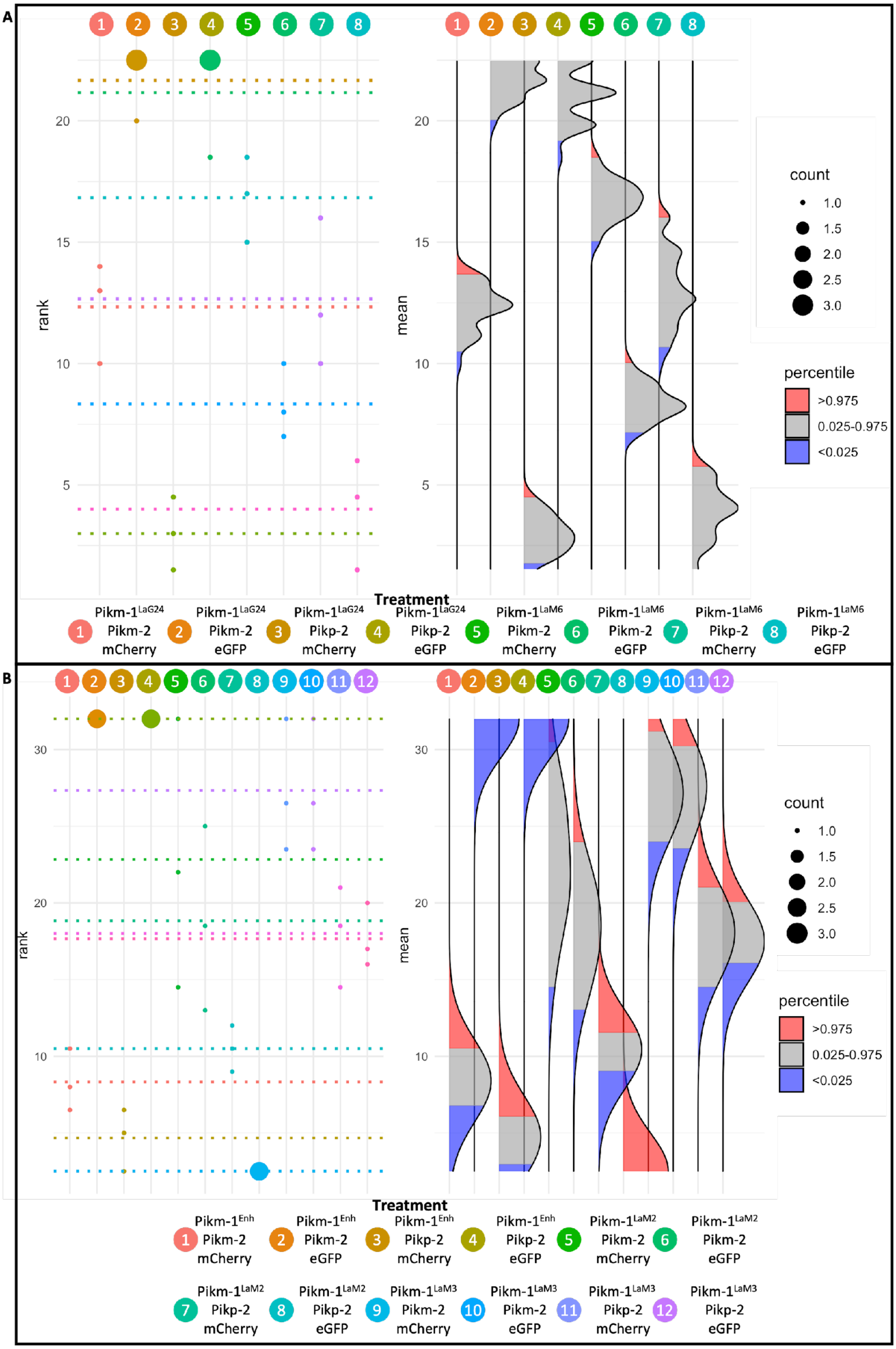

Appendix 1 J. Statistical analysis of cell death scoring from Figure S11
