# Supplementary material for: Allelic compatibility in plant immune receptors facilitates engineering of new effector recognition specificities
Source: Plant Cell. 2023 Jul 24;35(10):3809–27. doi: 10.1093/plcell/koad204 (PMC10533329; doi:10.1093/plcell/koad204)
Supplement: koad204_Supplementary_Data [file TPC2023RA00251R1 Peer Review Report.pdf › TPC2023RA00251R1 Peer Review Report.pdf]

Allelic compatibility in plant immune receptors facilitates engineering of new effector recognition specificities

Adam R. Bentham, Juan Carlos De la Concepcion, Javier Vega Benjumea, Jiorgos Kourelis, Sally Jones, Melanie Mendel, Jack Stubbs, Clare E. M. Stevenson, Josephine H.R. Maidment, Mark Youles, Rafał Zdrzałek, Sophien Kamoun, Mark J. Banfield

Corresponding author: *Mark J Banfield mark.banfield@jic.ac.uk*

Review timeline:

**TPC2023-RA-00251**	Submission received:	March 20, 2023
	1st Decision:	May 6, 2023, *accept with minor revisions*
**TPC2023-RA-00251R1**	1st Revision received:	June 1, 2023
	2nd Decision:	June 5, 2023 *acceptance pending, sent to science editor*
	Final acceptance:	July 10, 2023

REPORT: (The report shows the major requests for revision and author responses. Minor comments for revision and miscellaneous correspondence are not included. The original format may not be reflected in this compilation, but the reviewer comments and author responses are not edited, except to correct minor typographical or spelling errors that could be a source of ambiguity.)

TPC2023-RA-00251 1st Editorial decision – *accept with minor revisions* **May 6, 2023**

On the basis of the advice received, the board of reviewing editors would like to accept your manuscript for publication in The Plant Cell. The three reviewers all provided minor suggestions for improvement of the text, but did not request any additional experiments. Please incorporate these suggestions as you see fit in your revision. Congratulations on these very positive reviews!

--- Reviewer comments:

Reviewer #1 (Comments for the Author):

The authors demonstrate HMA domains integrated in Pik-1 not only evolved to bind pathogen effectors but also likely co-evolved with other NLR domains to maintain immune homeostasis. They integrate the RGA5 HMA domain into mismatching pairs Pikm-1/Pikp-2, enabling Pikm-1APB to recognize multiple AVR-Pik variants and show the interaction interface of mutant RGA5 HMA domain by solving the crystal structure of APB domain with AVR-PikF. Overall, this is a very well-written manuscript exploiting the allelic diversity in the Pik NLR pair to allow for the generation of receptors with expanded recognition specificities and providing a significant advance toward structure-guided designing of sensor Pik to recognize multiple effectors. There is no comment to strengthen the current manuscript.

Reviewer #2 (Comments for the Author):

This manuscript extends a body of work from this group exploring how to engineer novel resistance specificities into the paired NLR Pik-1/-2 resistance locus based on detailed structural knowledge of the Pik-1-AvrPik interaction. Here the authors show that the autoactivity of some engineered Pikm-1 receptors with change ot or substitutionof the integrated HMA domain can be alleviated by expressing them with the pikp-2 allele of the helper NLR instead of the pikm-2 allele that naturally occurs with pikm-1. They define specific sites within the pikp,m-2 alleles and in the HMA domain that determine this compatibility/incompatibility between allelic pairs. This is an important study that helps to define how paired receptors work together as well as principles for effective NLR engineering.

Below I list some minor points for the authors to consider:

1. Line145. The reference in this sentence to 'autoactivity triggered by engineering' is a bit vague in this context. I assume it mainly refers autoactivity of some of the variants with nanobody insertions in Kourelis et al 2023 which is

directly addressed in the results. It would be better to describe that result explicitly in the introduction as a motivation for the current study.

2. Line 164-78, Figure 1. This figure includes results for substitutions at positions 434 and 627 but these are not described in the text. What is their significance?

3. Line 201. This sentence is missing some words to describe what the chimeric receptors contain, ie swaps of the individual beta strands and alpha helices from Pikp-1 into Pikm-1.

4. Line 205. In addition to not all of the aas in b1 and b2 making contacts, none of the a2 residues do so either. I note from this experiment that the a1, b2 and b4 swaps also lose recognition. Is this expected?

5. Line 219. The potential for large structural changes with these two substitutions is reasonable, but perhaps there is evidence for this from existing structure data of these domains or modelling/prediction that could be cited?

6. Line 246. It is not clear what is the significance of the RGA5-HMA substitution cell death being 'comparable to the cross-reactivity of pikp1-pikp-2 with Avr-Pia". This comparison is not included in the figure in any case. If the point is that this low level of response may not translate into resistance as pikp does not provide resistance to strain with AvrPia, then perhaps it would be more appropriate to elaborate on this in the discussion?

7. Line 270. The similarity in AvrPia recognition between the RGA5 and APB is consistent with the different binding sites for AvrPia and AvrPik isn't it?

8. Line 282 onwards. While the lower affinity of RGA5-HMA for AvrPia is a reasonable explanation for the weaker HR response, another possibility to consider is that the different binding site is simply not located in the right position to favour the active state of the Pik-1/2 receptor complex over the inactive state. The data in Table 1 is quite important for this section and I think it would be better as a main table rather than a supplementary table.

9. Line 347/Figure S10. I couldn't see figure S10, but I think it might be hiding behind figure S11 superimposed on top of it.

10. Line 354 - this sentence describing the autoactive nanobody fusions could go into the introduction - see comment above.

11. Line 367. Worth noting that LaM3 was still autoactive even with pikp-2.

12. Line 399. This sentence is not entirely clear. "different threshholds" for what?

13. Line 412. The authors state that the pikm1-/pikp-2 pairing is superior to either a pikp-1/pikp-2 or pikm-1/pikm-2 pair for engineering. However there is no data in this manuscript for engineering of the pikp-1 HMA domain or its compatibility with either pikp-2 or pikm-2. The only related data I can see is in figure 2A where pikp-1 containing the mHMA domain is shown to function equally well with either pikp-2 or pikm-2. Have other modifications of pikp-1 been described elsewhere as leading to autoactivity? If so, make that statement explicitly and include a citation, but if not then pikp1/2 should not be excluded as an unsuitable pairing.

14. Line 416. Insert that the pikm-1-VHH-nanobody fusions with autoactive in the presence of pikm-2

15. Line 448 to 454. This could be rephrased as the logic is not consistent. First it is noted that the RGA5 and APB mutations give low levels of cell death and a possible explanation provided that the binding interface is occluded. Then it is stated that it is more likely due to the lower affinity because low levels of cell death were observed. Clearly that observation does not discriminate between these possibly explanations. Fine to suggest that low affinity is the reason, but that is based on the correlation of the response with the measured binding affinities.

16. Line 509, figure 1 legend. The legend for part B does not describe what is shown in the figure - I assume UV fluorescence images of leaves at some time point after Agroinfiltration. As noted above the 434 and 627 mutations are not mentioned in the text or the legend.

Reviewer #3 (Comments for the Author):

Authors opened a possible strategy for NLR-mediated resistance engineering by using the allelic diversity in Pik NLR pair to create receptors with expanded recognition specificities without causing constitutive cell death. Authors' structural, biophysical and in planta analyses clearly demonstrated a usefulness of the Pik system as a simple but efficient means for generating bespoke resistance. In addition, this work provides a clear basis for the incorporation of new effector recognition motifs into the Pik system. I believe that this work is well organized with convincing results and written very well. The reviewer would like to confirm several minor points.

(1) Figure 1B: it would be nice to add a brief description about the reciprocal mutations for the two other residues T434S and M627V in the main text.

(2) L229: Our results also suggest Pikp-2 may be more accommodating of changes in the Pik-1 sensor than Pikm-2, even tolerating the complete substitution of the integrated HMA by an unrelated domain without inducing autoactivity. Here, cite the result.

(3) Fig 6 B: I wonder if the Pikp-2 + Pikm-1-LaM6 results are correct (Supplementary S11 showed cell death in Pikm-1LaM6 + Pikp-2 + mCherry, which is not consistent with Fig 6. Adding a control experiment (absence of fluorescent proteins) would be better here. Also, I would expect some discussion on this issue about how to overcome such problems in engineering Pikobodies.

(4) Check the S10/S11 figures.

TPC2023-RA-00251R1 1st Revision received June 1, 2023

Reviewer comments and author responses:

Reviewer #1 (Comments for the Author):
The authors demonstrate HMA domains integrated in Pik-1 not only evolved to bind pathogen effectors but also likely co-evolved with other NLR domains to maintain immune homeostasis. They integrate the RGA5 HMA domain into mismatching pairs Pikm-1/Pikp-2, enabling Pikm-1APB to recognize multiple AVR-Pik variants and show the interaction interface of mutant RGA5 HMA domain by solving the crystal structure of APB domain with AVR-PikF. Overall, this is a very well-written manuscript exploiting the allelic diversity in the Pik NLR pair to allow for the generation of receptors with expanded recognition specificities and providing a significant advance toward structure-guided designing of sensor Pik to recognize multiple effectors. There is no comment to strengthen the current manuscript.

We thank the reviewer for their supportive comments of our manuscript.

Reviewer #2 (Comments for the Author):
This manuscript extends a body of work from this group exploring how to engineer novel resistance specificities into the paired NLR Pik-1/-2 resistance locus based on detailed structural knowledge of the Pik-1-AvrPik interaction. Here the authors show that the autoactivity of some engineered Pikm-1 receptors with change to or substitution of the integrated HMA domain can be alleviated by expressing them with the pikp-2 allele of the helper NLR instead of the pikm-2 allele that naturally occurs with pikm-1. They define specific sites within the pikp,m-2 alleles and in the HMA domain that determine this compatibility/incompatibility between allelic pairs. This is an important study that helps to define how paired receptors work together as well as principles for effective NLR engineering.

We thank the reviewer for their thorough review of our manuscript and in adding beneficial comments to several important areas of the manuscript.

Below I list some minor points for the authors to consider:

Line145. The reference in this sentence to 'autoactivity triggered by engineering' is a bit vague in this context. I assume it mainly refers autoactivity of some of the variants with nanobody insertions in Kourelis et al 2023 which is directly addressed in the results. It would be better to describe that result explicitly in the introduction as a motivation for the current study.

We agree with the reviewer this could be better addressed in the introduction. To improve clarity for this point, we have included the following sentences in the paragraph starting at ln 118: "In particular, the manipulation of integrated domains in paired NLRs often results in autoactivity. This is best exemplified by several studies of the Pik NLR pair, in which the integrated domain of the Pik-1 sensor was substituted for an engineered variant or entirely different domains, resulting in autoactivation of the receptor pair (Białas et al., 2021; Kourelis et al., 2023; Maidment et al., 2022).

Line 164-78, Figure 1. This figure includes results for substitutions at positions 434 and 627 but these are not described in the text. What is their significance?

We have edited line 169-171 to include information about the other polymorphism in Pik-2 to address the reviewers query. The text now reads: "The Pikp-2 and Pikm-2 helpers only differ by three polymorphisms: Asp230Glu, Thr434Ser and Met627Val. We made reciprocal mutants of Pikp-2 and Pikm-2 helpers for each of these polymorphisms, generating six mutants (Pikp-2^{D230E}, Pikp-2^{T434S}, Pikp-2^{M627V}; Pikm-2^{E230D}, Pikm-2^{S434T}, Pikm-2^{V627M}) and found autoactivity induced by co-expression of Pikp-1$^{\Delta HMA}$/Pikm-2 is determined by the Pik-2 Asp230Glu polymorphism (Pikp-2^{D230E}/Pikm-2^{E230D}) (Figure 1 – Appendix 1A) as previously described for wildtype Pik NLRs (De la Concepcion et al., 2021b)"

Line 201. This sentence is missing some words to describe what the chimeric receptors contain, ie swaps of the individual beta strands and alpha helices from Pikp-1 into Pikm-1.

We have updated the text to include the single mutants of Pikm-1 made. Please see like 209 of the revised manuscript which reads: "We generated three sets of single mutants in Pikm-1: the β1 mutants Pikm-1^{I184K}, Pikm-1^{M185T}, Pikm-1$^{\Delta G186}$, Pikm-1^{E188L}, Pikm-^{1M189K,} Pikm-1^{F194I}, Pikm-1^{I196V} and Pikm-1^{P197A}; the α2 mutants Pikm-1^{S239P}, Pikm-1^{N241K} and Pikm-1^{V243I}; and the β4 mutants Pikm-1^{P252D}, Pikm-1^{M254E}, Pikm-1^{F255L}, Pikm-1^{E257Q}, Pikm-1^{V261A}, Pikm-1^{K262N} and Pikm-1^{E263K}. These mutants were then co-expressed in *N. benthamiana* with Pikm-2 to test their effect on receptor compatibility."

Line 205. In addition to not all of the aas in b1 and b2 making contacts, none of the a2 residues do so either. I note from this experiment that the a1, b2 and b4 swaps also lose recognition. Is this expected?

We thank the reviewer for their observant comment. From our cell death analyses we see no significant difference in the AVR-PikD recognition with the Pikm b2 and Pikm a1 chimeras, however the reviewer is correct in their statement recognition of AVR-PikD is lost in the Pikm-1 b4. This was not expected, and we are now aware that it was not addressed in the text. We have added the following text to the paragraph to acknowledge this observation: "Furthermore, we note co-expression of Pikp-2 with the Pikm-1^{β4} prevented autoactivity caused by co-expression of this chimera with Pikm-2, however we observed a the Pikm-1^{β4} to have a reduced response to AVR-PikD when paired with Pikp-2 (Figure 2 B, Figure S1 B – Appendix 1 C). This result may be due to changes to the important interactions between the residues of the β4 strand with the effector, which are known to be more extensive in Pikm-1 than in Pikp-1 (De la Concepcion et al., 2018), or potentially due to the reduced sensitivity of Pikp-2 as a helper (De la Concepcion et al., 2021)."

Additionally, we have updated the Pikm-1 b1 panels in Fig 2 B with better representative images from that experiment as the ones previously selected gave the wrong impression of a differential response to AVR-PikD, as highlighted by the reviewer.

Line 219. The potential for large structural changes with these two substitutions is reasonable, but perhaps there is evidence for this from existing structure data of these domains or modelling/prediction that could be cited?

We are glad the reviewer agrees our assessment of the ΔG186 and Pro252 substitutions is reasonable. Unfortunately, there is little structural data to draw upon to confirm our speculations. G186 lies outside of the HMA domain fold in a loop that is disordered in constructs that have been previous crystallised, and it is difficult to assess the impacts of a Pro substitution. Our lab has previously endeavoured to predict the structure of the Pikm-1 receptor with AlphaFold2 (AF2) to understand how the HMA domain works in conjunction with the other domains of the receptor, however the models are not satisfactory enough to draw reasonable hypotheses from, and as such we have deemed using AF2 to try to understand the impacts of the ΔG186 and Pro252 substitutions unfeasible. This analysis could be included in this manuscript, however due to the large amount of data in this manuscript and the speculative nature of the analysis, we believe it would distract from the messages already in this manuscript.

Line 246. It is not clear what is the significance of the RGA5-HMA substitution cell death being 'comparable to the cross-reactivity of pikp1-pikp-2 with Avr-Pia". This comparison is not included in the figure in any case. If the point is that this low level of response may not translate into resistance as pikp does not provide resistance to strain with AvrPia, then perhaps it would be more appropriate to elaborate on this in the discussion?

The reviewer is correct in their statement that the point is that this low level of response may not translate into resistance as Pikp does not provide resistance to strains with Avr-Pia. To increase clarity, we have expanded this sentence in the results (starting line 253 in the revised manuscript) to include "To test whether the Pikm-1^{RGA5} chimera is a functional receptor, the Pikm-1^{RGA5}/Pikp-2 combination was co-expressed with AVR-Pia. Co-expression of Pikm-1^{RGA5}, Pikp-2 and AVR-Pia in *N. benthamiana* resulted in weak cell death, significantly weaker than the cell death elicited by RGA4/RGA5 in response to AVR-Pia (Figure 3 – Appendix 1 G), but comparable to the cross-reactivity of Pikp-1/Pikp-2 with AVR-Pia previously observed in *N. benthamiana* (Varden et al., 2019, indicating a level of cell death equivalent to a known interaction which does not translate into full resistance to M. oryzae carrying AVR-Pia in rice plants".

Furthermore we've highlighted this in the discussion at Ln 438 in the revised MS, inserting the text "Indeed, the cell death observed is better compared to the cell death caused by the Pikp pair when co-expressed with AVR-Pia, which has described as cross-reactivity in *N. benthamiana*, as it known Pikp is unable to provide full resistance to *M. oryzae* strains carrying AVR-Pia (Varden et al., 2019)."

Line 270. The similarity in AvrPia recognition between the RGA5 and APB is consistent with the different binding sites for AvrPia and AvrPik isn't it?

We agree with the reviewer and this is discussed in the paragraph starting Ln 409 in the discussion (now In 430 in the revised manuscript). We do not embellish too much on the idea of a different binding sites as our favoured hypothesis is recognition is due to lower affinity, and this is in-line with the incorporation of completely different domains (the VHH nanobodies) being capable of changing the NLRs ability to responding to entirely new ligands.

Line 282 onwards. While the lower affinity of RGA5-HMA for AvrPia is a reasonable explanation for the weaker HR response, another possibility to consider is that the different binding site is simply not located in the right position to favour the active state of the Pik-1/2 receptor complex over the inactive state. The data in Table 1 is quite important for this section and I think it would be better as a main table rather than a supplementary table.

We appreciate the reviewers insightful comment. However, we believe the manuscript is more coherent with Table S1 remaining in the supplement. As this study contains a considerable amount of data, it is a challenge to decide what information should be in the main manuscript to best convey the message to the reader, and as such we believe the current six figures in the MS best portray this information.

Line 347/Figure S10. I couldn't see figure S10, but I think it might be hiding behind figure S11 superimposed on top of it.

We are sorry the reviewer was unable to view the figure. Have checked the submitted version of the pdf and there appears to have been an error in the pdf conversion process. We have attached a corrected version of the pdf which correctly displays figures S10 and S11.

Line 354 - this sentence describing the autoactive nanobody fusions could go into the introduction - see comment above.

We believe our addition to the introduction as per the reviewers first comment covers this, while it does not explicitly name VHH nanobodies, it covers the essential information that manipulation of integrated domains can result in autoactivity.

Line 367. Worth noting that LaM3 was still autoactive even with pikp-2.

We have edited Ln 361 in the revised MS to read "While Pikm-1^{LaM3} remained autoactive, the autoactivity of Pikm-1^{LaG24}, Pikm-1^{LaM2} and Pikm-1^{LaM6} could be mitigated by co-expression with Pikp-2, while still retaining recognition of their cognate fluorescent protein, although in the case of Pikm-1^{LaM2} cell death signalling was significantly reduced (Figure 6; Figure S11 – Appendix 1 J).

Line 399. This sentence is not entirely clear. "different threshholds" for what?

To improve clarity we have edited this sentence at Ln395 in the revised MS to read "This co-adaptation may have led to different sensitivity thresholds of the helpers for the sensors, resulting in differences in the requirements for activation between Pikp-2 and Pikm-2."

Line 412. The authors state that the pikm1-/pikp-2 pairing is superior to either a pikp-1/pikp-2 or pikm-1/pikm-2 pair for engineering. However there is no data in this manuscript for engineering of the pikp-1 HMA domain or its compatibility with either pikp-2 or pikm-2. The only related data I can see is in figure 2A where pikp-1 containing the mHMA domain is shown to function equally well with either pikp-2 or pikm-2. Have other modifications of pikp-1 been described elsewhere as leading to autoactivity? If so, make that statement explicitly and include a citation, but if not then pikp1/2 should not be excluded as an unsuitable pairing.

We agree with the reviewer and have removed Pikp-1/Pikp-2 form the text. This sentence at Ln407 in the revised MS now reads: Combining the Pikm-1 sensor with the Pikp-2 helper yielded a compatible receptor pair with greater ability to accept HMA modifications than the natural pairing of the Pikm-1/Pikm-2 alleles.

Line 416. Insert that the pikm-1-VHH-nanobody fusions with autoactive in the presence of pikm-2

We have edited this sentence as per the editors recommendation. It now reads "Notably, this strategy appears to be useful in areas such as the incorporation of VHH-nanobody fusions into Pikm-1 to allow for tailor-made NLRs deemed 'Pikobodies' (Kourelis et al., 2023), which are often autoactive in the presence of Pikm-2."

Line 448 to 454. This could be rephrased as the logic is not consistent. First it is noted that the RGA5 and APB mutations give low levels of cell death and a possible explanation provided that the binding interface is occluded. Then it is stated that it is more likely due to the lower affinity because low levels of cell death were observed. Clearly that observation does not discriminate between these possibly explanations. Fine to suggest that low affinity is the reason, but that is based on the correlation of the response with the measured binding affinities.

The reviewer makes a valid point that low affinity could be a result of occlusion of the interface, as such we have rephrased the paragraph to address this, adding several more lines to strengthen our argument and make our speculation less vague. The text starting Ln446 of the revised MS now reads:" It is possible the AVR-Pia/AVR1-CO39 interface is occluded in the Pikm-1^{RGA5} chimera, and co-IP with the APB mutant did not show association in planta. However, we speculate the lack of AVR-Pia recognition *N. benthamiana* is likely due to a naturally lower affinity of the effector for the HMA domain opposed to occlusion of the interaction interface, as we were able to observe some weak cell death when Pikm-1^{RGA5} or Pikm-1^{APB} co-expressed with Pikp-2 was challenged with AVR-Pia. Indeed, substitution of VHH nanobodies that share no homology to the effector recognition interfaces of HMA domains are able to act as sensory domains for the Pikm-1 sensor, making the hypothesis that reduced response to AVR-Pia by the Pikm-1^{RGA5} and Pikm-1^{APB} receptor is due to the RGA5/AVR-Pia interface is occluded less likely. In support of this, previous studies have benchmarked the affinity of AVR-Pia/AVR1-CO39 for the RGA5 HMA domain in the micromolar range (Guo et al., 2018; Ortiz et al., 2017), while AVR-Pik effectors bind their cognate integrated HMA domains with nanomolar affinity (for interactions that result in cell death responses). These previous observations were corroborated by the biophysical analyses performed in this study (Figure S6 A, Table S1), which also measure affinities of RGA5 and the APB HMA domains for AVR-Pia in the micromolar range, compared to the nanomolar affinities of the APB HMA and Pikm-1 HMA domains for AVR-Pik effectors. It remains unclear why the binding affinities of the Pik HMA and RGA5 HMA domains for their cognate effectors differ so significantly in nature."

Line 509, figure 1 legend. The legend for part B does not describe what is shown in the figure - I assume UV fluorescence images of leaves at some time point after Agroinfiltration. As noted above the 434 and 627 mutations are not mentioned in the text or the legend.

We have edited the figure legend to address the reviewer's points. From Ln 510, the legend now reads "B) *N. benthamiana* cell death assays of Pikp-1$^{\Delta HMA}$ co-expressed with Pik-2 helpers and mutant variants visualised via UV light. The HMA domain of Pikp-1 is not required for effector-independent cell death in *N. benthamiana*; compatibility remains with Pikp-2 but not Pikm-2. Reciprocal D230E and E230D mutations in Pikp-2 and Pikm-2 flip the compatibility of the helper NLRs for Pikp-1$^{\Delta HMA}$. The other polymorphic residues between Pikp-2 and Pikm-2, T434S and M627V, have no effect on helper compatibility."

Reviewer #3 (Comments for the Author):

Authors opened a possible strategy for NLR-mediated resistance engineering by using the allelic diversity in Pik NLR pair to create receptors with expanded recognition specificities without causing constitutive cell death. Authors' structural, biophysical and in planta analyses clearly demonstrated a usefulness of the Pik system as a simple but efficient means for generating bespoke resistance. In addition, this work provides a clear basis for the incorporation of new effector recognition motifs into the Pik system. I believe that this work is well organized with convincing results and written very well. The reviewer would like to confirm several minor points.
(1) Figure 1B: it would be nice to add a brief description about the reciprocal mutations for the two other residues T434S and M627V in the main text.

We have updated the figure legend as per the reviewer's comment and added some text to the results addressing these. See responses to reviewer #2 comments 2 and 16.

(2) L229: Our results also suggest Pikp-2 may be more accommodating of changes in the Pik-1 sensor than Pikm-2, even tolerating the complete substitution of the integrated HMA by an unrelated domain without inducing autoactivity. Here, cite the result.

We have cited the result (Figure 1 – Appendix 1A) as per the reviewers request.

(3) Fig 6 B: I wonder if the Pikp-2 + Pikm-1-LaM6 results are correct (Supplementary S11 showed cell death in Pikm-1LaM6 + Pikp-2 + mCherry, which is not consistent with Fig 6. Adding a control experiment (absence of fluorescent proteins) would be better here. Also, I would expect some discussion on this issue about how to overcome such problems in engineering Pikobodies.

We thank the reviewer for their observant comment – the issue was the arrangement of the figure 6 B. The Lam6 does indeed respond to mCherry however, the panels for the mCherry and eGFP coinfiltration with the helper were incorrectly arranged. This has been updated in Figure 6 and now corresponds correctly with the cell death quantification reported in S11.

(4) Check the S10/S11 figures.

We have acknowledged and dealt with this in response to Reviewer #2.

TPC2023-RA-00251R1 2nd Editorial decision – *acceptance pending* June 5, 2023

We are pleased to inform you that your paper entitled "Allelic compatibility in plant immune receptors facilitates engineering of new effector recognition specificities" has been accepted for publication in The Plant Cell, pending a final editorial review by a science editor. At this stage, your manuscript will be evaluated by a science editor with respect to its presentation of scientific content, compliance with journal policies, and presentation for a broad readership.

Final acceptance from Science Editor July 10, 2023
